# Street dance redefined: a bridge across the knowledge gap

**DOI:** 10.3389/fspor.2025.1610656

**Published:** 2025-07-23

**Authors:** Xi Ling

**Affiliations:** Faculty of Education, Silpakorn University, Nakhon Pathom, Thailand

**Keywords:** street dance, hip-hop dance, breakdance, define, knowledge gap

## Abstract

**Introduction:**

Street dance research has expanded rapidly since its inception, yet the absence of a unified terminology has produced inconsistent academic interpretations and a substantial knowledge gap. This study aimed to establish a clear definition of “street dance,” classify its common styles, and identify the factors underlying terminological inconsistency.

**Methods:**

Following PRISMA guidelines, we conducted a systematic review. Indicative Behavioral Standards were applied to critically appraise 26 peer-reviewed articles retrieved from major databases. Data extraction focused on terminological usage, style descriptions, and reported sources of conceptual divergence.

**Results:**

The review revealed wide variability in definitions and style classifications across the literature. Synthesizing these findings, we reconstructed a concise, consensus-based definition of street dance and proposed a classification framework for common street dance styles. This review identified internal and external factors contributing to the knowledge gap. Internal factors including individual creativity, knowledge transmission. External factors including national cultural, media communication, music development, public opinion.

**Discussion:**

The reconstructed definition and classification system standardize street dance terminology, offering researchers, educators, and practitioners a coherent conceptual framework. This advancement enhances scholarly communication and supports more rigorous future investigations within the global street dance community.

## 1 Introduction

In the Lau (1), which records the words and teachings of Confucius and is considered the most reliable expression of Confucianism, the text reads:

To know what you know and what you do not know—that is true knowledge (1).

This statement is one of the most important principles when defining the concept of street dance. So what way can help street dance researchers define the concept of street dance? Exploring the classification of common street dance styles and investigating the factors that influence public perception of popularity may be two feasible approaches.

In fact, in existing research on the definition of street dance, scholars have different understandings of the terminology used to define street dance, resulting in a significant knowledge gap. As Gaziano (2) stated, “A knowledge gap may not exist for awareness of an issue but may exist for in-depth knowledge of that topic” (p. 1). For example, Stevens (3) noted that “street dances—the popular dances of black America” (p. 361). Lys Stevens limited street dance term to the African American community, assigning it two labels: black community and popular dance. In another example, Lai et al. (4) described street dance as an “informal dance style” that takes place in public places (p. 2). Li in Vexler (5) combined the above two examples of street dance research and describes street dance as a popular dance with more diverse dance styles:

The term “street dance”, especially prevalent in Europe and Asia, is used to encompass all of the above, as well as waacking, house, krumping, and the “hip-hop” style. as waacking, house, krumping, and the “hip-hop dance” that during the past decade has begun to be developed as a discipline of its own. (p. 440)

Street dance researchers assigned three elements to the definition of street dance, including: black community, informal dance, and numerous styles (3–5). This view have also been widely cited by other scholars in academia (6–8).

It is worth noting that although street dance as described by Li in Vexler (5) encompasses a wider range of styles, However, Li in Vexler (5) seems to lack an objective understanding of street dance style. For example, Li in Vexler (5) described breakdance (a style of street dance) as follows:

B-Boys, B-Girls, and everyone involved in hiphop culture never before used the term “breakdance” to describe what they do, so even today when someone uses the word “breakdance”, it signifies a lack of understanding about this dance and its culture. (p. 435)

Whether it is B-boy or breakdance, different understandings arise in different cultural contexts. In fact, other researchers have also explored this issue, for example, Yang et al. (9) mentioned that using a single name may lead to missing literature. Because some scholars may use street dance or other terms to refer to breakdance. In other words, Yang et al.'s study indirectly demonstrated the diversity that exists in the terms of street dance. This also responds to Sotiriadou in Hill (10) that, terminological ambiguities can lead to ambiguous claims that result in statements that are subject to multiple interpretations, which in turn affects the validity of research findings. It can be seen that knowledge gap refers to the cognitive bias of street dance scholars caused by the confusion of the definition of street dance.

I agree with Yang et al. (9) that failure to establish a unified definition of street dance will result in the following negative consequences: (1) confusing the street dance terms, which affects the validity of academic research and authority, hindering the development of the academic system of street dance; (2) misleading the implementation of street dance in education and cultural policies, affecting the social acceptance of street dance; and (3) misleading subsequent scholars in their research, resulting in the expansion of the cognitive misconceptions of street dance scholars.

Given these considerations, how can I describe the definition of street dance terms? Additionally, how can I identify the influencing factors that cause the knowledge gap in street dance? This review attempts to describe definition and categorization to standardize the public's perception of street dance and provide reference theory for street dance research. In addition, this review explained the influencing factors of the knowledge gap in street dance. In view of the existing literature studies, I briefly reviews the current status of the knowledge gap in street dance and aims to answer the following questions:

Research question 1 (RQ1): What is street dance?

Research question 2 (RQ2): What are the common street dance styles?

Research question 3 (RQ3): What are the factors that contribute to the public knowledge gap?

If I could answer the question, “What is street dance?”, I can better help street dance scholars to establish the foundation of unified street dance theoretical cognition.

First, this will reduce the academic misinformation caused by the knowledge gap of street dance cognition (11). Second, if I establish a categorization of street dance styles and an explanation of street dance styles, I can reduce the risk of cognitive conflicts of street dance terms. These conflicts are often caused by scholars from different regions due to different cultural backgrounds (5). In addition, establishing a causal model of the factors of the street dance knowledge gap will help the public to clearly understand the root causes of the street dance terms, and help bridge knowledge gap between teachers and students, and between scholars.

In this review, I proceeded in three stages. The initial stage focused on the methodological approach, which involved defining the criteria for literature inclusion and screening, followed by the extraction and subsequent analysis of the literature that met the established requirements. In the second stage, drawing on the screened literature, I collected data concerning the definitions of street dance terms, classifications, and the factors influencing the knowledge gap. Based on these data, I formulated and elaborated on hypotheses with the aim of reaching a consensus on the definitions, classifications, and influencing factors related to street dance. Finally, by analyzing the influencing factors, a causal model of the knowledge gap of street dance terms is developed.

## 2 Method: screening methods for research articles on street dance definitions

### 2.1 Search strategy

The review's selection process followed the Systematic Reviews and Meta-Analyses (PRISMA) Statement (12). The researchers used the PICo framework as a strategy for identifying search terms (13), “PICo stands for the population, the phenomena of interestand the context” (p. 181). I searched Scopus and Web of Science on April 7, 2025 for peer-reviewed articles on street dance definition studies. I adopted the search strategy of Yang and Whatman (14) and then optimized it according to the topic of this review, different permutations of each keyword based on previously validated search results. I used not only the term “Street dance” but also “Breakdance”, “Hip-hop dance” because street dance scholars may focus on related aspects of street dance, such as breakdance (15), popping dance (16), and other styles of street dance, but did not use the term “street dance”. However, the retrieved articles must contain content relevant to the definition of street dance (see Table 1 for keyword search).

**Table 1:** Terms used to search two databases related to qualitative research on street dance.

Database	Step	Terms	Results
Web of science	Search terms 1	ALL = (“knowledge gap” OR “Cognitive gap” OR “gap” OR “cognitive differences” OR “Cognitive” OR “understand”)	6,596,566
	Search terms 2	ALL = (“Street dance” OR “streetdance” OR “street-dance” OR “Street Dance” OR “street dances” OR “streetdancing” OR “street dancing” OR “hiphop dance” OR “hip-hop dance” OR “Hiphop dance” OR “Hip-Hop dance” OR “HipHop dance” OR “b-boying” OR “b-girling” OR “urban dance” OR “breakdance” OR “break dance” OR “breakdancing” OR “breaking dance” OR “Piliwu” OR “jiewu” OR “popping dance” OR “Boogaloo” OR “Locking dance” OR “Cambell lock” OR “New Jack Swing” OR “Waacking” OR “Punking” OR “Krumping” OR “House Dance” OR “Dance House” OR “House Dancing“ OR “Litefeet” OR “Crip walk” OR “Crip walk” OR “C-walk” OR “Old school” OR “New school”)	6,933
	Search terms 3	ALL = (“Breakers” OR “b-boy” OR “b-girl” OR “breakdancer” OR “breakdancers” OR “b-girls” OR “b-boys” OR “Hip-hopper” OR “hip-hoppers” OR “breakers” OR “breaker” OR “Hip-hop practitioner” OR “dancer” OR “dancers” OR “young people” OR “insiders”)	387,960
	1 AND 2 AND 3		56
Scopus	Search terms 1	TITLE-ABS-KEY (“knowledge gap” OR “Cognitive gap” OR “gap” OR “cognitive differences” OR “Cognitive” OR “understand”)	4,113,507
	Search terms 2	TITLE-ABS-KEY (“Street dance” OR “streetdance” OR “street-dance” OR “Street Dance” OR “street dances” OR “streetdancing”OR “street dancing” OR “hiphop dance” OR “hip-hop dance” OR “Hiphop dance” OR “Hip-Hop dance” OR “HipHop dance” OR “b-boying” OR “b-girling” OR “urban dance” OR “breakdance” OR “break dance” OR “breakdancing” OR “breaking dance” OR “Piliwu” OR “jiewu” OR “popping dance” OR “Boogaloo” OR “Locking dance” OR “Cambell lock” OR “New Jack Swing” OR “Waacking” OR “Punking” OR “Krumping” OR “House Dance” OR “Dance House” OR “House Dancing” OR “Litefeet” OR “Crip walk” OR “Crip walk” OR “C-walk” OR “Old school” OR “New school”)	5,962
	Search terms 3	TITLE-ABS-KEY(“Breakers” OR “b-boy” OR “b-girl” OR “breakdancer” OR “breakdancers” OR “b-girls” OR “b-boys” OR “Hip-hopper” OR “hip-hoppers” OR “breakers” OR “breaker” OR “Hip-hop practitioner” OR “dancer” OR “dancers” OR “young people” OR “insiders”)	191,038
	1 AND 2 AND 3		38

### 2.2 Inclusion and exclusion

**Figure 1:**
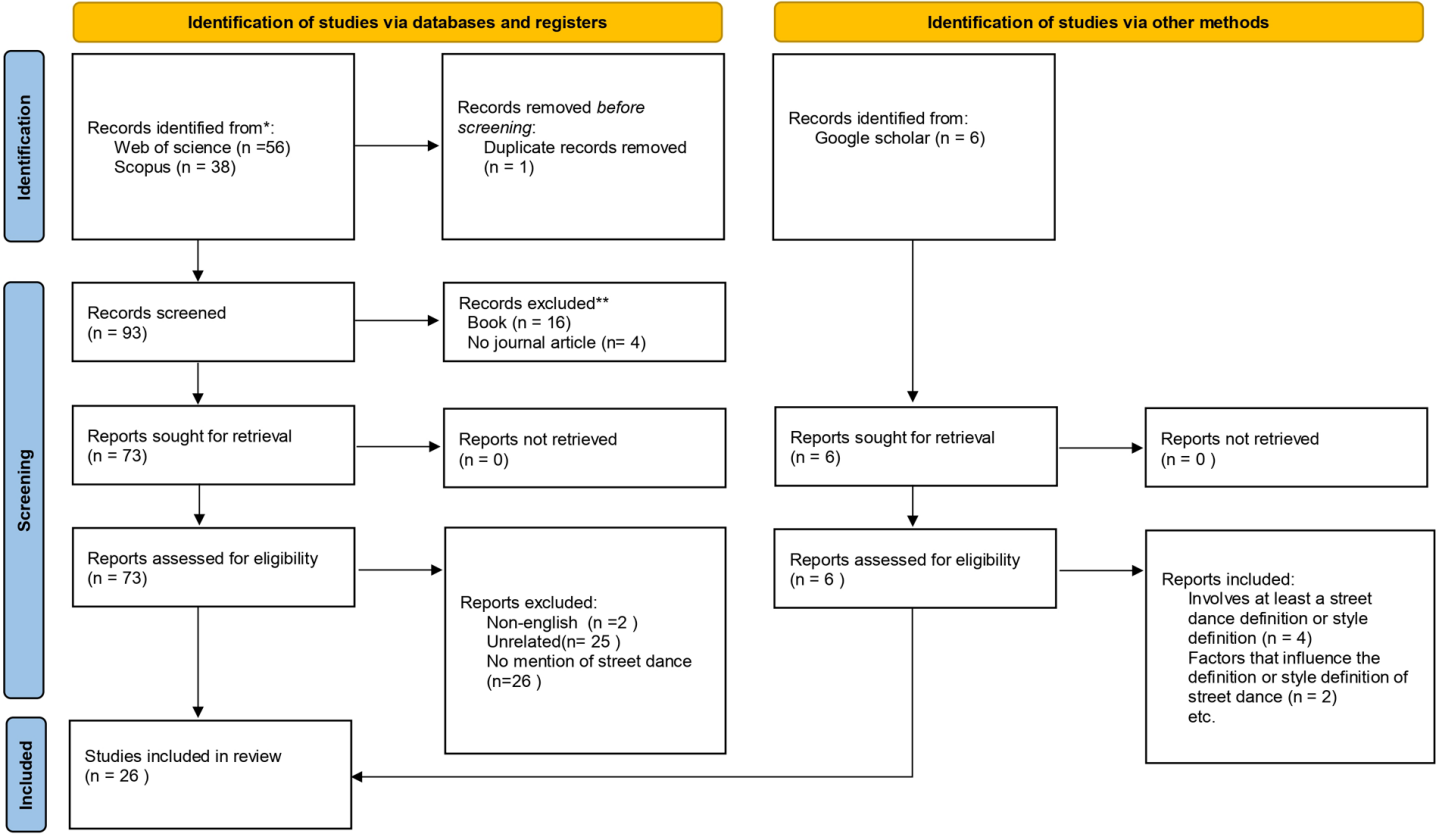
PRISMA flow diagram for article selection.

I used a series of inclusion and exclusion criteria to screen articles related to the definition of street dance. Inclusion criteria included (1) peer-reviewed journal articles written in English; (2) the study included, but was not limited to, the term street dance, e.g., words such as hip-hop dance (see Table 1: keyword search for more details); and (3) the participants including street dancers or individuals who had participated in street dance activities. If the study was a non-street dance study, such as K-pop (popular dance from Korea), it was excluded because it was not relevant to street dance. The initial search identified a total of 56 articles in Web of Science and 38 articles in Scopus, which were imported into the Zotero reference management software. I used Zotero to conduct a literature screening process and excluded 21 articles from the 94 articles screened. Types of literature excluded include: non-English literature, books or book chapters, conference papers, dissertation, and duplicates. After a full-text review, I excluded 53 articles that were considered to be irrelevant to the street dance topic because they simply mentioned street dance and did not discuss it in depth. To avoid omission and neglect of some important literature, I also found another 6 articles from Google Scholar. Ultimately, 26 articles were included in the final review sample. Figure 1 further depicts the inclusion/exclusion process.

### 2.3 Extraction and analysis

To maximize the transparency and traceability of the extracted literature, I listed the basic structure and relevant evidence of all articles that met the inclusion criteria, including: Author, year of publication, study design, methods and data collection, phenomena of interestand, population, and context. Summary of the reviewed articles see Supplementary Material A.

The data extraction and analysis procedures followed the steps proposed by Yang and Whatman (14): (1) the PDF versions of the 26 articles included in the review were saved in a folder named “review”; (2) these articles were read thoroughly; (3) the main contents from the PDF files were transferred to Microsoft Word and summarized; (4) a coding framework was developed based on the assessment criteria (14) and our research questions to evaluate the quality of the literature and to provide answers to the research questions posed in this study; (5) the 26 articles were coded in Word; (6) the coded content (e.g., quotations and references) was documented in a Microsoft Excel literature grid; (7) the tag scores (e.g., non-compliant with purpose statements—0) from Excel were entered into a Guttman chart (17), see Figure 4. The original data, coding framework, records of the coding process (e.g., codes, subcodes, and direct quotations), and descriptive information of the 26 articles are provided in the Supplementary Materials B.

## 3 Quality appraisal

I used Yang et al.'s study on Indicative Behavioral Standards to assess the quality of the literature (14). This choice was made because this criterion is specifically designed to assess the quality of breakdance (a classic style of street dance) literature. As Zhi Yang stated,

What distinguishes our evaluation standards from others is that they are specifically designed for qualitative research in the breakdance field, marking a pioneering effort in the scholarship. Additionally, our standards focus on providing feedback and guidance (14).

**Figure 2:**
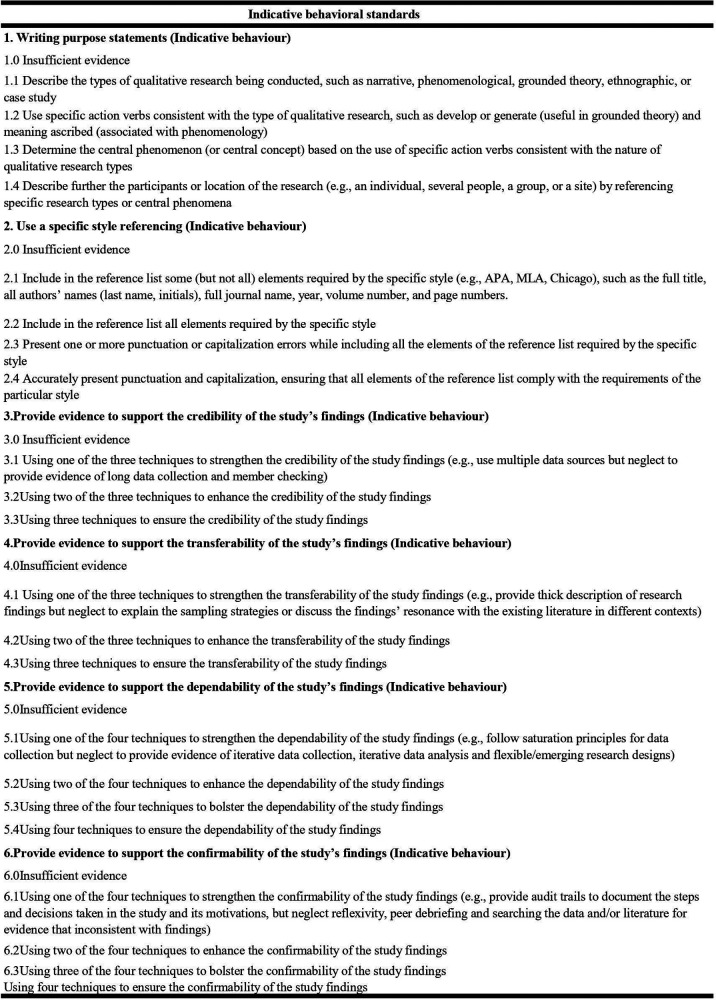
Indicative behavioral standards. From/Adapted from “Development and validation of standards for evaluating the quality of qualitative research on Olympics breakdance” by Yang et al. (14).

**Figure 3:**
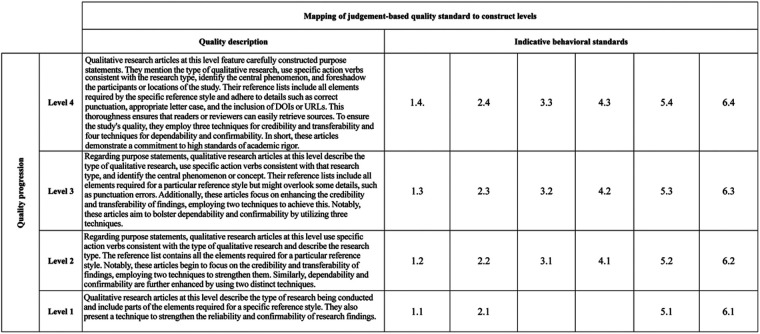
Hypothesized construct map. From/Adapted from “Development and validation of standards for evaluating the quality of qualitative research on Olympics breakdance” by Yang et al. (14).

Indicative behavioral standards consist of 6 dimensions that assess the quality of street dance literature. These items are indicated by a rating on a level of 1–4. Figure 2 provides the indicative behavioral standards, Figure 3 provides the level map.

**Figure 4:**
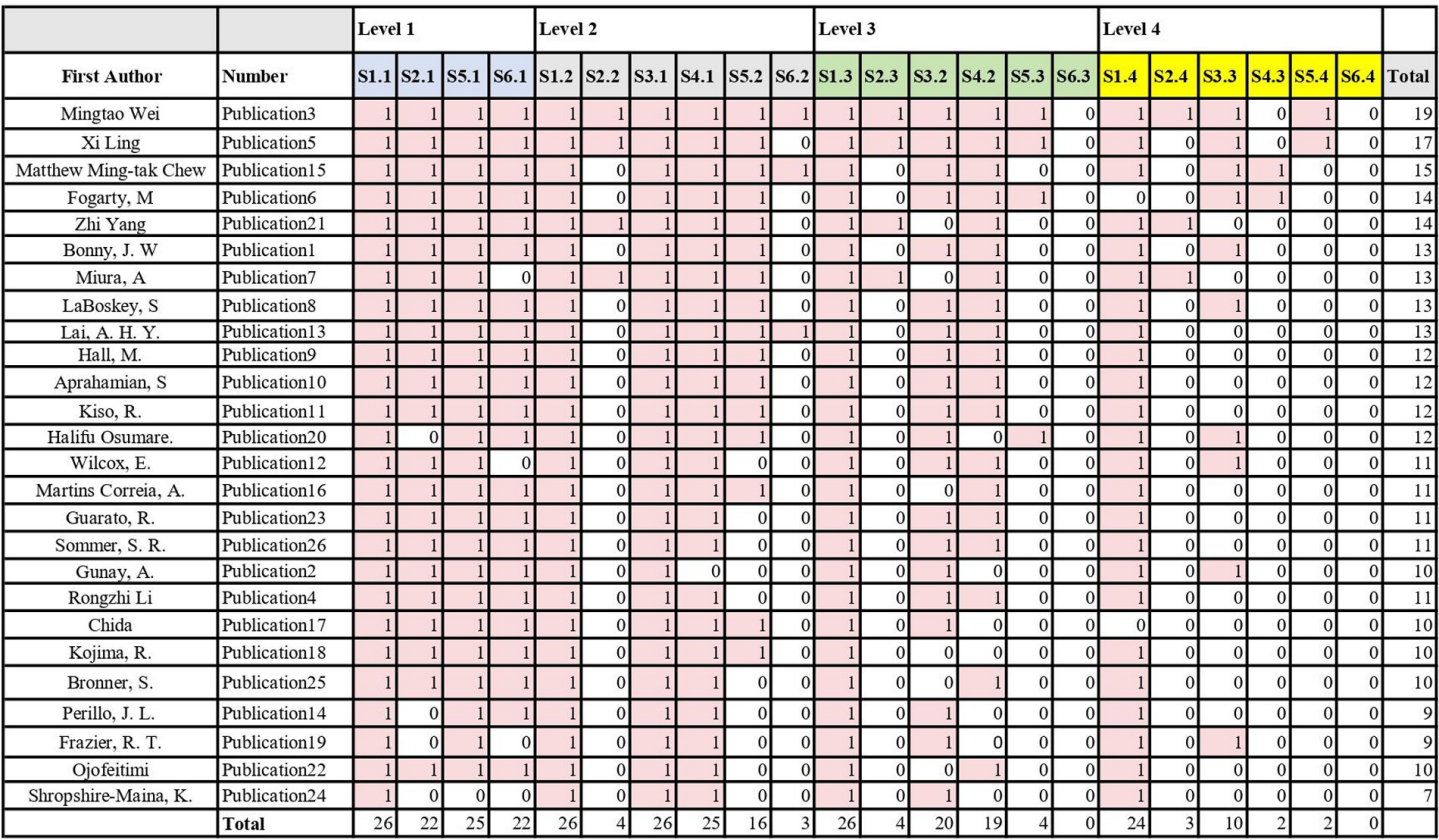
Data entry. At the top of the grid are the standards labeled as 1.1, 2.1, 5.1, 6.1, and so on, with this pattern continuing through to standards 5.4 and 6.4. The descriptions of the standards are typically lengthy, which may make them difficult to read within the cells of an Excel spreadsheet; however, I have documented each standard meticulously. The colors blue, gray, green, and yellow denote different levels of standard groups within the hypothesized construct map.The total scores for each publication and for each standard are calculated at the end of each row (publication total score) or column (standard total score). To enhance the clarity of the standard score patterns, I have changed the color of the cells containing “1”s to red.

The quality of the collected literature was rated using Standards (14). For example, the first dimension in Indicative Behavioral Standards is Writing Purpose Statements. Sub-items 1.1–1.4 (S1.1–S1.4) earn 1 point for meeting criteria, 0 points for not meeting. The remaining five dimensions follow in sequence. The reliability of the selected literature was assessed using Indicative Behavioral Standards (14), with results detailed in Figure 4. The Raw Data Set is available at Supplementary Material B.

## 4 Result

As can be seen from Figure 4, the quality assessment of publications about street dance. The dataset consists of 26 publications, each of which is scored and the total score for each publication is calculated. The total scores for the publications range from 7 to 19.The highest- scoring publication (Publication3) achieved a total score of 19, the lowest-scoring publication (Publication24) achieved a total score of 7.

The publications demonstrate consistency in describing the type of research being conducted and using specific action verbs, with 22 out of 26 publications receiving full scores. However, the variability in scores at Levels 2, 3, and 4 indicates areas for improvement, particularly in documenting the decision-making process and motivation in the research, peer review, inclusion of all elements of the reference list that meet specific formatting requirements, use of two or more techniques to improve the transferability of the research, and provision of iterative scores. However, the variability in scores at Levels 2, 3, and 4 indicates areas for improvement, particularly in documenting the decision-making processes and motivations in the study, peer review, inclusion of all elements of the reference list that meet the specific format requirements, the use of two or more techniques to improve the transferability of the study, and the provision of iterative data collection.

To ensure reliability and transparency in the process of assessing the quality of the literature, I used a reflexive diary to identify any inconsistencies that may have arisen during the assessment process (18), as detailed in Supplementary File C. This reflective practice helps the reader to get a clear picture of the research quality of the literature. In addition, I meticulously maintained an intellectual audit trail at all stages of the study to document the evolution of my thinking and decision-making (14), as detailed in Table 2.

**Table 2:** An intellectual audit trail on street dance terminology and history.

The study to document	Thinking and decision-making
Starting philosophical position	When I was ready to begin this research, my philosophical paradigm was based on positivism. While pursuing my Master's degree, my research focused on street dance, and the knowledge I gained of street dance terminology and history came from street dance positivism research and oral transmission from street dancers.
Questioning the positivist position	After becoming a PhD candidate, I began to recognize the limitations of positivism research. While existing empirical studies of street dance have described the terminology and history, street dance terms are often not uniformly named, and some literature even shows misuse of terms. Based on the above, this may create misunderstandings about the history of street dance and the understanding of the terminology; for example, the same historical event that gave rise to the street dance term may result in audiences in different countries assigning new names to the street dance term. A typical example is that breakdance is known as “PILIWU” in China and street dance is known as “JIEWU” (19). This phenomenon of oral instruction is not unique to China. In fact, the spread of street dance around the world has given rise to a variety of names, which has resulted in the lack of uniformity in the use of street dance terminology by current street dance scholars.
The search for a philosophical stance	In addition to extensive reading of books on street dance terminology and history, I drew on the research of street dance scholar (9, 15) and qualitative methodology scholar Sotiriadou (10) to use critical realism as my research paradigm. Critical realism assumes a transcendental realist ontology, an eclectic realist/interpretivist epistemology and a generally emancipatory axiology. By developing a structural map of street dance terminology, I hope to help street dance researchers create a unified standard of terminology. I also hope that a unified street dance terminology will allow street dance educators to better design street dance materials, conduct street dance instruction, street dance competitions, and more easily promote street dance events. My approach focuses on applying a unified street dance terminology to the academic field of street dance in order to address the confusion of street dance terminology, a perspective that is consistent with the philosophy of critical realism.
Considering alternatives for evidence collection and data analysis	Despite the methodological flexibility provided by the critical realism paradigm, I faced challenges in reviewing the diverse literature. For example, the lack of a uniform street dance terminology vocabulary and the need to set up search terms that often required the use of ten or more keywords (see Table 1 for more details) made reviewing the literature significantly more difficult. For data collection, I chosed the PRISMA search strategy to maximize the transparency and reproducibility of the review. This strategy required researchers to report in detail the search terms, database, and inclusion/exclusion criteria.
Interpretation and presentation of evidence	The interpretation of the data in this study is guided by three primary research questions: 1) What is street dance? 2) What are the common street dance styles? 3) What are the factors that contribute to the public knowledge gap? To supplement the additional content in the charts, I used the text of a personal reflective journal and 26 literature code text.
Raw data	The process of evaluating the indicative behavioral standards of the literature (See Supplementary Material B), personal reflexive diaries (See Supplementary Material C), and Glossary of street dance terms (See Supplementary Material D).

## 5 Findings and discussion

### 5.1 Research 1: what is street dance?

Through a review of keywords in the abstracts of the existing literature, I identified the literature's focus on the definition of street dance. For example, street dance was defined as a cultural and artistic form of urban dance that emerged in public spaces as a form of self-expression and community interaction, or a type of street dance in which dancers can improvise according to the music, the environment and the audience (20), or “as an urban dance, is composed of various other genres originating in popular culture and street culture” (21). Regardless of whether these articles focus on the definition of street dance or the history of street dance, the articles reflected the following claims about the definition of street dance:

(1) Street dance is a form of urban dance rooted in public space, which is characterized by the ability to integrate one's personal understanding of street dance with the local cultural context to create new styles. Street dance emphasizes free creation, and Freestyle is a key factor in its development.
(2) Street dance is an urban dance form that encompasses hip-hop dance and non-hip-hop dance. Common street dance styles divided into two periods: old school and new school. The old school includes styles such as breakdance (breaking, popping, locking), and waacking, while the new school features new jack swing, new style, house, krump, and other emerging styles. Street dancers often use choreography to creatively integrate these diverse styles, contributing to the dynamic and ever-evolving nature of street dance culture.
(3) Street dance is a kind of social dance in which people utilize street dance to make social connections with others. Cypher and battle is a form of impromptu sharing between street dancers and a platform for communication between street dancers. Communication between dancers is a key factor in the development of street dance.

In this section, I presented a series of cited literature from diverse backgrounds to illustrate the points made.

Regarding the first knowledge claim, Lai et al. (4) defined street dance content from the perspective of social environment. For example, Lai et al. (4) noted that “.Street dance is an informal dance style that takes place in public spaces, such as streets, parks, and dance parties” (p. 2). According to Lai et al. (4), street dance associated with socialization to promote emotional communication between people (Knowledge Claim 1). In terms of the freestyle, creativity is also part of freestyle. Yang et al. (9) explained the definition of creativity based on breakdance choreography. For example, Yang et al. (9) wrote “…the most distinctive feature of creative dance is that the expressive aspect is more important than the functional aspect…” (p. 2). Yang et al.'s perspective emphasized guiding students' free choreography of street dance moves and focuses more on students' ability to be creative using street dance moves (Knowledge Claim 1). Bonny et al. (22) further explained the classification of freestyle in terms of creativity. For example, Bonny et al. (22) claimed that “…some of which, such as break dancing, are more spontaneous and in response to music, and others that are more structured, pre-choreographed routines” (p. 20/26). Here, Bonny et al. (22) categorized freestyle into improvised and purposefully choreographed dancing, and creating moves that surprise the audience becomes an indicator of high street dance competence (Knowledge Claim 1).

With regard to the Knowledge Claim 2, the concept of street dance should encompass an explanation of the term street dance. Categorized in terms of time, street dance terminology encompasses old school and new school (23). Perillo (23) stated that the relationship between old school street dance and new school street dance is cultural transmission relationship, dancers transmit knowledge of old school and new school street dance. For example, new style, a classic street dance style, “.…the Elite Force came up with their whole DVD thing and their whole old school new school Hip-hop dictionary” (p. 82). Thus the division of street dance into old school and new school can help dancers better understand street dance terminology. However, this idea made the terminology of street dance limited. Because street dance possesses multiple styles (24–26), such as breaking, popping, locking, waacking, just to name a few. Therefore, street dance is a kind of urban dance that contains many styles. In addition, dancers expressed different styles of street dance through choreography, creating street dance movies that are well known all over the world, such as the 1984 movie Breakin’ (19, 27). An unavoidable question is, how does hip-hop dance relate to street dance? As an extended discussion of theories related to hip-hop is impractical for reasons of space, I have compiled the historical literature on hip-hop and invite interested readers to read it (28–30). To help readers understand the classification of street dance, in the next section, I have designed a classification framework for street dance styles from different periods based on Knowledge Claim 1.

Regarding the Knowledge Claim 3, it is necessary to explain the meaning of Cypher (as know as Cipher). According to Wei et al. (31),

Cipher is dynamic … Cipher can occur in many formal and informal learning environments, such as on campus, on the street … Cipher is shared. the core idea behind cipher is really seeing all the fantastic innovations that are happening … (p. 04).

In addition, battle also possessed communicative attributes but is also confrontational in nature (32). Osumare (33) argued that “Breakdance ‘battles’ originally took place in lined-up opposing ‘gangs’ facing each other” (p. 33). Nowadays, battle is no longer a confrontation between gangs, battle has become a mode of sports competition, selected for the 2024 Paris, France Olympics (34). It can be seen that cypher and battle have contributed to the street dance's worldwide widespread popularity, the sharing of new idea have facilitated the development of the street dance movement (35), which is in line with Wei et al. (15) “Principles of hip-hop: Peace, unity, love, having fun, and knowledge” (p. 6).

### 5.2 Research 2:what are the common street dance styles?

**Figure 5:**
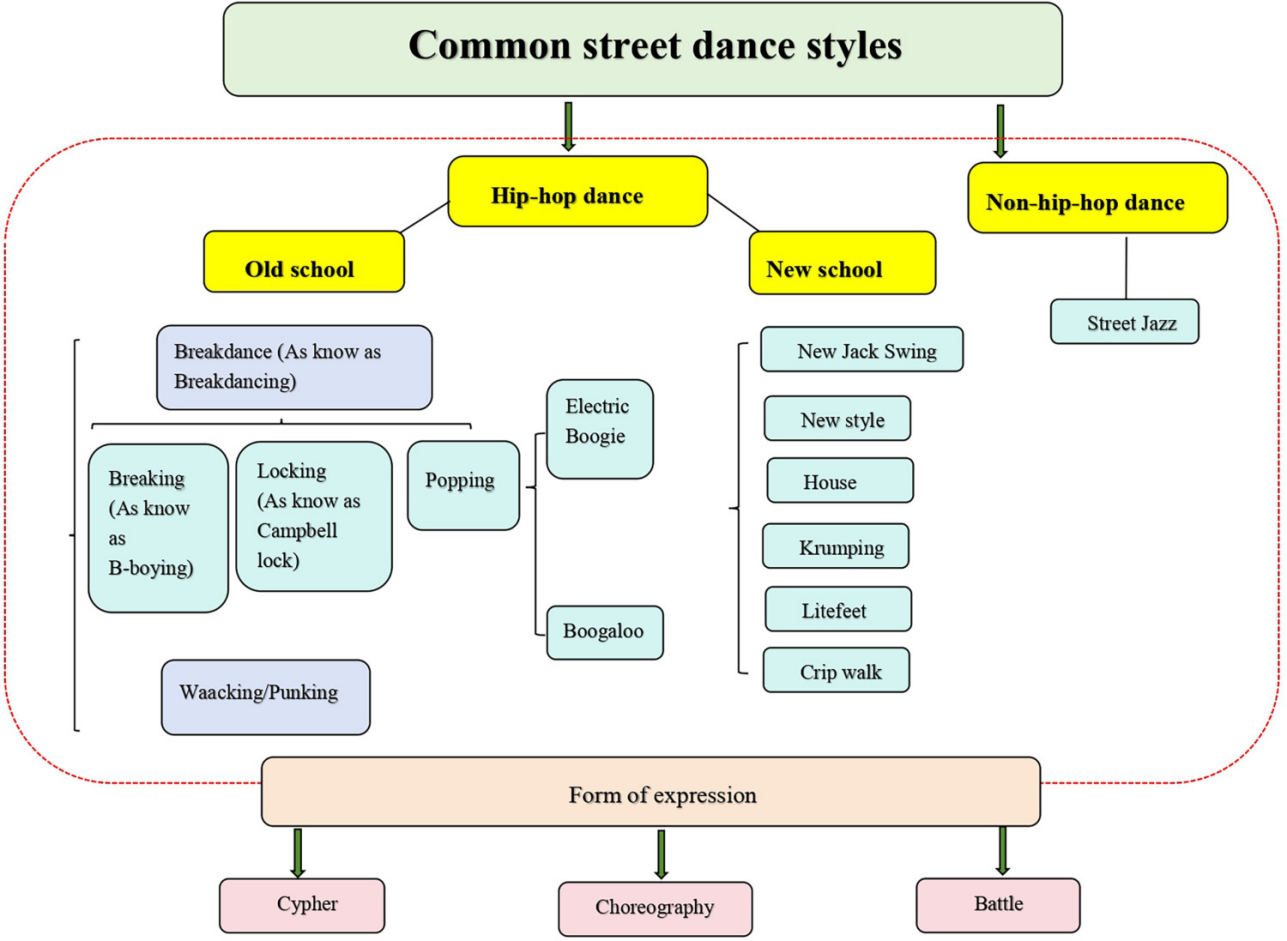
The common street dance styles. The figure showed the categorization of common street dance styles into hip-hop and non-hip-hop dance categories. In addition, common street dance styles are categorized by cypher, choreography, and battle as forms of expression.

This section focused on the categorization of street dance styles. From the perspective of temporal development, common street dance is divided into two periods, old school and new school. Old school period is considered to be between 1970 and 1980, while new school is considered to be between 1980 and 1990 (36). However, Ojofeitimi et al. (37) argued that “Old School dance styles emerged in the 1970s and 1980s (e.g., breaking, popping, and locking) … New School styles (e.g., house, krumping, street jazz) developed in the mid-1980s to 1990s as hiphop evolved” (p. 347). It is noteworthy that the two perspectives create a middle ground, making it possible for the street dance styles produced during this period to be both old school and new school in nature. In the following, I have provided a detailed classification of the street dance styles commonly seen during these two periods, see Figure 5.

#### 5.2.1 Old school street dance

It is well known that old school street dance took place in the 1970s and 1980s (37).Breakdance, as know as breakdancing, a classic hip-hop dance (9), known worldwide for the movie Breakin’, encompasses breaking (b-boying), locking, popping (15, 16, 38). Osumare (33) argued that “.…breakdancing originated as a creative dance alternative to actual gang violence, as well as party moves in the percussive breaks of the early 1970s hip hop mix of funk, soul, disco, and salsa music” (p. 33). Wei et al. (15) suggested that breakdance originated a little earlier, “Breakdancing originated in the United States between the 1960s and 1970s” (p. 5). After defining the time of old school street dance, I specified the detailed styles.

Breaking, the oldest style of breakdance, breaking men are called b-boys and women are called b-girls (5). Kopytko (39) defined breaking as “… the athletic floor-based movements often resembling acrobatics…” (p. 21).It must be recognized that acrobatics are one of the most common features of breakdancing, but that doesn't cover the full spectrum of breaking. Subsequently, street dance scholars have offered broader definitions. Vexler et al. (40) noted “Breaking is a very improvisational and creative dance” (p. 2). Laboskey (41) noted that “Breaking is most often defined as ‘downrocking’, which Tricia Rose describes as a “competitive, acrobatic and pantomimic dance with outrageous physical contortions, spins, and back-flips…” (p. 113). As we can see, breaking is not a purely aerobatic style of street dance, but rather an ancient form of street dance that combines both dance moves and technique. To summarize, breaking is a form of street dance that originated in the Bronx, New York, in the 1970s. Influenced by figures such as Bruce Lee, it incorporates acrobatics and elements of Kung Fu, and is closely associated with hip-hop culture. The signature moves of breakdance include: power moves, freezes and toprock/footwork, the commonly used music genre is breakbeat.

In the media, breaking has been misrepresented as breakdance to the extent that other breakdance styles have been overlooked (38). Next, I discussed these two styles.

Locking, a classic breakdance form often used by street dancers to perform, was originally known as campbell lock (19, 42). As locking has evolved, the name and definition has changed, Chida et al. (25) defined locking as “the movement in which a dancer moves and stops his or her hands and feet quickly. This dance was named ‘lock dance’ because the dancer seems to be locking by these movements” (p. 37). Lock dance is characterized by fast, stop-start movements, robot-like gestures, and complex movement control and coordination through joints and muscles (25), complex movement control and coordination (43). Although the name of locking has changed, it retains the classic movements of locking and continues the witty and joyful dance style of campbell lock. Therefore, locking is defined as a street dance originating in the late 1960s, characterized by rapid movements, abrupt pauses, a joyful sense of rhythm, and dramatic expressiveness.

Popping has been referred to by some scholars as “Electric Boogie” (5) and shares cultural roots with locking (44). It is interesting to note that popping can denote both a general term and a style of dancing (a style of dancing with rapid muscle contractions) (16). Laboskey (45) categorized popping dance, including:Electric Boogie and Popping. This categorization seems to overlook an important style of popping dance, Walker (46) argued, “‘Popping’ has come to be known as an umbrella term for many dance styles that have been grouped together such as; boogaloo, scarecrow, ticking, waving and many more” (p. 32). From this, it can be seen that popping dance includes popping as a communal element is integrated into both electric boogie and boogaloo styles. Therefore, I defined popping dance as a hip-hop dance form that originated on the West Coast of the United States in the 1970s, with quick muscular contractions as its signature movement. The diverse technical styles include electric boogie (e.g., robot, slide, waving, etc.) and boogaloo (Scarecrow, Romeo twist, etc.). Funk and electronic music are commonly used in popping.

Despite our definition of the three styles of old school street dance, there are still some old school street dance that have been left out (23). In fact, waacking is considered to be in the same category as breakdance as old school street dance (20, 33, 36, 47, 48).Waacking, as know as Punking. Bragin (49) noted that “‘Waacking/Punkin’ developed in gay, primarily Black and Latino underground disco clubs of 1970s Los Angeles” (p. 63). Waackin/punking dancers spread this popular dance to the general public on the television program Soul Train (50), which along with breakdance is known as old school street dance.

Although hip-hop scholar Hazzard-Donald (20) ranked old school street dance, and waacking is thought to have emerged before breakdance, there is not yet enough evidence to prove which style emerged first. Therefore, this review only listed the categorization of old school street dance and will not discuss the order of its emergence. To summarize, old school street dance includes breakdance and waacking/punking. Because these styles are hip-hop dance, it is widely believed that old school street dance is actually old school hip-hop dance. Street dance dancers spread street dance around the world through the television program Soul Train, and as the music and street dance techniques developed, street dance entered new school era.

#### 5.2.2 New school street dance

New school street dance is thought to have occurred in the mid-1980s to 1990s (37).Based on old school street dance, new school street dance has developed various styles, such as New Jack Swing, new style, house, krumping, crip walk, Litefeet (51). It is worth noting that new school differs from old school in that new school covers non-hip-hop dance urban dance, such as street jazz (52), which makes new school more diverse. Even after 2000, a form of street dance choreography called Hip-Hop Choreography emerged (53). Unlike old school street dance, new school street dance incorporates a variety of musical genres (e.g., hip-hop, R&B, electronica), focuses on choreography and improvisation, and spreads rapidly across social media platforms. I introduced the styles of new school street dance in the latter paragraph.

New jack swing is a music genre (54) that became known to the public through the spread of singer MTV (55). Street dancers use New Jack Swing music to dance, creating many classic steps. Kojima et al. (47) defined New Jack Swing as “…a music genre that became popular in the late 1980s, and these steps are called New Jack Swing because they were often used to dance to this type of music” (p. 3). Not only that, singers and dancers performing dances in music video created many classic steps through New Jack Swing, such as Bobby Brown (56) Therefore, New Jack Swing is a street dance style that emerged in the late 1980s, popularized through music videos by artists such as Bobby Brown. It has become a significant part of popular music culture and hip-hop dance, offering an expressive and dynamic movement vocabulary tied to the rhythm and beats of New Jack Swing music.

New style is often thought of as a hybrid style that blends different hip-hop dance styles together (57).A representative dance group of the New style is Elite Force, who mixed steps from New Jack Swing with elements from hip-hop dance to create the new style and released “their whole DVD thing and their whole old school new school Hip-hop dictionary” (23). In fact, there are different understandings of hip-hop dance in the academic world. Some scholars believed that new style is a style that continues to innovate (36), and some scholars argued that new style refers to the transformative dance steps of hip-hop rap music such as “Happy Feet, the Roger Rabbit, the Biz Markie, the Running Man, and the Skate” (58). All of these ideas seem to point to a state of being, i.e., new style is the state of evolving hip-hop dance, which is not only a specific style of street dance that evolves with hip-hop music and movement styles, but also sometimes a hybrid style.

House, like New Jack Swing, is a musical genre where house dancing is created by house dancers (58). Sommer (59) argued that “house dance was developed in the clubs at the end of the disco era” (p. 134), house consists of many dance steps including “Lindy and bebop, African, Latin salsa, Brazilian capoeira, jazz, tap, and modern” (60). House did not originate from hip-hop culture because of the addition of hip-hop dancers to the scene, which gave house many dance steps. Thus, house as a club culture, was absorbed into hip-hop dance, which in turn became part of the street dance. In this view, I agree with Perillo (23) and Ojofeitimi et al. (37), house is a kind of street dance that developed in clubs at the end of the disco era.

Rose et al. (61) described krump as “.…a fast-paced, highly expressive form of dance, most often practiced as a free-form improvisation, characterized by sharp and clear movements” (p. 1). In Batiste (45), krump was produced in South Central Los Angeles. Krump became known to the general public and included in popular culture. In the current academic literature, krump and hip-hop dance are often conflated in the current academic literature (61). This may be due to some scholars' misunderstanding of the definition of hip-hop dance, which is, an umbrella term for popular dance in hip-hop culture (19). Krump has exclusive technical moves, such as “chest pops, arm swings, jabs, stomps, and a percussive focus” (61). The specialized technical movements establish a dance style unique to krump, and as a result, krump has been incorporated into the street dance system (5, 37, 62).

In addition to the aforementioned street dance styles, some non-mainstream hip-hop dances also need to be enumerated, and after careful selection, I have decided to present two non-mainstream hip-hop dances: Crip-walk (as know as C-walk) and lite feet, which although considered by some scholars to be minoritization dance style (51, 62–64), but these styles are widely used by dancers during choreography, batte, cypher.

Crip walk had gang connotations in its early formative years and was called Crip walk or C-walk (65, 66). Crip walk, although well known as a hip-hop dance, has a smaller audience, possibly due to the fact that “.…calculated insultto the Bloods” (65). Nowadays, the social scene has seen the crip walk subsumed within the broader category of street dance (64), becoming a hip-hop dance step characterized by footwork.

Lite feet, also known as Getting Light, Get Light, or Get Lite, the youngest dance style to emerge in Harlem at the beginning of the 21st century (67). Similar to the Crip walk, Lite feet is rooted in the hip hop tradition (68), also featuring footwork, and has been called “ the rebirth of hip hop through dance”. Dancers demonstrated their intimate knowledge of New York City by practicing on the streets and subways, performing gymnastic movements on the floors and poles of moving subway cars (69). Lite feet is the creation of a younger generation of dancers, a style of dance that breaks away from the early hip-hop dance gang colors and used positive emotions to help young dancers innovate street dance.

### 5.3 What are the factors that contribute to the public knowledge gap?

**Figure 6:**
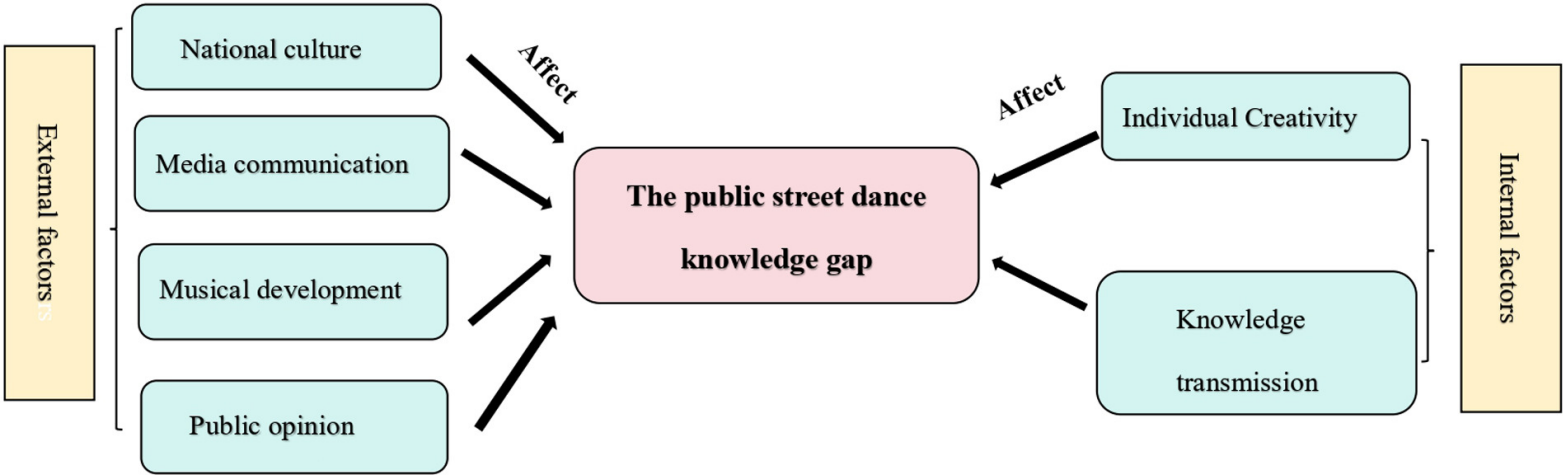
A framework of factors influencing the public street dance knowledge gap. The factors influencing the public street dance knowledge gap are categorized into external and internal factors in the figure, and the refinement of the influencing factors is carried out.

I analyzed to determine the internal and external influences on street dance knowledge gap. Internal factors including individual creativity, knowledge transmission. External factors including national cultural, media communication, music development, public opinion. The details are shown in Figure 6.

#### 5.3.1 Internal factors

##### 5.3.1.1 Individual creativity

Individual creativity refer that street dancers innovate moves and adapt styles to personal identity and emotions (9). In the background of the widespread spread of hip-hop culture, a number of style founders have been born. For example, Don Campbell of West Coast created the Campbell Lock, Boogaloo Sam created the Boogaloo style (26, 70). Interestingly, Campbell Lock has since been given the new name “Lock dance” (25), and the Boogaloo style has been included in the theoretical framework of popping dance (16), such results lead to different understandings of the superimposition of terms. In addition, some styles that lack a recognized founder can also create knowledge gaps, most typically street jazz, a classic style of street dance where the founder is not yet clear (37, 71), also led to varying interpretations of the public's understanding of street dance terminology, e.g., another alias for street jazz is new jazz dance (52). As a result, founders of different styles of street dance have coined different terms, and those styles that lack founders' definitions create a knowledge gap.

##### 5.3.1.2 Knowledge transmission

Knowledge transmission is carried out through crews foster innovation (15, 72). For example, in Sun in Li (73)'s study, “dance group (composed of Henry Link, Loose Joint, Buddha Stretch) developed a new style of street dance” (p. 1216). Not only that, but Locking was also initially known as Campbell lock, with dance movements created by team members (25). This iteration and development of new styles through the succession of founders and disciples or teammates, musical styles, and technological advances has created new names for street dance styles. With the emergence of a new generation of dancers on stage, new street dance terminology is spread. New and old street dance dancers have different understandings of old and new street dance terminology, resulting in the creation of a street dance knowledge gap.

#### 5.3.2 External factors

##### 5.3.2.1 National culture

National culture refers to a set of values and beliefs developed by a group of people over time in relation to their environment, a “way of seeing the world” (74). Street dance styles did not start out so rich, popular dance cultures from a variety of national culture influenced and fused with each other, eventually forming the present-day stylistic framework (26, 67). As Pond (42) described, “In short succession, Robot influenced dances, such as the Campbellock (or Locking), Waving, Popping, and the Electric Boogaloo emerged on the West Coast and, by mid-decade, Breakdancing on the East” (p. 130). Regional differences (e.g., New York's Breaking vs. California's Popping) shape stylistic preferences (5), and the fusion of different styles of popular dance forms the framework of street dance, yet each retains its original name. the original name of the dance. However, as street dance continued to spread to different countries, different names arose, resulting in the emergence of regional terms such as “Jiewu”, etc. (19). This kind of special appellation is an alias for street dance by dancers in different cultures, and the alias of different regions is one of the important reasons for the knowledge gap in street dance. I have compiled explanations of some of the common hip-hop terms so that readers can better understand them, as detailed in Supplementary Material D.

##### 5.3.2.2 Media communication

Media campaigns have prompted a variety of titles for street dance (22, 47), for example, the movie “Breakin’” aired in the United States in 1984, breaking, locking and popping were collectively referred to as breakdancing or breakdance (5). Interestingly, in China, breaking, locking and popping are referred to as “PILIWU” (3, 19). “PILIWU” as a name for commercial promotion is actually a Chineseized alias for breakdance (15). Media communication has designed different street dance promotional terms, causing confusion in the public's understanding of street dance and creating a knowledge gap.

##### 5.3.2.3 Musical development

The evolution of street dance music can be traced back to the 1970s, when hip-hop music mixed funk, soul, disco, and salsa music (33), extending a variety of styles of street dance music, such as New Jack Swing and house (47, 59). The development of street dance music has spawned new styles of street dance and influenced the public perception of street dance, leading to the emergence of different street dance terminology in different regions (73). For example, funk music is commonly used for popping, locking, and waacking (50), house music is specialized for house dance (59), and hip-hop music is suitable for all street dance styles (75). Whether it is funk, house, or any other style of music, it has been blended into hip-hop music and has confused the public's perception of the music, thus indirectly affecting the understanding of street dance.

##### 5.3.2.4 Public opinion

Public opinion is an important factor influencing the perception of street dance. Speier (76) argued that “opinion disclosed to others or at least noted by others, so that opinions which are hidden or concealed from other persons may be called either private or clandestine opinions” (p. 376). In 2024, Breaking, as an Olympic sport (5, 15), gained widespread public recognition. International Olympic Committee have noted that “Breakdancing, as breaking is popularly known” (34). This terminology can lead the public to conflate breaking and breakdancing, and may even result in the misconception that all breakdance styles are included in the Olympic program (15).

## 6 Conclusion and limitation

This review redefined the concept of street dance to help street dance researchers and learners bridge the street dance knowledge gap. Initially, I developed three templates of definitions of street dance that could be used as a reference. Subsequently, I devised a theoretical framework for street dance that encompasses stylistic divisions between old school and new school periods. In this section, I merged hip-hop dance and other styles under the overarching framework of street dance so that readers would have a clear understanding of the street dance theoretical framework. I suggested that street dance theoretical framework can be applied by researchers to the study of street dance terminology. This street dance theoretical framework not only clarifies the temporal and stylistic evolution of street dance but also provided a foundation for standardizing street dance terminology and guiding future research.

Moreover, this review explored the internal and external factors influencing the street dance knowledge gap, including individual creativity, knowledge transmission, national cultural, media communication, musical development, and public opinion. By examining the causal relationships between these factors, I demonstrated how discrepancies in defining street dance have contributed to confusion among scholars, practitioners, and the general public. This review underscored the importance of a unified definition and framework to promote consistency in academic research, street dance education, and cultural representation.

Several limitations should be acknowledged. First, the review was limited to peer-reviewed literature written in English, potentially excluding valuable insights from non-English sources and oral histories. Second, the complexity of street dance terminology and its evolution across different cultural contexts may have led to the omission of certain nuanced interpretations. Third, while the proposed framework offers a comprehensive categorization, the dynamic and evolving nature of street dance means that new styles and terms may emerge, requiring future adjustments to the framework. In addition, the review relied primarily on secondary sources, which may introduce biases or gaps in the data. Future research should incorporate ethnographic fieldwork and direct engagement with street dance practitioners to enhance the validity and applicability of the framework.

Finally, I hope that this review will facilitate a dialog between street dance scholars of different styles in the field of street dance. To enhance the dissemination of ideas, a unified theoretical framework is necessary to mitigate the impact of the knowledge gap on research credibility in street dance academic field. While I can adopt other scholars' perspectives to corroborate street dance, I must avoid being influenced by these definitions because it is not clear that there are theoretical references to these perspectives. Similarly, I can bridge the knowledge gap in the public's perception of street dance by using a unified theoretical framework while celebrating the complex differences between different styles of street dance. In order to accomplish this goal, I will continue to refine and expand the theoretical framework of street dance.

## Data Availability

The original contributions presented in the study are included in the article/Supplementary Material, further inquiries can be directed to the corresponding author.
