# Supplementary material for: Street dance redefined: a bridge across the knowledge gap
Source: Front Sports Act Living. 2025 Jul 23;7:1610656. doi: 10.3389/fspor.2025.1610656 (PMC12325215; doi:10.3389/fspor.2025.1610656)
Supplement: Supplementary file 2 [file Supplementaryfile2.docx]

**Appendix B: Supplementary Material – Raw Data Set**

**[Sample** Number: 1**]**

**[Title:** Hip Hop Dance Experience Linked to Sociocognitive Ability] **[Source:** https://doi.org/10.1371/journal.pone.0169947**]
[Edited? No]**[**Reason for Inclusion:** This article explores how hip-hop dance experience is linked to cognitive and social cognition skills, particularly mental rotation, problem-solving, and emotion recognition. It is relevant for understanding how street dance, including hip-hop, influences sociocognitive abilities, contributing to discussions on street dance pedagogy and its broader educational and psychological benefits.]
[**Coding Date:** 02/18/2025]
[**Coder:** First author]

[**Main Points / Impressions:**

This study examines the impact of hip-hop dance experience on sociocognitive skills, drawing parallels between kinesthetic expertise and cognitive abilities found in other domains like sports and gaming. The research demonstrates that hip-hop dancers exhibit enhanced mental rotation skills and a trend toward better emotion recognition, indicating that hip-hop dance fosters cognitive and social abilities relevant to STEM education and social interaction.The study is particularly valuable for research on street dance and education, as it positions hip-hop as a tool for cognitive and social development, challenging traditional academic biases against kinesthetic learning.]

**Writing Purpose Statements**

The article aims to investigate whether hip-hop dance enhances specific sociocognitive skills and draws comparisons with kinesthetic and gaming expertise research. It specifically assesses working memory, mental rotation, problem-solving, and theory of mind.

Example from text:
"To determine whether hip hop dance experience may be connected to mental processes underlying academic performance, we examined whether accumulated experience in this activity was linked with individual differences in working memory, mental rotation, problem solving, and social cognition skills.In line with previous research, these skills may be connected to hip hop dance experience for the following reasons. With regard to working memory, when learning or developing choreography for a routine, hip hop dancers have to notonly coordinate multisensory information (e.g., music, kinesthetic, visual) but also have to maintain their spatial position on a dance floor according to a predetermined sequence of movements." (p.5).

**Methodology and Data Collection (Provide Evidence to Support the Credibility of the Study’s Findings)**

This study employs a quantitative experimental design, using computerized tasks to assess cognitive and social abilities among hip-hop dancers and non-dancers. It relies on reaction time-based performance tests and survey data to quantify dance experience.

Example from text:
"We recruited dancers who varied in levels of experience across different dance styles. We defined dance experience as the number of estimated hours that individuals had spent either deliberately practicing or teaching choreography to other dancers" (p.5).

**Data Collection Methods:**

(1)Survey: Participants estimated hours of dance practice and teaching experience.

Example from text:
“The total number of hours of dance experience significantly correlated with self-reported ratings of dance experience (hip hop: r[59] = 0.783, p < 0.001; other styles: r[59] = 0.732, p < 0.001) providing evidence that total number of hours was a valid measure of dance experience.”(p.12)

(2)Cognitive Tasks: Participants completed mental rotation tests, problem-solving tasks, and emotion recognition tasks using computerized decision tasks.

Example from text:
"To assess sociocognitive skills, we utilized computerized decision tasks, specifically the symmetry operation span task, block and hand mental rotation tasks, the Tower of London task to assess problem-solving ability, and the Reading the Mind in the Eyes (RME) task to assess emotion recognition skill" (p.6).

**Provide Evidence to Support the Transferability of the Study’s Findings**

The study suggests that hip-hop dance may enhance sociocognitive abilities across different age groups and dance backgrounds. The findings are transferable to other kinesthetic fields such as sports and gaming, where similar cognitive benefits have been observed.

Example from text:
"We suggest that these results indicate that hip hop dance experience is connected to specific sociocognitive skills. We discuss ways in which future research can build upon the present study, such as examining the causal direction of the observed links between hip hop dance experience and sociocognitive skills, whether these connections are observed with dancers in different geographic regions and age groups, and if hip hop dance training could be used to supplement STEM education." (p.21).

**Provide Evidence to Support the Dependability of the Study’s Findings**

The study controls for demographic factors (e.g., age, gender) and uses linear mixed models to ensure robustness in its statistical analysis. Furthermore, various cognitive measures were tested to strengthen the reliability of findings.

Example from text:
"We used linear mixed models to analyze performance as these types of models are able to account for variation in performance across participants and stimuli and are relatively robust to unbalanced data" (p.6).

**Provide Evidence to Support the Confirmability of the Study’s Findings**

The study acknowledges its limitations and suggests future research directions, emphasizing the need for further investigations into causal relationships between hip-hop dance and cognitive ability.

Example from text:
"Future research using training paradigms where participants are given experience with hip hop dance can help elucidate the causal direction of the link between hip hop dance experience and select sociocognitive skills" (p.20).

Additionally, the authors highlight that regional and stylistic variations in hip-hop dance may influence results, calling for broader studies across different cultural contexts.

Example from text:
"Given the variability in the styles of hip hop dance, it remains to be seen whether connections between dance experience and mental rotation ability vary across geographic regions" (p.20).

**Conclusion**

This article provides compelling evidence that hip-hop dance enhances sociocognitive skills, particularly in mental rotation ability and emotion recognition. While the findings align with research on kinesthetic learning, they also highlight hip-hop dance as a cognitive and social development tool, with implications for STEM education, youth programs, and urban dance pedagogy.

**References (Use a Specific Style Referencing)**

For example,

OECD. Lessons from PISA 2012 for the United States. Strong Performers and Successful Reformers inEducation. OECD Publishing; 2013.

This reference, which is not labeled with its accessible address, after I looked it up, this article is available at https://www.oecd.org/content/dam/oecd/en/publications/reports/2013/12/lessons-from-pisa- 2012-for-the-united-states_g1g397ea/9789264207585-en.pdf. Unfortunately, the author did not add it.

[**Sample Number:**2]

[**Title:**Experiences of Youth Mentoring Through Street Dance ]

[**Source:**https://doi.org/10.1177/1473225419879248 ]

[**Edited?** No]

[**Reason for Inclusion:**This study evaluates a mentoring program for at-risk youth in the UK, integrating street dance as a key intervention strategy. It explores the psychological and social benefits of mentoring combined with street dance, particularly in fostering positive relationships, improving emotional regulation, and opening life opportunities for the youth involved.]

[**Coding Date:** 02/18/2025**]**

[**Coder:** First author]

[**Main points/impressions:**

Note. *The reference style of this dataset will be evaluated based on SAGE Harvard (https://journals.sagepub.com/author-instructions/IRS#ReferenceStyle)*

This study examines how street dance mentoring supports at-risk youth by addressing their emotional challenges, improving self-confidence, and providing a framework for positive future outlooks. The research highlights the mentor-mentee relationship, emphasizing the importance of a non-hierarchical and trust-based approach. Additionally, the role of street dance in promoting social bonding, self-expression, and coping with negative emotions emerged as a crucial factor in facilitating mental well-being and desistance from antisocial behavior. The study calls for further evaluation of dance-based interventions for youth at risk of offending.]

**Writing Purpose Statements**

The study explores the role of street dance as a mentoring tool for youth, emphasizing its impact on personal development and community building. The research aims to understand how participation in street dance programs fosters positive mentorship relationships.

Evidence: The article states, “This study presents a qualitative evaluation of a community mentoring programme in which the key component is participation in street dance.”(p.3)

**Methodology and Data Collection – Provide Evidence to Support the Credibility of the Study’s Findings**

The study follows a qualitative research design, collected data through semi-structured interviews. It incorporates multiple data sources but lacks evidence of longitudinal data collection.

Evidence: "The participants were recruited from a Youth Centre in a provincial UK city. The centre is non-profit and run by a charity which provides free and confidential information and support to young people aged 13–25. Service users can be referred to the centre by support workers or school, or attend of their own volition – it is situated in a busy area and invites drop-ins from young people in need of support at any time. The locality has several nearby areas classed some of the most deprived in the United Kingdom" (p.4).

The data sources included two different groups of people, for example,“The

participants were a male mentor from the centre and eight service users, aged 16–18 who had completed the 2-year mentoring through street dance scheme within the previous 6months. All had either offended or were considered at risk of doing so. The latter were identified as at risk by their school or family due to their behaviour and/or because they were mixing with known offenders. Fifteen young people (12 males, 3 females) had completed the scheme within the previous 6months, were still using the centre and therefore still known to the mentor on an informal basis”(p.4).

**Provide Evidence to Support the Transferability of the Study’s Findings**

The findings offer a thick description of mentorship practices in street dance, making them transferable to similar community arts programs. However, the study does not explicitly explain its sampling strategy or compare findings to other contexts.

Evidence:"In conclusion, this is one of the very few UK studies of mentoring which has considered a dance-focussed programme and the first in terms of street dance."（p.14）

**Provide Evidence to Support the Dependability of the Study’s Findings**

The study fully follow the principles of data saturation.

Evidence:

"Eight participants (aged 16–18) and their mentor took part in semistructured interviews which were transcribed verbatim and the data subjected to thematic analysis."(p.1)

"A thematic analysis of the transcriptions was conducted according to the guidelines offered by Braun and Clarke (2006). A master table of themes was developed by an iterative process of reading and rereading each transcript, noting convergences and divergences in the data throughout. Both authors considered the data to ensure veracity of themes (p.6). "

**Provide Evidence to Support the Confirmability of the Study’s Findings**

The study lacks explicit peer debriefing or audit trails, nor does it articulate the limitations of the study, or search the data/literature for evidence inconsistent with the study findings.

Only some of the limitations of the study are noted in the text, for example, "However, before street dance-based interventions can be rolled out more

widely, further evaluations will be required to test the veracity of our findings in a

variety of locations. These studies should also focus on explicating the mechanisms which make such programmes effective. Our first recommendation therefore

is for further evaluation studies. "(p.14)

**Use a Specific Style Referencing**

The reference list for this paper contains some, but not all, of the elements that meet specific formatting requirements (e.g., APA, MLA, Chicago style), such as the full title, the names of all authors (last name + initials), the name of the journal, the year of publication, the volume number, and the page number. For example, some documents are missing DOI or access addresses.

The references include:

Bouffard JA and Bergseth KJ (2008) The impact of reentry services on juvenile offenders’ recidivism. Youth Violence and Juvenile Justice 6: 295–318.

Braun V and Clarke V (2006) Using thematic analysis in psychology. Qualitative Research in Psychology 3: 77–101.

Cain KL, Gavand KA, Conway TL, Peck E, Bracy NL, Bonilla E, Rincon P and Sallis JF (2015) Physical activity in youth dance classes. Paediatrics 135: 1066–1073.

Warburton EC, Wilson M, Lynch M and Cuykendall S (2013) The cognitive benefits of movement reduction: Evidence from dance marking. Psychological Science 24: 1732–1739.

Sullivan CJ and Jolliffe D (2012) Peer influence, mentoring and the prevention of crime. The influence of mentoring on reoffending. In: Welsh BC and Farringdon D (eds) The Oxford Handbook of Crime Prevention. Oxford: Oxford University Press, 207–225.

Steinberg L, Graham S, O’Brien L, Woolard J, Cauffman E and Banich M (2009) Age differences in future orientation and delay discounting. Child Development 80: 28–44.

Stevens L (2006) Urban youth street dances go to the movies and become ‘breakdancing’. In: Cann KR (ed.) Dance/Diversity/Dialogue: Bridging Communities and Cultures. Proceedings of the World Dance Alliance Global Assembly. Toronto, ON, Canada: World Dance Alliance, 361–369.

**(...)**

[**Sample Number:** 3]

[**Title:** Shaping Future Directions for Breakdance Teaching]

[**Source:** https://doi.org/10.3389/fpsyg.2022.952124]

[**Edited?** No]

[**Reason for Inclusion:**This article discussed how breakdance teaching can evolve in the coming years, addressing both teaching methodologies and the influence of cultural shifts. The article describes the historical relationship between breakdancing and street dance and is an important reference for the reconstruction of the definition of street dance.In addition, this article's study of search terms also contributes to the reconstruction of the concept of street dance, for example, when searching for the term “breakdance”, in order to ensure that as much relevant literature as possible is searched for, the term “street dance” may be used as a complementary. It can be seen that the authors have a deeper understanding of the formation and definitional reconstruction of street dance.]

[**Coding Date:** 02/18/2025**]**

[**Coder:** First author]

[**Main points/impressions:**

Note. *The reference style of this dataset will be evaluated based on SAGE Harvard (https://journals.sagepub.com/author-instructions/IRS#ReferenceStyle)*

This supplementary material delves into the future directions for breakdance education, with a focus on how teachers can adapt their teaching methods to bridge the gap between hip-hop culture and formal dance education. The study discusses how street dance culture continues to influence the pedagogical approaches of breakdance instructors, highlighting the importance of creativity, cultural authenticity, and community engagement in teaching practices.Notably, creativity, cultural authenticity, and community engagement somewhat influenced the formation of the street dance definition and its categorization.**]**

**Writing Purpose Statements**

This article aims to enhance the quality of breakdance teaching and learning by identifying the positive impact of formative assessment on student learning and designing a formal formative assessment task related to breakdance. The authors argue that while breakdance has gained significant attention due to its inclusion in the 2024 Olympic Games, there is a lack of research on formal formative assessments in breakdance education. The study seeks to address this gap by providing a framework for assessing breakdance students' abilities in formal educational settings. For example, the text reads: “Given the inclusion of this dance form in the 2024 Olympic Games in Paris, France, scholars subsequently generated a substantial body of international research related to breakdance teaching... However, few researchers have focused on the impact of formal formative assessment on breakdance teachers’ teaching and students’ learning.” (Wei et al., 2022, p. 2).

**Methodology and Data collection (Provide Evidence to Support the Credibility of the Study’s Findings)**

This aritcle utilized a qualitative research design with existing breakdance research literature as the primary source of data. The researchers collected breakdance assessment studies and designs of educational methods and teaching styles from different countries, synthesized different assessment methods of breakdance to innovate, and summarized a set of programs suitable for the assessment of breakdance students.

**Methods of data collection:** review and screening of breakdance literature, such as studies of classroom observations of breakdance lessons and practice sessions(Foley, 2016); and studies of the design of breakdance textbooks and lesson plans.Although this paper is based on literature to collect data, the literature itself contains different research methods, for example, “This section will focus specifically on the breakdancing assessment items, criteria, and preparation for students before the assessment” (p. 3).

The authors conducted a comprehensive review of peer-reviewed articles from the EBSCO and Web of Science databases, focusing on studies that included formative assessment task design relevant to breakdance. The inclusion criteria were: (1) written in English, (2) peer-reviewed articles, (3) related to breakdance, and (4) articles that included items or criteria related to assessment skills. For example, the text reads: “This article has collected formative assessment tasks related to breakdance from the EBSCO and Web of Science databases... The following inclusion criteria included (1) written in English, (2) peer-reviewed articles, (3) related to breakdance, and (4) articles (or supplemental materials) must include items or criteria related to assessment skills” (p. 3).

“Again, space limitations make an extended discussion of these findings impractical. Interested readers are therefore referred to other relevant sources (Foley, 2016; Vasil, 2020; Sato and Hopper, 2021)....Vasil's assessment task was a low-stakes and formative assessment (Vasil, 2020). The results of the assessment were used in a proposal for bringing pop culture into the arts-integrated curriculum. conducted at the end of the course, and students will perform a breakdance show. Through three music lessons and three art lessons, students avoid, to some extent, unexpected reactions to the assessment items. Through three music lessons and three art lessons, students avoid, to some extent, unexpected reactions to the assessment items (Messick, 1995). For example, students will not be confused about the assessment items” (p.3). Therefore, the data in this paper were collected from multiple data sources.

**Research design**

This article presents three iterations of the research design, providing evidence of iterative data collection, iterative data analysis, and flexible/dynamic research design. For example, “According to the rules of rubrics proposed by Wolf and Stevens (2007) and Brookhart (2018), the assessment rubric will be reviewed in three iterations.- First iteration: Ensures that rubrics reflect the quality of the breakdancing student's cognitive and First iteration: Ensures that rubrics reflect the quality of the breakdancing student's cognitive and movement learning, with indicators ordered in terms of increasing proficiency, and using clear and easily understood language avoids ambiguous language. Second iteration: Be written so that students can verify their own performance and ensure that defined by a set of quality indicators that display a continuous level of growth, the breakdancing student's cognitive and movement learning is not a “one-stop shop”. Second iteration: Be written so that students can verify their own performance and ensure that defined by a set of quality indicators that display a continuous level of growth. Third iteration: Ensure that the criteria match the construct map to help breakdancing teachers identify student position in the construct map (see Figure 1)” (p.5).

**Provide Evidence to Support the Transferability of the Study’s Findings**

The study’s findings are supported by the detailed design of a formal formative assessment task that can be used to assess breakdance students' abilities. The assessment task is suitable for students aged eight and up and focuses on four key capabilities: analyzing dance movement combinations, executing breakdance movements, outlining breakdance history, and identifying breakdance terms. For example, the text reads: “This section will design a formal formative assessment task related to breakdancing. This is a low-stakes formative assessment task. The assessment is suitable for breakdancing students age eight and up. Breakdancing teachers can use it to assess their students in the following four capabilities...” (Wei et al., 2022, p. 4).

**Tables and Figures**

Table 1: Judgment-based assessment rubric.

Figure 1: Student Capability Progress Level Map.

**Provide Evidence to Support the Confirmability of the Study’s Findings**

In the text, the author reflects on the paper. For example, “This task is only tentative. we hope that this task will open more research and discussion on formal formative assessment items related to breakdance among scholars in different disciplines."(p.6). In addition, several co-authors reviewed the article written by the first author.

**Provide Evidence to Support the Dependability of the Study’s Findings**

The article presents a structured approach to formative assessment, including the use of assessment items, constructing maps, and judgment-based rubrics. The authors also discuss potential constraints and provide recommendations for minimizing construct-irrelevant variance. For example, the text reads: “Some potential constraints on the use of this assessment include... Recommendations for minimizing construct-irrelevant variance include the following...” (p. 4).

**Provide Evidence to Support the Confirmability of the Study’s Findings**

The authors reflect on the limitations of their study and suggest future research directions. They emphasize the need for further research to refine and validate the assessment tasks. For example, the text reads: “Although this is a low-stakes formative assessment task, this process will help design a high-stakes formative assessment task in the future... We hope that this task will open more research and discussion on formal formative assessment items related to breakdance among scholars in different disciplines.” ( p. 5).

**Conclusion**

The article concludes that breakdance teaching must balance technical proficiency with cultural authenticity. The authors argue that formative assessment can play a crucial role in improving teaching strategies and student learning outcomes. They suggest that future research should focus on developing more comprehensive assessment tools and exploring the impact of these tools on student performance and engagement. For example, the text reads: “This article describes the current state of breakdancing... Finally, we believe that if this article is to be truly helpful to breakdance teachers who are facing teaching dilemmas, then we need to try to design a formative assessment task.” (p. 6).

**References (Use a Specific Style Referencing)**

Bode Bakker, M., and Nuijten, M. (2018). ‘When breaking you  make your soul dance’ utopian aspirations and subjective transformation in breakdance. Identities 25, 210–227. doi: 10.1080/1070289X.2017.1400275

Chang, W., and Lee, J. E. (2022). The return of the prodigal B-boy: from marginalised subculture to protected Korean cultural institution. Cultural Trends, 1–15. doi: 10.1080/09548963.2022.2070724

CHUC (2020). ‘China Dancers Association street dance Committee Promotional

film’ [in Chinese] [video], CHUC Publications. Available at: https://www.chhuc.org/14164.html (Accessed June 29, 2020).

Foley, C. G. (2016). From b-girl to B-baby: constructing a breakin’ pedagogy. J. Dance Educ. 16, 62–66. doi: 10.1080/15290824.2015.1081206

Li, R. Z., and Vexler, Y. A. (2019). Breaking for gold: Another crossroads in

the divergent history of this dance. Int. J. History Sport 36, 430–448. doi:

10.1080/09523367.2019.1615896

Osumare, H. (2002). Global breakdancing and the intercultural body. Dance Res. J. 34, 30–45. doi: 10.2307/1478458

Shapiro, R. (2004). The aesthetics of institutionalization: breakdancing in France. J. Arts Manag. Law Soc. 33, 316–335. doi:10.3200/JAML.33.4.316-335

**(...)**

[**Sample Number:**4]

[**Title:**Breaking for Gold: Another Crossroads in the Divergent History of this Dance ]

[**Source:**https://doi.org/10.1080/09523367.2019.1615896 ]

[**Edited?** No]

[**Reason for Inclusion:**This article critically examines the evolving relationship between breaking (also known as breakdancing) and its recent inclusion as a competitive event in the Olympic Games. The article explores the historical development of breaking, its cultural significance, and the ongoing debate within the breaking community regarding its transition into a competitive sport. It emphasizes the tension between the dance’s roots in hip-hop culture and its commercialization and institutionalization.It is worth noting that although some of the views in this article differ from my own, I do not deny that this article makes a significant contribution to the reconstruction of the definition of street dance. This is a reference paper that tells about the terms related to street dance and has high academic value.]

[**Coding Date:** 02/19/2025**]**

[**Coder:** First author]

[**Main points/impressions:**

Note. *The reference style of this dataset will be evaluated based on SAGE Harvard (https://journals.sagepub.com/author-instructions/IRS#ReferenceStyle)*

This article provides an insightful look into the history of breaking, tracing its roots back to the hip-hop culture of the 1970s and examining how it has evolved over time. The authors discuss the cultural divergence between breaking as an art form and its growing recognition as a competitive sport, particularly with its inclusion in the Olympic Games. The article underscores the importance of cypher culture—where dancers perform in a communal, non-judged environment—as central to the breaking community’s identity, contrasting it with the more standardized, competitive structures that have emerged in global events. The article also offers a critique of the World DanceSport Federation (WDSF)’s involvement in Olympic breakdancing, emphasizing the challenges of maintaining the artistic essence of breaking in a commercialized, competitive context.]

**Writing Purpose Statements**

Evidence:“Dedicated to the historical evolution of breaking itself, the authors do not discuss how its popularity influenced the histories of performing arts and physical education, but rather focus on the dance and the community of dancers from which this influential popularityoriginates.”(p.431)

“Numerous events set the stage for its formation, but the birth of hiphop can be traced back to parties organized by DJ Kool Herc in 1973. Breaking was similarly crystallized from many sources of inspiration, but its birth can also be traced back to these same parties.”(p.431)

**Methodology and Data Collection – Provide Evidence to Support the Credibility of the Study’s Findings**

The authors conducted a longitudinal ethnographic study, drawing on their personal experiences within the breaking community. The research also includes historical analysis of key events in breaking’s development, such as the first international competitions and the growing influence of the media. Additionally, the authors analyzed Olympic proposals and public responses to the inclusion of breaking in global sporting events.

Evidence:“When asked about the breaking dancer population demographics during the 1970s,most first generation B-Boys and B-Girls answer that breaking was not a dance of any one specific ethnic group. It started in the poorer areas of the Bronx, but there were B-Boys and B-girls in Brooklyn, Queens, Harlem, Manhattan, and in places even further away, as early as 1975. The older generations say that they did not wish to view humankind as demographically separate, and the younger generations strive to continue this philosophy.”(p.432)

“Most B-Boys and B-Girls opposed to this dance becoming an Olympic event

believe that the cultural and social value of breaking is more precious than any

material profit that can potentially be gained by developing it as a sport. Adamant

that it should be promoted as an art form, they dislike any changes that may cause

their culture and their dance to lose its original essence. Others dislike the idea of

their dance being exploited (again) by people just trying to use its popularity to push

their own agendas, dislike the confusion spread by IOC documents, and also dislike

the emphasis on competitions. Introduced as a competitive sport, people will be

motivated to practice breaking only to compete in such events, perhaps not even

knowing about (or caring to participate in) the cypher. If cypher culture is forsaken

as this dance develops, the unique fundamental that made it so popular in the first

place would be lost.”(p.442)

**Data Collection Methods:**Ethnographic fieldwork in hip-hop and breaking communities worldwide.

Interviews with key figures in breaking history.

Historical document analysis of Olympic discussions and media representations of breakdancing.

**Provide Evidence to Support the Transferability of the Study’s Findings**

The authors provide a detailed description of the study's findings but do not explain the sampling strategy or do not discuss the resonance of the study's findings with the existing literature in different contexts.

Evidence:“Analysis of breaking history reveals the effects of outside forces: spectators of early dance battles were so used to competitive sports, yet so unfamiliar with hiphopideals, that they demanded to celebrate a winner; the media always found it easier toshare breaking in the context of a sport rather than try to explain its art or itsculture; most companies only sponsored breaking events if they resembled sporting

events, and this evolution cumulated with the recent plans of the WDSF and the

IOC... The Olympics welcome ‘breakdance’ because they wish to

keep up with the changing times, so if all involved remain committed to this goal, its

inclusion could possibly change the nature of the Olympics more than the Olympics

change the nature of breaking. Regardless, if they wish to have a larger influence on

how their own dance develops, B-Boys and B-Girls around the world just need to

become more united in material and in spirit to carry out more numerous and more

significant activities of their own design.(p.446)

**Provide Evidence to Support the Dependability of the Study’s Findings**

Evidence used e.g. statements made by dancers on social media platforms. This non-academic viewpoint used to prove the author's point of view seems to lack some authority.

Evidence:


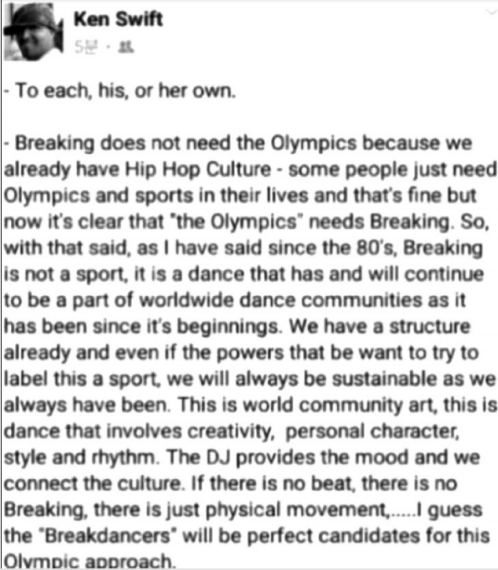


Nonetheless, the authors do cite official information as corroboration of their views, as follows:“Although the WDSF has never organized a breaking competition, the IOC finally agreed to let them try an Olympic event when they proposed ‘breakdance’.When applying for the chance to organize an Olympic breakdance event, the WDSF proposed that:

The inclusion of DanceSport/Breakdance into the programme will strongly support the IOC’s desire to attract youth, promote gender equality and increase the number of mixed-team events. Breakdance competitions are easy to organise and require minimal equipment, therefore it will not generate many additional expenditures. DanceSport also strongly aligns with the Olympic values of Friendship, Respect and Excellence.

They continued to explain:

Breakdance has a wide audience reach among Youth. Since no new infrastructure is

required, DanceSport contributes to efforts related to sustainability [and] contributes to the range of innovative ideas of the YOG to engage the youth in sport. [It] Offers

opportunities to join/participate and create a young, vibrant, innovative and festive”(p.441)

**Provide Evidence to Support the Confirmability of the Study’s Findings**

Ultimately, the author simply states the topic of whether breakdancing will be an art or a sport once it is in the Olympics, with no clear definition of the end result. For example, at the end of the article, it is written, “if they wish to have a larger influence onhow their own dance develops, B-Boys and B-Girls around the world just need tobecome more united in material and in spirit to carry out more numerous and moresignificant activities of their own design.”(p.446) This lack of a clear recommendation would make it appear that the article did not clear conclusion. In addition to this, the study lacks a clear peer debriefing or audit trail, nor does it articulate the limitations of the study, nor does it retrieve data/literature evidence that is inconsistent with the findings.

**Use a Specific Style Referencing**

Mary Fogarty, ‘Breaking Expectations: Imagined affinities in mediated youth cultures’,Continuum-Journal of Media & Cultural Studies 26 (2012): 449–62.

**(...)**

[**Sample Number:**5]

[**Title:**Develop a formative assessment protocol to examine the practice of Popping dance in students online learning ]

[**Source:**https://doi.org/10.3389/feduc.2023.1207086 ]

[**Edited?** No]

[**Reason for Inclusion:**This is an online study assessing students' learning of street dance, using one of the most classic styles of street dance: popping dance.There are 2 reasons for choosing this article: first, popping is one of the oldest styles of street dance, and it has a wide range of audiences all over the world, and because of the many styles of popping, there are also many different terms for it. First, popping is one of the oldest styles of street dance, and has a wide audience all over the world. Popping has many different names because of its many styles, the most famous of which is the Chinese “piliwu”, which can be better explained by this paper. Secondly, we searched for street dance styles on the web of science, among which, the literature of popping style was only searched to this one article, because this article has a representative meaning as the reference material of popping style.]

[**Coding Date:** 02/19/2025**]**

[**Coder:** First author]

[**Main points/impressions:**

Note. *The reference style of this dataset will be evaluated based on SAGE Harvard (https://journals.sagepub.com/author-instructions/IRS#ReferenceStyle)*

The article proposes a formative assessment model for Popping dance, focusing on assessing students' technical proficiency and theoretical understanding of the dance style. The assessment is designed for online environments, addressing the challenges of evaluating dance through remote learning platforms. The study emphasizes the use of digital tools like the Wukong App, integrating video recordings and interactive assignments to monitor students' dance performance, with a specific focus on assessing rhythmic synchronization, movement execution, and choreographic creativity.]

**Abstract**

This article develops an innovative formative assessment framework for the online learning of Popping dance, aiming to assess students' movement techniques, theoretical understanding, and rhythmic synchronization. It introduces a comprehensive assessment protocol utilizing video submissions, theoretical tests, and real-time feedback through dance-focused apps. The study outlines practical assessment tasks that evaluate various dimensions of dance proficiency, such as basic movements, execution quality, and choreographic integration. The assessment aims to guide both students and instructors in a remote learning context, helping students improve their dance techniques through continual feedback and personalized learning pathways.

**Writing Purpose Statements**

Evidence:“This article presents a diversified assessment model for the future evolution of Popping dance, informed by existing literature. It aims to furnish teachers with the tools necessary to guide students in performing App search videos, developing a knowledge system, demonstrating technical movements, and integrating dance choreography”(p.05)

**Methodology and Data Collection – Provide Evidence to Support the Credibility of the Study’s Findings**

The research team conducted an extensive review of existing literature on online dance assessments and developed a formative assessment protocol that is adaptable for Popping dance. The methodology includes the integration of video recordings for performance tasks and online quizzes for theoretical knowledge. Teachers were trained to use the Wukong App, a digital platform for dance learning, which facilitates video submissions for students’ performances. The data collection involved student performance analysis, self-assessment quizzes, and feedback mechanisms.

It is only in this paper that multiple data sources were used, containing different literature from different databases.

Evidence:

“In the development process of the formative assessment project, an exhaustive search was undertaken across various databases such as Web of Science, SAGE, and JSTOR to find articles pertinent to the formative assessment task related to students’ online learning of Popping dance. Literature was screened using the keyword “Popping

dance,” albeit the term was not consistently explicit. Some authors opted for related phrases like “Electric Boogie” (LaBoskey, 2001) or “Hip-hop dance” (Borges et  al., 2018). Importantly, only articles addressing both Popping dance and online dance assessments were included, with coverage spanning styles, classic characters, history,

and online education.”(p.02)

**Data Collection Methods(Assessment)**

Video-based assessment where students record and submit their performances.

Online theoretical testing using a variety of formats (e.g., multiple choice, short answer, fill-in-the-blank).

Real-time feedback via online platforms like the Wukong App, allowing students to track progress and receive targeted suggestions.

**Provide Evidence to Support the Transferability of the Study’s Findings**

The formative assessment designed in this paper has been found to be effective in other fields, such as those described in the text:“To address the potential environmental shortcomings in online teaching, it may be useful to think of formative assessment as a tool (Airasian, 1968; Li, 2020; Heyang and Martin, 2021). Effective use of formative assessment in this research is defined as an effective means of tracking student progress and identifying areas in need of improvement (Dean, 2007; Bennett, 2011; Antoniou and James, 2014;Andrade et  al., 2015; Spector et  al., 2016; Rasooli et  al., 2018)（p.02）”

**Provide Evidence to Support the Dependability of the Study’s Findings**

This article presents three iterations of the research design, providing evidence of iterative data collection, iterative data analysis, and flexible/dynamic research design. For example,“The first iteration is the establishment of assessment criteria, classified into five primary areas: mastery of the Wukong App for tutorial purposes, comprehension of Popping dance terminology, proficiency in executing basic Popping dance movements, ability to dance in rhythm, and competency in merging choreography with music. The second iteration sets the assessment level, comprised of five levels indicative of progressive growth. Students evaluate their test performance based on these levels and receive feedback to improve their skills. In the third iteration, assessment descriptions are crafted for each scoring item corresponding to the evaluation form. To ensure teachers can accurately score students, each standard is characterized by identifiable attributes. These iterative formative assessment tasks are designed to provide a straightforward and instinctive learning and assessment experience via the network. Furthermore, we have intentionally developed a variety of item types.” (p.04)

More iterative evidence is detailed:Tables and Figures

Table 1: Student capability progress level map.

Table 2:Assessment item

Figure 1: Judgement-based assessment rubric.

**Provide Evidence to Support the Confirmability of the Study’s Findings**

In the text, the author reflects on the article. For example, “The assessment model emphasizes software operation, theory testing, technical testing, among others, yet it acknowledges its limitations. These limitations may originate from environmental, human, and subjective factors, with the latter being the most challenging to control. While it is impractical to eliminate all irrelevant variances, assessors are urged to establish communication with participants beforehand, secure their trust, and foster a conducive test environment.”(p.05)

**Conclusion**

The study presents a versatile formative assessment protocol for evaluating Popping dance in online learning environments. By integrating video-based submissions, online theory testing, and real-time feedback mechanisms, the model supports the development of technical skills and theoretical understanding. The assessment allows for individualized feedback, helping instructors tailor their teaching methods to students' needs. The results underscore the effectiveness of online assessments in dance education, suggesting that such frameworks can improve student performance even in remote contexts, despite the challenges posed by online learning formats.

**References(Use a Specific Style Referencing)**

Wei, M. T., Yang, Z., Bai, Y. J., Yu, N., Wang, C. X., Wang, N., & Cui, Y. S. (2022). Sha** future directions for breakdance teaching. Frontiers in Psychology, 13, 952124.https://doi.org/10.3389/fpsyg.2022.952124

Yang, Z., Bai, Y., and Wei, M. (2022). The importance of creativity in the sportification of breakdance. Front. Educ. 7:855724. doi: 10.3389/feduc.2022.855724

Li, R. Z., and Vexler, Y. A. (2019). Breaking for gold: another crossroads in the divergent history of this dance. Int. J. Hist. Sport 36, 430–448. doi: 10.1080/09523367.2019.1615896

LaBoskey, S. (2001). Getting off: portrayals of masculinity in Hip hop dance in film. Dance Res. J. 33, 112–120. doi: 10.1080/0141192960220502

(...)

[**Sample Number:** 6]

[**Title**: Breaking Expectations: Imagined Affinities in Mediated Youth Cultures]
[**Source**:https://doi.org/ 10.1080/10304312.2012.665845]
[**Edited**? No]
[**Reason for Inclusion:** This article examines the mediated experiences of hip-hop and funk dance practitioners, particularly those involved in breaking (b-boying/b-girling). It explores the concept of ‘imagined affinities’ and how media, including videotapes, transnational networks, and commercial influences, have shaped the global spread of breaking. This study is valuable for understanding how street dance definitions and cultural exchanges evolve in a digital and globalized era.]
[**Coding Date:** 02/19/2025**]**

[**Coder:** First author]

[**Main Points / Impressions:**

This study introduces the concept of imagined affinities, which describes the mediated encounters between dancers across geographic and cultural boundaries. It argues that video artefacts, international dance events, and underground distribution networks have played a crucial role in shaping the way breaking is perceived and practiced globally.The article also historicizes the influence of media on breaking, highlighting how the proliferation of homemade videotapes in the 1990s allowed dancers to document, circulate, and shape breaking’s aesthetic values and codes beyond their local scenes. This process challenged dominant media narratives, which had previously misrepresented or commodified breaking.**]**

**Writing Purpose Statements**

The study investigates the role of media in shaping breaking culture and transnational dance identities, with a focus on the impact of videotapes, online representation, and global exchanges.

Example from text:

“This article examines the mediated encounters experienced by participants in hip hop

and funk dance styles especially breaking or b-boying/b-girling.”(p.449)
"I argue that artefacts made, distributed and circulated by dancers help to produce perceptions of commonalities between them. The nature of the process of rapid mediatisation, which has taken place during the past few decades, and its subsequent impact on breaking or b-boying/b-girling, are considered here through a concerted effort to historicize shifts in practice and experience" (p. 449).

**Methodology and Data Collection (Provide Evidence to Support the Credibility of the Study’s Findings)**

This study employs a mixed-method qualitative approach, combining:

(1)Interviews with dancers from New York, Los Angeles, Toronto, UK, and Germany

(2)Participant observation at dance events and communities

(3)Archival research of media materials, including underground video footage

(4)Multi-sited ethnography to analyze cross-cultural exchanges

Example from text:
"For this study, I use a mixed-method, qualitative approach including interviews, participant observation, archival research, multi-sited ethnography and video analysis. This combination of methods produces an overlap between interpretation and experience" (p. 451).

**Data Collection Methods:**

(1)Primary interviews with experienced and novice breakers

(2)Analysis of underground dance videos as cultural texts

(3)Ethnographic observations of dancers’ interactions in global events

Example from text:
"For some of the local dancers I interviewed later on, both home videos and videos bought from other dancers were significant factors in their understanding and articulation of knowledge about the dance" (p. 451-452).

**Provide Evidence to Support the Transferability of the Study’s Findings**

The study’s findings are transferable to other studies on cultural globalization and mediated youth cultures. It demonstrates that dance, like language, can be transmitted through visual media, enabling transnational learning and adaptation.

Example from text:
"The transnational spread of breaking is in many ways inseparable from the impact of video technology on the art form" (p. 450).

It also suggests that breaking, as a cultural form, follows patterns similar to other subcultures where media acts as both a unifying and differentiating force.

Example from text:
"In b-boy/b-girl culture, knowledge circulated through mediated and embodied elements such as video artefacts and travelling dancers" (p. 453).

**Provide Evidence to Support the Dependability of the Study’s Findings**

The study relies on multiple data sources (interviews, archival footage, ethnographic accounts), ensuring a robust triangulation of evidence. Additionally, it cross-references media representations with firsthand dancer testimonies, enhancing reliability.

Example from text:
"Ethnographic studies of breaking neglect a consideration of how the meaning of the dance is negotiated, shared and challenged transnationally through mediation. In this case, underground b-boy/b-girl video archives capture the conversations between dancers and demonstrate how participants have negotiated in cross-cultural exchanges" (p. 451).

**Provide Evidence to Support the Confirmability of the Study’s Findings**

The study reflects critically on how media representation can simultaneously empower and distort breaking culture, acknowledging both the benefits and limitations of mediated dance experiences.

Example from text:
"Breaking identities are not only about musical tastes and significations of dress and activity. They also are centred on the ‘show and prove’ mentality of belonging through the display of abilities in performance" (p. 460).

It also recognizes its own limitations, particularly the challenges of fully capturing the nuances of live, embodied dance experiences through media.

Example from text:
"Though the value judgements of the culture continue to centre on the live experience, face-to-face encounters are actually mediated by reputations gained through movies, videos, video games and internet websites" (p. 460).

**Conclusion**

This article challenges traditional theories of subculture and mediation by demonstrating how breaking evolved transnationally through media networks rather than direct physical contact alone. It highlights the importance of video artefacts, digital exchanges, and underground distribution channels in shaping how dancers perceive, learn, and engage with breaking culture globally.The study ultimately contributes to a broader understanding of how street dance definitions evolve through mediated interactions, emphasizing that breaking is not just a dance style but a cultural system shaped by history, technology, and social exchange.

**References (Use a Specific Style Referencing)**

Haraway, Donna. 1988. Situated knowledges: The science question in feminism and the privilege of partial perspective. Feminist Studies 14: 3 (Autumn) 575–99.

Schloss, Joseph G. 2006. “Like old folk songs handed down from generation to generation”: History,canon, and community in B-boy culture. Ethnomusicology 50, no. 3: 411–32.

**(...)**

[**Sample Number:**7]

[**Title:** Finger-to-beat coordination skill of non-dancers, street dancers, and the world champion of a street-dance competition ]

[**Source:**https://doi.org/10.3389/fpsyg.2016.00542 ]

[**Edited?** No]

[**Reason for Inclusion:**This study investigates the auditory-motor coordination (AMC) skills of street dancers, non-dancers, and a world champion, focusing on finger-to-beat synchronization. It provides empirical evidence of how dance training enhances coordination stability and resistance to unintentional pattern transitions, contributing to the understanding of sensorimotor learning in dance. The findings highlight the role of small movements (e.g., fingers) in dance performance, aligning with the broader definition of street dance as a discipline requiring precise rhythmic control.]

[**Coding Date:** 02/20/2025**]**

[**Coder:** First author]

[**Main points/impressions:**

Note. *The reference style of this dataset will be evaluated based on SAGE Harvard (https://journals.sagepub.com/author-instructions/IRS#ReferenceStyle)*

This study examines the finger-to-beat coordination skills of street dancers, non-dancers, and a world champion, using a dynamical systems approach. Key findings include:

Enhanced Stability in Dancers: Street dancers exhibited higher critical frequencies (3.3 Hz vs. 2.6 Hz in non-dancers) before transitioning from extend-on-the-beat to flex-on-the-beat patterns, indicating greater coordination stability (p. 8).

World Champion’s Unique Skill: The champion matched peak finger velocity to the beat across tempos, suggesting an advanced ability to synchronize movement dynamics with auditory cues (p. 9).

Effector Independence: Results imply that AMC skills acquired through dance training may generalize across body parts (e.g., knees to fingers), supporting the idea of a shared neural representation (p. 7).]

**Writing Purpose Statements:**

This article explores the auditory-motor coordination (AMC) skills of street dancers, non-dancers, and a world champion during finger-to-beat synchronization. By analyzing phase transitions and variability in coordination patterns, the study demonstrates how dance training enhances the ability to maintain stable rhythmic movements at high tempos. The research underscores the importance of small movements (e.g., fingers) in dance performance and suggests that sensorimotor learning in street dance may be effector-independent. For example, the text states: "The dancers had a significantly higher critical frequency than the non-dancers had during the extend-on-the-beat condition" (p. 6).

**Methodology and Data Collection (Provide Evidence to Support the Credibility of the Study’s Findings)**

This paper uses a diversity of participants in the study, including different types of participants. For example."Six skilled street dancers and six controls participated in this experiment. The street dancers (men; age: 27.8 ± 5.8 years[mean ± standard deviation (SD)]) had 8427 ± 3039 h of accumulated dance practice time (11.2 ± 6.0 years of dancing experience). One of the dancers won a celebrated international street dance competition. Hereafter, we refer to this individual as the world champion. The other five participants were not street dancing at an international level, but at a national level."

**Data Collection Methods:**
The study employed a phase transition paradigm with an increasing metronome beat rate (1.0–3.7 Hz). Participants performed finger flexion/extension synchronized to the beat while angular displacement and velocity were recorded via electrogoniometry. Data were analyzed using circular statistics to determine phase angles and variability. For example: "The angular displacement data were smoothed using a forward and back 2nd order Butterworth low-pass filter with a cut-off frequency of 10 Hz" (p. 4).

Evidence:

Quantitative: Statistical comparisons (ANOVA, Welch’s tests) confirmed group differences in critical frequencies and phase variability .

“Thus, the unpaired Welch’s tests were performed to compare the groups at each beat

rate. ...We performed a two-way analysis of variance (ANOVA) with a between-subject factor of group (dancer vs. non-dancer) and a within-subject factor of beat rate (1.0, 1.3, 1.7, 2.0, 2.3, 2.7, 3.0,3.3, and 3.7 Hz) on (i) the mean and (ii) the SD of the phase angle of the movement at the beat time.”（p.5）

**Provide Evidence to Support the Transferability of the Study’s Findings**
The findings may apply to other rhythmic tasks, as the study suggests AMC skills are not limited to specific effectors. For instance: "The learning effect that is gained from dance practice to coordinate the knee movements may be transferred to the finger coordination to a beat, and vice versa" (p. 2).

**Provide Evidence to Support the Dependability of the Study’s Findings**

Evidence:

“These results suggest that the skill of accomplished dancers not only lies in big knee movements but also in small finger movements. The phrase “God is in the details” may apply to street dance. These findings provide additional evidence that the sensorimotor learning of rhythmic dance is characterized by a stabilization of the coordination pattern, including the inhibition of an unintentional transition to other coordination patterns.”(p.9)

**Provide Evidence to Support the Dependability of the Study’s Findings**
The authors acknowledge limitations, such as the small sample size and lack of resistance instructions for the world champion: "If the participants were instructed to intentionally resist the phase transition, the results may have been different" (p. 8).

**References (Use a Specific Style Referencing)**

Alderisio, F., Bardy, B. G., and Di Bernardo, M. (2015). Entrainment and Synchronization in Heterogeneous Networks of Haken-Kelso-Bunz (HKB) Oscillators. arXiv preprint arXiv:1509.00753. Available at: http://arxiv.org/abs/1509.00753v1

Amunts, K., Schlaug, G., Jäncke, L., Steinmetz, H., Schleicher, A., Dabringhaus, A.,et al. (1997). Motor cortex and hand motor skills: structural compliance in the human brain. Hum. Brain Mapp. 5, 206–215. doi: 10.1002/(SICI)1097-0193(1997)5:3

Repp, B. H., and Su, Y.-H. (2013). Sensorimotor synchronization: a review of recent

research (2006–2012). Psychon. Bull. Rev. 20, 403–452. doi: 10.3758/s13423-

012-0371-2

Schmidt, R. C., Carello, C., and Turvey, M. T. (1990). Phase transitions and

critical fluctuations in the visual coordination of rhythmic movements between

people. J. Exp. Psychol. Hum. Percept. Perform. 16, 227–247. doi: 10.1037/0096-

1523.16.2.227

**(...)**

[**Sample Number:**8]

[**Title:** Getting Off Portrayals of Masculinity in Hip Hop Dance in Film ]

[**Source:**https://doi.org/10.2307/1477808. ]

[**Edited?** No]

[**Reason for Inclusion:**This article provides a detailed analysis of how masculinity is portrayed through hip-hop dance in film. It explores the cultural and social dimensions of hip-hop dance, particularly focusing on the evolution of its representation from street culture to mainstream media. This study is crucial for understanding the intersection of dance, gender, and media representation within hip-hop culture.]
[**Coding Date:** 02/20/2025**]**

[**Coder:** First author]

[**Main points/impressions:**
Note. The reference style of this dataset will be evaluated based on SAGE Harvard ([https://journals.sagepub.com/author-instructions/IRS#ReferenceStyle](https://journals.sagepub.com/author-instructions/IRS#ReferenceStyle))
This article examines the portrayal of masculinity in hip-hop dance, tracing its evolution from street culture to mainstream media. The study highlights how hip-hop dance, particularly breakdancing, has been used as a means of self-expression and empowerment for young men in marginalized communities. The article also discusses the competitive nature of hip-hop dance and its role in establishing male dominance and respect. Additionally, it explores how these themes are translated and sometimes altered when hip-hop dance moves from the street to the screen. ]

**Writing Purpose Statements**This article explores the evolution of masculinity portrayals in hip-hop dance, particularly focusing on its transition from street culture to mainstream media. Through an analysis of various films and music videos, the article discusses how the competitive and expressive nature of hip-hop dance is both preserved and transformed in different media contexts. For example, the text reads: “This article traces the evolution of hip hop through its various genres, from early 1980s documentary films to its first appearances in Hollywood videos and its 1990s manifestation and proliferation in television music videos” (p. 113).

**Methodology and Data collection(Provide Evidence to Support the Credibility of the Study’s Findings)**This article utilized a qualitative research design, and employed multiple data sources, focusing on the analysis of various films, documentaries, and music videos that feature hip-hop dance. The author examined how hip-hop dance is portrayed in different media formats and how these portrayals reflect and shape cultural understandings of masculinity. For example, the text reads: “The competitive layers of hip hop dancing are dissected and presented in their purest forms in Tony Silver's 1983 documentary film, Style Wars” (p. 114). Data collection involved a detailed review of films such as “Style Wars,” “Wild Style,” and “Flashdance,” as well as music videos by artists like IMX and Usher. The study also references scholarly works to support its analysis, such as those by Nelson George and Tricia Rose. For instance, the text states: “According to Foster, in hip hop ‘The competitive stakes of each performance allowed dancers to enhance their status and increase their prestige within a masculine-dominated arena’” (p. 114).

**Provide Evidence to Support the Transferability of the Study’s Findings**As evidence of the transferability of the study, the text mentions the consistent portrayal of competitive and masculine themes across different media formats. For example, “From the sidewalk to the dance hall to the screen in films and in music videos, and despite choreographed versus spontaneous hip hop dance-offs, these competitive elements remain” (p. 116). The study also highlights the influence of hip-hop dance on mainstream culture, suggesting that its themes of masculinity and competition are widely recognized and replicated. For instance, the text notes: “Hip hop made one of its earliest Hollywood appearances in Flashdance, the popular 1983 Hollywood film directed by Adrian Lyne. The hip hop dance number is only about a minute-and-a-half in length and is situated within a movie focused on jazz dance, using an almost entirely white cast. Nonetheless, the brief episode adds a touch of reality to the film and manages to insinuate the competitive roots of hip hop” (p. 115).

**Provide Evidence to Support the Dependability of the Study’s Findings**
This article presents a comprehensive analysis of multiple films and music videos, providing evidence of iterative data collection and analysis. For example, the text reads: “In a 1999 music video, ‘Stay the Night,’ the artists IMX express competitive aggression overtly through gestures and movement. In this video, the camera itself becomes the enemy, or the opposition, as IMX creates an antagonistic relationship by dramatizing movements of confrontation with it” (p. 115). The study also includes detailed descriptions of specific scenes and performances, demonstrating a thorough and consistent approach to data analysis. Another example is the analysis of the film “Wild Style,” which shows how hip-hop dance is used to convey themes of masculinity and competition: “In one scene, two crews compete on a black and white checkered dance floor as the audience, rooting loudly, and the camera encircle the performance. The crews accentuate certain body parts through costuming that includes white gloves, large white sneakers, and hats as props” (p. 115).

Some proofs about iterative data analysis, for example:“Hip hop dance-breaking and uprocking-communicate masculinity. The culture arosefrom the souls of adolescent boys;, it is like a doorway into their hearts and minds, a diary writ.ten and recorded by the body on pavement, Preserved like oral history, the stories are per.formed over and over, each time with a different twist, reflecting the individuality of each nar-.rator. It is the story of battles, of a struggle to be heard, seen, and remembered”(p.119)

**Provide Evidence to Support the Confirmability of the Study’s Findings**In the text, the author reflects on the broader implications of the study. For example, “As these films and music videos illustrate, the two components of competition in hip hop dance have persisted throughout the stages of hip hop's evolution. From the sidewalk to the dance hall to the screen in films and in music videos, and despite choreographed versus spontaneous hip hop dance-offs, these competitive elements remain” (p. 115).

**Conclusion**
The article concludes that hip-hop dance remains a powerful medium for expressing and affirming masculinity, even as it transitions from street culture to mainstream media. The study highlights the importance of competition, sexuality, and hero-worship in hip-hop dance and how these themes are adapted and preserved in different media contexts. This research provides valuable insights for scholars and practitioners interested in the intersection of dance, masculinity, and media representation. Future research could further explore how these themes evolve with the continued globalization of hip-hop culture.

**References(Use a Specific Style Referencing)**

Bing, Jonathan, Maria Simson, and Jeff Zaleski.1998.“Hip-Hop America.”Publishers Weekly(August31): 58-59.

Foster, Susan Leigh. 1998. “Choreographies of Gender.” Signs: Journal of Women in Culture andSociety 24(1):1-33.

**(...)**

[**Sample Number:**9]

[**Title:** A Cross-Sectional Study Comparing Traumatic Alopecia Among B-Boys and B-Girls to Other Dance Styles and Its Impact on Dance Performance and Health ]

[**Source:**https://doi.org/10.1177/1089313X231176598]

[**Edited?** No]

[**Reason for Inclusion:**This study investigates the unique health condition of alopecia among breakdancers (b-boys and b-girls), with a focus on the "headspin hole" caused by frequent headspins in breakdancing. It highlights the potential impact of this hair loss condition on dance performance, mental health, and overall well-being, which is crucial for understanding the intersection of street dance and health-related issues.]

[**Coding Date:** 02/20/2025**]**

[**Coder:** First author]

[**Main points/impressions:**

Note. *The reference style of this dataset will be evaluated based on SAGE Harvard (https://journals.sagepub.com/author-instructions/IRS#ReferenceStyle)*

This article investigates the prevalence of traumatic alopecia, commonly known as “headspin hole,” among breakdancers compared to other dance styles. The study highlights the significant impact of hair loss on dancers’ concerns and well-being. For example, the text reads: “This study found that there was a significant difference in hair loss among breakers compared to non-breakers” (p. 13). The study also explores the barriers to medical treatment and the effects of hair loss on dancing performance. The results indicate that breakers are less likely to seek medical attention for hair loss, which may exacerbate their concerns and health issues. For instance, the text states: “Despite these concerns, breakers were less likely to seek medical attention” (p. 13).]

**Writing Purpose Statements**

This article aims to investigate the relationship between alopecia and breaking (commonly known as breakdancing) and its impact on dancers’ concerns, performance, and health. The study explores the prevalence of hair loss among breakers compared to other dance styles and examines the barriers to medical treatment. For example, the text reads: “Given the prevalence of hair loss in this dance community and limited research conducted in this underserved population, the purpose of this study was to investigate the relationship between alopecia and breaking, the level of concern dancers have regarding hair loss, barriers to medical treatment, and how it affects their dancing” (p. 13).

**Methodology and Data collection(Provide Evidence to Support the Credibility of the Study’s Findings)**

This article utilized a cross-sectional study design with an online survey as the primary source of data. The survey addressed participants’ demographics, hair, dancing styles, training, and health history. For example, the text reads: “This was a cross-sectional study using an online survey. The survey addressed participants’ demographics, hair, dancing styles, training, and health history” (p. 13). Data collection involved disseminating the survey via social media platforms such as Facebook, Instagram, and Reddit. The study included 142 participants, with 71 breakers and 71 dancers of other styles. The results were analyzed using descriptive statistics, chi-squared analysis, independent samples t-test, and logistic regression. For instance, the text states: “Descriptive statistics including mean and standard deviation were calculated. Chi-squared analysis was conducted to examine differences between categorical variables and style of dancing (breakers vs. non-breakers)” (p. 14).

**Provide Evidence to Support the Transferability of the Study’s Findings**

As evidence of the transferability of the study, the text mentions the significant difference in hair loss among breakers compared to non-breakers. For example, “This study found that there was a significant difference in hair loss among breakers compared to non-breakers” (p. 13). The study also highlights the increased concern for hair loss among breakers, which may be applicable to other dancers who perform similar head-related movements. For instance, the text notes: “Breakers reported significantly increased concerns for hair loss compared to those who do not break (p < 0.001); 45 (63.38%) of breakers compared with 20 (28.17%) of other dancers reported hair loss concerns. Not controlling for age and sex, breakers were 4.4 times more likely to report concerns for hair loss compared to other types of dancers” (p. 15).

**Provide Evidence to Support the Dependability of the Study’s Findings**

This article presents multiple analyses, including logistic regression models, to ensure the reliability of the findings. For example, the text reads: “Multiple logistic regressions controlled for age and sex.”(p.14)

“Logistic regression showed that breakers were 2.6 times more likely to report balding than non-breakers (OR: 2.61; 95% CI: 1.27-5.34)” (p. 15). The study also controlled for age and sex in the analyses to account for potential confounders. Another example is shown in Table 1 and Table 2, which provide detailed demographic and statistical data to support the findings.

**Provide Evidence to Support the Confirmability of the Study’s Findings**

In the text, the authors reflect on the limitations and future recommendations. For example, “While our study showed that the relationship between breaking and alopecia was statistically significant, there are factors that may affect the population’s concerns about hair loss and seeking out medical help” (p. 18).

**Conclusion**

The article concludes that breakers are significantly more likely to experience alopecia compared to other dancers, which significantly affects their concerns and well-being. The study highlights the need for further research to investigate interventions to prevent and treat hair loss in this population and to address the barriers to medical care. This study provides valuable insights for dance medicine professionals and highlights the unique health challenges faced by breakdancers.

**References(Use a Specific Style Referencing)**

Samrao A, Chen C, Zedek D, Price VH. Traction alopecia in a ballerina: clinicopathologic features. Arch Dermatol. 2010;146(8):930-1.

Brankov N, Conic RZ, Atanaskova-Mesinkovska N, et al. Comorbid conditions in lichen planopilaris: a retrospective data analysis of 334 patients. Int J Womens Dermatol. 2018;4(3):180-4.

Rubin MB. Androgenetic alopecia. Battling a losing proposition. Postgrad Med. 1997;102(2):129-31, 136.

Romanova Y, Romanov D, Brazhnikov AY, et al. Perspective targets for secondary prevention of quality of life decrease and psychosomatic disorders in alopecia: results of an observational study [in Russian]. Profilakticheskaya meditsina. 2019;22(5):104-11.

Andermann A, Collaboration C. Taking action on the social determinants of health in clinical practice: a framework for health professionals. CMAJ. 2016;188(17-18):E474-83.

**(...)**

[**Sample Number:** 10]

[**Title:** Hip-Hop, Gangs, and the Criminalization of African American Culture ]

[**Source:**https://doi.org/10.1177/0021934719833396]

[**Edited?** No]

[**Reason for Inclusion:**This article critiques the pervasive gang origin narrative of hip-hop culture, highlighting its association with the criminalization of African American and Latino communities. By analyzing the oral history collection Yes Yes Y’all: The Experience Music Project Oral History of Hip-Hop’s First Decade, this material examines the discrepancies between early hip-hop practitioners' accounts and the historical framing that links hip-hop culture to gangs.]

[**Coding Date:** 02/21/2025**]**

[**Coder:** First author]

[**Main points/impressions:**

Note. *The reference style of this dataset will be evaluated based on SAGE Harvard (https://journals.sagepub.com/author-instructions/IRS#ReferenceStyle)*

This article critically interrogates the widely accepted narrative that hip-hop originated from street gangs in the Bronx. The study reveals that this narrative is not supported by the firsthand accounts of early hip-hop practitioners. The article also highlights how the editorial process behind the book “Yes Yes Y’all” framed the connection between gangs and hip-hop in misleading ways. The study concludes that the association between gangs and hip-hop is part of a larger discourse that criminalizes African American culture.]

**Writing Purpose Statements**

This article aims to critically examine the dominant narrative that hip-hop emerged from street gangs in the Bronx, New York. The study investigates how this narrative is presented in the influential book “Yes Yes Y’all” and compares it with the firsthand accounts of early hip-hop practitioners. For example, the text reads: “I critically interrogate this dominant narrative through an examination of the influential book Yes Yes Y’all: The Experience Music Project Oral History of Hip-Hop’s First Decade” (p. 298). The study also explores the broader implications of this narrative for the understanding of hip-hop culture and its historical context.

**Methodology and Data collection(Provide Evidence to Support the Credibility of the Study’s Findings)**

This article utilizes a critical analysis of the original interview transcripts used in the book “Yes Yes Y’all” and compares them with the published accounts in the book. The study examines how the editorial process framed the connection between gangs and hip-hop. For example, the text reads: “Drawing from the original interview transcripts used for YYY, I compare the gang-origin narrative espoused in the book with the primary accounts of early hip-hop practitioners featured within it” (p. 298). The study also discusses the broader historical context of how African American culture has been associated with criminality. The data were collected from the archives at the Museum of Pop Culture (MoPOP) in Seattle, WA, and the Charlie Ahearn Hip Hop Archive at Cornell University.

**Provide Evidence to Support the Transferability of the Study’s Findings**

As evidence of the transferability of the study, the text mentions the consistent pattern of framing quotes in misleading ways to draw a connection between gangs and hip-hop where none exists. For example, the text reads: “A close examination of the transcripts reveals a consistent pattern of framing quotes in misleading ways to draw a connection between gangs and hip-hop where none exists” (p. 300). The study also highlights how this narrative has been reproduced in various scholarly and academic texts on hip-hop history. For instance, the text notes: “The most common narrative, in both academia and the media, is that street gangs dominated the Bronx and eventually rechanneled their energies to create hip-hop music and its accompanying expressions” (p. 299).

“Special attention is given to the divergences between the two sources”(p. 298).

**Provide Evidence to Support the Dependability of the Study’s Findings**

This article presents a detailed analysis of the original interview transcripts and the published accounts in “Yes Yes Y’all.” The study identifies specific instances where the editorial process altered the original quotes to fit the gang-origin narrative. For example, the text reads: “The original interview with [Afrika Bambaataa] for YYY does not suggest any relationship between street gangs and hip-hop’s artistic practices” (p. 303). The study also provides supplemental appendices with extracts from the original transcripts and the published book to support its findings. Another example is shown in the analysis of DJ Disco Wiz’s interview, where the authors removed crucial qualifiers and context to create a false connection between gangs and hip-hop.In fact, BLADE points out that the former were overtly hostile toward the latter. “Gang guys would chase graffiti guys to catch them, take their paint, and spray all over them,” he is quoted as saying. In the original interview (supplemental Appendix D), BLADE goes even further, explaining how subway artists were ethnically mixed, in contrast to the gangs ”(p. 304).

**Provide Evidence to Support the Confirmability of the Study’s Findings**

In the text, the author reflects on the broader implications of the gang-origin narrative and its role in criminalizing African American culture. For example, the text reads: “The portrayal of these communities impacts everything from public policy and scholarship to esthetic practices and social movements” (p. 300). The study also discusses the potential motivations behind the propagation of this narrative, including market considerations and the desire for dramatic storytelling. For example,. “I am not suggesting that their testimony merely be presented without analysis or interpretation5 but, rather, that historical research into the culture be grounded, first and foremost, in the ideas, meanings, and experiences of those who directly lived it.”(p.312)

**Conclusion**

The article concludes that the association between gangs and hip-hop is not supported by the firsthand accounts of early hip-hop practitioners. The study highlights the need to reconsider the dominant narrative and to engage more thoroughly with the ideas, meanings, and experiences of those who directly lived the culture. The study calls for a more accurate and comprehensive understanding of hip-hop’s history, grounded in the experiences of its founders.

**References(Use a Specific Style Referencing)**

Flores, J. (1993). Rappin,’ writin,’ & breakin.’ Trotter Review, 7, 26-28.

Dimitriadis, G. (1999). Hip-hop to rap: Some implications of an historically situated

approach to performance. Text and Performance Quarterly, 19, 355-369.

**(...)**

[**Sample Number:** 11]

[**Title:** Measurement of Rhythmical Movements in Street Dance for Quantifying Movement Timing Skills]

[**Source:** https://doi.org/10.1109/WHC56415.2023.10224437]

[**Edited?** No]

[**Reason for Inclusion:**This study explores how rhythmical movements in street dance can be quantified to assess movement timing skills. It uses an experimental paradigm to measure the accuracy of movements by comparing trained dancers with novice dancers. This material offers insights into how movement proficiency can be measured in street dance through audio-motor coordination, which is critical for both learning and evaluating street dance techniques.]

[**Coding Date:** 02/21/2025**]**

[**Coder:** First author]

[**Main points/impressions:**

Note. *The reference style of this dataset will be evaluated based on SAGE Harvard (https://journals.sagepub.com/author-instructions/IRS#ReferenceStyle)*

This study aims to measure the rhythmic movements in street dance and quantify the differences in movement timing skills based on the proficiency level of the dancers. The research highlights the importance of movement timing in street dance and explores how trained dancers differ from novices in maintaining rhythm.The study also discusses the potential application of haptic feedback in dance education to improve movement timing skills.]

**Writing Purpose Statements**

This article investigates the rhythmic movements in street dance and quantifies the differences in movement timing skills between trained and novice dancers. The study aims to understand how proficiency affects the ability to maintain rhythm and explores the potential use of haptic feedback in dance education. For example, the text reads: “This study aimed to measure the rhythms of three types of movements in street dance and to quantify the differences depending on the level of proficiency” (p. 197).“This study quantifies the movement timing of three types of movement rhythms in street dance and investigates differences in movement timing by dance proficiency (Fig. 1a)”(p.198).

**Methodology and Data collection(Provide Evidence to Support the Credibility of the Study’s Findings)**

This article utilized an experimental paradigm with video capture and motion analysis using OpenPose to measure the rhythmic movements of street dancers. The study involved 20 participants, divided into trained and novice groups. For example, the text reads: “We developed an experimental paradigm to quantify the rhythms of street dancers’ movements. 20 street dancers, 10 trained and 10 novices participated in the experiment” (p. 197). Data were collected using a mirrorless SLR camera and analyzed using OpenPose to detect body joint positions. The study measured three types of rhythmic movements: on-time, fast, and slow, and compared the results between trained and novice dancers. For instance, the text states: “The measurement method employed in this study is to capture the dancer’s movements on video and analyze and estimate the posture from the 2D images using OpenPose” (p. 198).

**Provide Evidence to Support the Transferability of the Study’s Findings**

As evidence of the transferability of the study, the text mentions the significant differences in movement timing accuracy between trained and novice dancers. For example, “The results of the experiment suggested that there was a difference in the accuracy of maintaining the same movement timing in time and slow when comparing trained and novice dancers” (p. 197). The study also highlights the potential for using haptic feedback to improve movement timing skills in dance education, suggesting broader applications for similar interventions.

Sample evidence:“Twenty participants (five females) joined the experiment.In order to identify differences in the timing of the three types of rhythms according to proficiency in street dance, we defined trained and novice dancers as follows, and recruited participants from each group. As the trained group, dancers who had won prizes in world competitions or had more than 10 years of street dance experience were asked to participate in the experiment. As a result, 10 trained participants (7 males and 3 females, 25.9 ± 4.0 years old) participated in the experiment. Trained participants had an average of 14.7 ± 5.3 years of street dance experience. For the novice group, dancers with less than three years of street dance experience were asked to participate in the experiment. As a result of the experimental recruitment, 10 novice learners (8 males and 2 females, mean age 23.0 ± 5.2 years) participated in the experiment. The average street dance experience of novice dancers was 1.6 ± 0.8 years.”(p.198)

“Video recordings of the experiment participants’ body movements were analyzed using OpenPose, an image analysis environment for posture estimation, to detect body joint positions. OpenPose is a motion tracking software using 2D images proposed by Cao et al. and published as the OpenPose library. OpenPose estimates the joint positions of multiple persons in an image or video. Posture estimation with OpenPose has been used in research in a variety of fields and has recently been used to analyze dance movements. The training dataset for estimating joint positions in OpenPose used the COCO model, which estimates 18 points, including shoulder, elbow, and wrist.”(p.199)

**Provide Evidence to Support the Dependability of the Study’s Findings**

This article presents multiple analyses, including statistical tests and circular histograms, to ensure the reliability of the findings. For example, the text reads: “A mixed ANOVA showed that the tempo of the sound (TEMPO) factor, rhythmical timing of movement (TIMING) factor, and the interaction between TEMPO and TIMING were statistically significant” (p. 200). The study also used Bonferroni correction to adjust for multiple comparisons, ensuring the robustness of the results. Another example is shown in Table I, which provides detailed statistical analysis results to support the findings.

**Provide Evidence to Support the Confirmability of the Study’s Findings**

In the text, the authors reflect on the limitations and future recommendations. For example, “The experimental paradigm proposed by this study does not measure overall dance ability. However, continuing similar attempts will lead to the development of a machine vision system that will immediately provide objective decision-making materials for human evaluators to make intuitive judgments when viewing physical expressions” (p. 201). Additionally, the study was reviewed by multiple co-authors to ensure its validity and reliability.

**Conclusion**

The article concludes that trained street dancers exhibit greater accuracy and consistency in maintaining rhythmic movements compared to novices. The study highlights the potential for using haptic feedback in dance education to improve movement timing skills. For example, the text reads: “The results indicate that machine vision can play a role in quantifying the skill with which humans can express some information through their body movements” (p. 200). This study provides valuable insights for dance educators and researchers interested in the application of technology in dance training and performance.

**References(Use a Specific Style Referencing)**

R. Donchev, E. Pescara, and M. Beigl, “Investigating retention in passive haptic learning of piano songs,” Proc. ACM Interact. Mob. Wearable Ubiquitous Technol., vol. 5, no. 2, jun 2021.

G. Grindlay, “Haptic guidance benefits musical motor learning,” in 2008 Symposium on Haptic Interfaces for Virtual Environment and Teleoperator Systems, 2008, pp. 397–404.

S. Holland, A. Bouwer, and O. Hodl, ¨ Haptics for the Development ofFundamental Rhythm Skills, Including Multi-limb Coordination. Cham: Springer International Publishing, 2018, pp. 215–237

**(...)**

[**Sample Number:** 12]

[**Title:** Moonwalking in Beijing: Michael Jackson, piliwu, and the origins of Chinese hip-hop]

[**Source:** https://doi.org/10.1080/14649373.2022.2064610]

[**Edited?** No]

[**Reason for Inclusion:**This study discusses the origin of Chinese hip-hop dance, piliwu, which integrated various U.S. urban dance styles, including b-boying, popping, and locking, into a localized Chinese version. The article emphasizes Michael Jackson's influence on Chinese hip-hop culture and how piliwu emerged in the 1980s, driven by both Western media and local performance networks.This article is extremely important for the reconstruction of the definition of street dance, because the formation of the cognitive generation gap of street dance has a lot to do with the different cultural backgrounds, for example, in this article, “piliwu” is the special name of street dance in China. For example, “piliwu” in this paper is the special name of street dance in China. This situation does not only occur in China, but also in Japan, Korea and other places where the term street dance has different names.]

[**Coding Date:** 02/21/2025**]**

[**Coder:** First author]

[**Main points/impressions:**

Note. *The reference style of this dataset will be evaluated based on SAGE Harvard (https://journals.sagepub.com/author-instructions/IRS#ReferenceStyle)*

This article examines the emergence of “piliwu” (a Chinese term for breakdancing) in the 1980s and its connection to Michael Jackson, arguing that Jackson played a central role in shaping China’s first localized hip-hop movement.The article concludes that Michael Jackson’s image and dance moves were key inspirations for Chinese hip-hop artists, despite the limited availability of his music and videos in China during the 1980s.**]**

**Writing Purpose Statements**

This article aims to reconstruct the history of the piliwu movement in China and analyze its connection to Michael Jackson and the origins of Chinese hip-hop. The study investigates how Jackson’s dance style influenced Chinese hip-hop culture through the film Rock Youth and the performances of dancers like Tao Jin. For example, the text reads: “This article follows the career of Tao Jin, China’s first hip-hop dance star, and analyzes piliwu dance choreography recorded in Tao’s debut film, the 1988 Chinese dance film Rock Youth” (p. 302). The study also explores the broader cultural context of China’s Reform Era and the role of Hong Kong in mediating U.S. popular culture.

**Methodology and Data collection(Provide Evidence to Support the Credibility of the Study’s Findings)**

This article utilizes a combination of film analysis, historical records, and interviews with key figures involved in the piliwu movement. The study examines the dance choreography in the film Rock Youth and compares it with Michael Jackson’s performances to identify similarities and influences. For example, the text reads: “A comparison of dance scenes in Rock Youth, Breakin’, and Jackson’s dancing shows that Jackson may have had more of an impact on the film’s choreography than Breakin’, even though the influences of both are clearly present” (p. 305). The study also draws on interviews with dancers who participated in the piliwu phenomenon, providing firsthand accounts of how Jackson’s style was introduced and adapted in China.

**Provide Evidence to Support the Transferability of the Study’s Findings**

As evidence of the transferability of the study, the text mentions the widespread influence of Michael Jackson’s dance style on Chinese hip-hop culture, despite the limited availability of his music and videos in China during the 1980s. For example, the text reads: “During the mid-1980s, a time when Chinese audiences had limited direct access to Jackson’s music and videos, they nevertheless “encountered” Jackson indirectly through the performances of artists like Tao Jin, who incorporated aspects of Jackson’s image and movement style into their own.” (p. 305). The study also highlights how the underground commercial dance economy known as “zouxue” facilitated the spread of Jackson’s dance styles in China. For example,“Through an informal concert economy known as “zouxue” (“moonlighting”) that emerged in China during the mid-1980s, dancers like Tao Jin disseminated Jackson’s dance styles

before they were available widely in the mass media. It was this localized, indirect experience of Michael Jackson that found its way into Rock Youth and the piliwu movement.” (p. 305).This suggests that similar cultural phenomena could occur in other regions where popular culture is transmitted through informal networks and live performances.

**Provide Evidence to Support the Dependability of the Study’s Findings**

This article presents a detailed analysis of the film Rock Youth and the piliwu movement, supported by historical records and interviews with key figures. For example, the text reads: “The ample references to Jackson that appear in Rock Youth present several conundrums to the dance historian. First, if Jackson’s music and dance videos were not widely available in China until after 1988, how did they find such a strong presence in the dance choreography, costuming, and bodily repertoires presented in Rock Youth?” (p. 309). The study addresses these questions by examining the role of dancers like Tao Jin and Zhang Ping, who traveled to Hong Kong and brought back Jackson’s dance styles through informal networks. The study also provides specific examples of Jackson’s dance moves and choreographic structures that appear in Rock Youth, supporting its claims with detailed analysis and visual evidence.

**Provide Evidence to Support the Confirmability of the Study’s Findings**

Although the text mentions something like reflection, it only states the impact that Michael Jackson has brought to Chinese pop culture, for example, “Jackson himself embodied one of the strongest representations of urban youth rebellion available in China in 1984, when he first entered popular consciousness” (p. 314). The author does not conduct a reflective study of the research.The study concludes that Michael Jackson’s influence on Chinese hip-hop culture was significant, despite the limited formal distribution of his music and videos in China during the 1980s.That is all.

**Conclusion**

The article concludes that Michael Jackson played a central role in shaping China’s first localized hip-hop movement through his dance style and image. The study highlights the importance of informal networks and live performances in transmitting popular culture across borders and the agency of local performers in adapting and disseminating new dance styles.This study provides valuable insights into the transnational flow of popular culture and the unique cultural context of China’s Reform Era, challenging conventional narratives about the origins of Chinese hip-hop.

**References(Use a Specific Style Referencing)**

Li, Yilan 李邑兰. 2009. “迈克尔·杰克逊和我们的青春记忆” [Michael Jackson and Our Memories of Youth].中国新闻周刊 [China Newsweek] 24: 34–36.

Liang, Lun 梁伦. 1985.“坚持走具有中国特色的社会主义舞蹈艺术道路” [Persist in the Path of Socialist Dance Art with Chinese Characteristics]. 舞蹈 [Dance] 2: 19–21.

**(...)**

[**Sample Number:** 13]

[**Title:** Social Resources for Positive Psychosocial Health: Youths’ Narratives of a Street Dance Performing Arts Program]

[**Source:** https://doi.org/10.1080/01488376.2020.1725715]

[**Edited?** No]

[**Reason for Inclusion:**This study focuses on how a street dance performing arts program (MINDJAM) promotes positive psychosocial health among young people in Hong Kong. The participants’ narratives provide valuable insights into the psychosocial benefits of engaging in street dance and how such programs facilitate social capital building, prosocial attitudes, and self-efficacy.]

[**Coding Date:** 02/21/2025**]**

[**Coder:** First author]

[**Main points/impressions:**

Note. *The reference style of this dataset will be evaluated based on SAGE Harvard (https://journals.sagepub.com/author-instructions/IRS#ReferenceStyle)*

This article investigates the social mechanisms through which a street dance performing arts program, MINDJAM, promotes positive psychosocial outcomes among young people in Hong Kong. The study identifies five key themes through thematic analysis of in-depth interviews with 22 participants aged 13 to 18. For example, the text reads: “The positive psychological outcomes identified are: happiness, prosocial attitudes, and self-efficacy” (p. 3). The study highlights the importance of social networks, shared goals, and positive interactions with instructors in enhancing the psychosocial well-being of participants.**]**

**Writing Purpose Statements**

This article aims to understand the underlying social processes that lead to psychosocial health improvements in young people participating in the MINDJAM street dance performing arts program. The study explores how social resources can be generated through street dance activities to promote positive outcomes among youths. For example, the text reads:"This study focuses on the use of extra-curricular activities to improve the psychosocial health of youths attending stigmatized schools in Hong Kong." (p. 1).

"This article explores and identifies the social processes underlying a 12-month youth street dance performing arts program, MINDJAM, in promoting positive psychosocial outcomes among young people in Hong Kong" (p. 1).

**Methodology and Data collection(Provide Evidence to Support the Credibility of the Study’s Findings)**

This article utilizes a qualitative research design with in-depth interviews as the primary source of data. The study interviewed 22 participants aged 13 to 18 who were part of the MINDJAM program. For example, the text reads: “We conducted in-depth interviews with 22 participants aged 13 to 18 years old who were MINDJAM participants” (p. 1). Data were analyzed using thematic analysis to identify key themes related to social processes and psychosocial outcomes. The study also provides detailed descriptions of the MINDJAM program, including its structure, activities, and goals. For instance, the text states: “The program consists of a set of sequenced activities with weekly rehearsals and seven public performances, all led by dance instructors with youth work experience” (p. 4).

**Data collection:**

“Thematic analysis was employed to examine and identify themes within the qualitative data of the in-depth interviews (Marshall & Rossman, 2011).

Words and phrases relevant to the research questions were highlighted and subsequently generated into codes; they were then categorized into themes. The data analysis was conducted by the first and second authors of this study. Identified themes were triangulated and discussed among the two researchers and the research assistant to increase the reliability and validity of the findings. Data generated from the semi-structured interviews and self-reflective logs were managed using the software NVivo. Finally, selected quotations were translated from Chinese into English. The quotations were back-translated to ensure their meaning was not lost in translation.”(p.4)

**Provide Evidence to Support the Transferability of the Study’s Findings**

As evidence of the transferability of the study, the text mentions the positive psychosocial outcomes observed in participants, such as increased happiness, prosocial attitudes, and self-efficacy. For example, the text reads: “The results of this study reveal the underlying social mechanism of a street dance performing arts program in Hong Kong. The findings suggest that MINDJAM improves the psychosocial health of participants in terms of their level of happiness, prosocial attitudes, and self-efficacy. These positive findings lend support to the program design of MINDJAM. With the aim to anchor youth engagement, these core components of the youth performing arts program are: regular rehearsals, performances, and positive socialization experiences with program instructors” (p. 8). We have reason to believe that similar street dance programs could be effective in promoting positive outcomes among young people in other regions or contexts.

**Provide Evidence to Support the Dependability of the Study’s Findings**

This article presents detailed thematic analysis of the interview data, supported by direct quotes from participants. For example, the text reads: “Interviewee12 stated that he felt happier as he spent more time with other MINDJAM peers” (p. 5). The study identifies five key themes related to social networks, shared goals, and instructor interactions, providing a comprehensive understanding of the social processes involved. The study also discusses the implications of these findings for future youth interventions and program design. For instance, the text states: “These findings serve as a pre-formative stage for future quantitative intervention research” (p. 2).

**Provide Evidence to Support the Confirmability of the Study’s Findings**

In the text, the authors reflect on the broader implications of the study and the potential for using street dance programs to improve youth psychosocial health. For example, the text reads: “Youth workers may consider harnessing the power of performing arts to engage at-risk youths in a meaningful way in the future” (p. 9). The study concludes that the findings provide valuable insights for practitioners and policymakers interested in promoting positive youth development through extracurricular activities.

**Conclusion**

The article concludes that the MINDJAM program effectively promotes positive psychosocial outcomes among young people through expanded social networks, shared goals, and positive interactions with instructors. The study highlights the potential of street dance performing arts programs as a new and effective platform for youth engagement. For example, the text reads: “Street dance performing arts programs can be a new and effective platform for youth engagement, as they are trendy, cool, and are gaining increasing popularity among younger generations” (p. 8). The findings suggest that future youth programs should consider incorporating similar elements to promote positive outcomes among participants.

**References(Use a Specific Style Referencing)**

Clay, A. (2006). “All I need is one mic”: Mobilizing youth for social change in the post-civil rights era. Social Justice, 33(2 (104), 105–121.

**(...)**

[**Sample Number:** 14]

[**Title:** Theorising hip-hop dance in the Philippines: blurring the lines of genre, mode and dimension]

[**Source:** https://www.researchgate.net/profile/J-Lorenzo-Perillo/publication/287752560_Theorising_hip-hop_dance_in_the_Philippines_Blurring_the_lines_of_genre_mode_and_dimension/links/6205e867afa8884cabd85279/Theorising-hip-hop-dance-in-the-Philippines-Blurring-the-lines-of-genre-mode-and-dimension.pdf]

[**Edited?** No]

[**Reason for Inclusion:**This material discusses the theorization of Hip-hop dance within the context of the Philippines, emphasizing how dancers in Manila interpret genres, modes, and dimensions. It offers a framework that helps us understand local adaptations of global Hip-hop culture and the evolving discourse surrounding street dance in the region.]

[**Coding Date:** 02/22/2025**]**

[**Coder:** First author]

[**Main points/impressions:**

Note. *The reference style of this dataset will be evaluated based on SAGE Harvard (https://journals.sagepub.com/author-instructions/IRS#ReferenceStyle)*

This article examines the ways in which hip-hop dancers in Manila theorize their practices through four main aspects—genre, mode, dimension, and conflict. The study highlights how these aspects blur traditional lines and contribute to a deeper understanding of hip-hop dance in the Philippines. The study also discusses the implications of these findings for contemporary popular music and performance in Asia and beyond.]

**Writing Purpose Statements**

This article aims to explore the principles of meaning-making in contemporary hip-hop performance in the Philippines by examining how dancers theorize their practices through genre, mode, dimension, and conflict. The study seeks to challenge traditional categorizations and offer a more nuanced understanding of hip-hop dance. For example, the text reads: “This essay privileges the ways Hip-hop dancers in Manila theorise their practices through four main aspects—genre, mode, dimension and conflict—in order to draw attention to the principles of meaning-making in contemporary Hip-hop performance” (p. 69).

**Methodology and Data collection(Provide Evidence to Support the Credibility of the Study’s Findings)**

This article utilizes interviews and participant observation conducted in the Philippines between February and October 2011. The study involved in-depth interviews with hip-hop dancers in Manila, focusing on their backgrounds, practices, and thoughts on Filipino gender relations within their dance practices. For example, the text reads: “This article advances its argument largely from interviews and participant observation that I accomplished in the Philippines between February and October 2011” (p. 74). The study also engaged in dance classes across different studio-dance crew partnerships to gain deeper insights into the local hip-hop dance culture. For example, the text reads:“I decided to enrol into dance classes across three different studio-dance crew partnerships. These classes allowed me greater access to possible interviewees. Living and dancing (and commuting) in Manila for an extended amount of time allowed me to establish conceptual frameworks about Hip-hop performance in folk terms—naming practices, use-value, and intertextuality inductively collected from fieldwork practices—and in academic terms drawn from existing scholarly research on Hip-hop, dance and the Philippines”(p. 75).

**Provide Evidence to Support the Transferability of the Study’s Findings**

As evidence of the transferability of the study, the text mentions the broader implications of the findings for contemporary popular music and performance in Asia and beyond. This article relates to transferability, for example, “this article has sought to outline the various ways that, seemingly unrelated, Hip-hop dance is practiced. Dancers use the pliancy of genre to place themselves within and beyond the categorical boundaries of Hip-hop."(p.96)

**Provide Evidence to Support the Dependability of the Study’s Findings**

This article presents a detailed analysis of the four main aspects—genre, mode, dimension, and conflict—through which hip-hop dancers in Manila theorize their practices. For example, the text reads: “Dancers use the pliancy of genre to place themselves within and beyond the categorical boundaries of Hip-hop” (p. 96). The study also discusses various modes of activity, such as kompet (competition), raket (commercial), klase (educational), and konsept (artistic vision), providing specific examples and quotes from interviews to support its findings. For instance, the text states: “Kompet in Manila also falls in line with what Joseph Schloss identifies as ‘the purist way to engage with the issues’ in b-boy communities” (p. 85).

**Provide Evidence to Support the Confirmability of the Study’s Findings**

In the text, the author reflects on the broader implications of the study and the potential for using hip-hop dance to promote social justice. For example, the text reads: “Only then can we dance to a tempo that stresses everyday and institutional social justice for all” (p. 96). The study concludes that recognizing the complexities of hip-hop dance practices in the Philippines can contribute to a deeper understanding of the culture and its impact on Filipino identity.

**Conclusion**

The article concludes that hip-hop dance in the Philippines is a complex and multifaceted practice that cannot be fully understood through traditional categorizations. The study highlights the importance of recognizing the internal discourses and local contexts of hip-hop dance to promote a more nuanced understanding of the culture.The findings suggest that future research should continue to explore the intersections of genre, mode, dimension, and conflict in hip-hop dance to better understand its global impact.

**References(Use a Specific Style Referencing)**

Lack of reference

**References**

**(...)**

[**Sample Number:** 15]

[**Title:** Towards a Chinese Hip-hop Feminism and a Feminist Reassessment of Hip-hop with Breakdance: B-girling in Hong Kong, Taiwan and China]

[**Source:** https://doi.org/10.1080/10357823.2019.1631256]

[**Edited?** No]

[**Reason for Inclusion:**This sample explores the intersection of hip-hop feminism, gender studies, and street dance, with a focus on B-girling (breakdance) practices in Hong Kong, Taiwan, and China. It presents data on the evolution of breakdancing as a gendered practice and its place within hip-hop culture in East Asia.]

[**Coding Date:** 02/22/2025**]**

[**Coder:** First author]

[**Main points/impressions:**

Note. *The reference style of this dataset will be evaluated based on SAGE Harvard (https://journals.sagepub.com/author-instructions/IRS#ReferenceStyle)*

This article examines the gender dynamics within the breakdancing community in Chinese societies, focusing on the experiences of b-girls in Hong Kong, Taiwan, and China. The study identifies six major types of gender inequalities faced by b-girls, including thin-body ideals, risk of infertility, masculine dance language, indecent exposure in dress, exclusion from breaker networks, and industrial exclusion.The article argues that these challenges are rooted in traditional Chinese patriarchal culture and differ significantly from those faced by women in the US hip-hop scene.**]**

**Writing Purpose Statements**This article aims to explore the gender inequalities confronting women who practice breakdancing in Chinese societies and to propose the concept of Chinese hip-hop feminism. The study seeks to document the struggles of b-girls against gender inequality and to illustrate how these challenges differ from those found in the US context. For example, the text reads: “We outline the contours of a Chinese hip-hop feminism and suggest adopting it to investigate the local specificities of hip-hop-based gender politics in Chinese societies” (p. 1).

**Methodology and Data collection(Provide Evidence to Support the Credibility of the Study’s Findings)**This article utilizes in-depth interviews and participant observation with 15 professional b-girls in Hong Kong, Taiwan, and China. The study also includes informal interviews conducted during participant observation. For example, the text reads: “This article adopts in-depth interviewing, participant observation and informal interviewing methods. Data collection was carried out in 2010, 2014 and 2015” (p. 6).“he conducted informal interviews during participant observation and through online means. These interviews lasted between 10 and 90 minutes. Among these secondary informants were two globally established b-girls, 30 professional and non-professional b-girls, and 15 professional b-boys.”（p.7） The study identifies six major types of gender inequalities faced by b-girls, including thin-body ideals, risk of infertility, masculine dance language, indecent exposure in dress, exclusion from breaker networks, and industrial exclusion. The findings are supported by detailed accounts from the interviews and observations.

**Provide Evidence to Support the Transferability of the Study’s Findings**As evidence of the transferability of the study, the text mentions the potential for these findings to inform the development of other local hip-hop feminisms. For example, the text reads: “Moreover, even if vestiges of regional difference

had existed, our inclusion of Beijing, Zhengzhou and Guangzhou/Shenzhen ensured

that we covered the North, Central and South China regions.” (p. 7). The study suggests that the concept of Chinese hip-hop feminism could be applied to other regions with similar cultural contexts.

**Provide Evidence to Support the Dependability of the Study’s Findings**
This article presents detailed findings based on interviews and participant observation, and informal interviews with b-girls in Hong Kong, Taiwan, and China. For example, the text reads: “We identified six kinds of gender inequality and struggle associated with each of these. They concern i) thin-body ideals, ii) risk of infertility, iii) masculine dance language, iv) indecent exposure in dress, v) exclusion from breaker networks, and vi) industrial exclusion of b-girls” (p. 7). The study provides specific examples and quotes from interviews to support each of these findings. For instance, the text states: “Informant E, who has a considerably thicker upper back and arms than most Hong Kong young women, is ‘troubled by the body shaming constantly hurled at [her]’” (p. 8).

**Provide Evidence to Support the Confirmability of the Study’s Findings**
In the text, the authors reflect on the broader implications of the study for the internationalization of hip-hop feminist studies. For example, the text reads: “We find that culture is a key dimension on which Chinese hip-hop-based gender politics focuses, but that race is not. This and other possible local specificities prompt two thorny theoretical questions: whether Chinese hip-hop feminist studies are a part of hip-hop feminism, and how to evaluate Chinese hip-hop feminism’s displacement of traditional culture.We invite future debates on these questions, which in our view are generalizable to different localities across the globe and hence crucial to the internationalization of hip-hop feminism” (p. 16). The study concludes that the concept of Chinese hip-hop feminism could contribute to a more nuanced understanding of hip-hop and gender politics in non-Western contexts.

**Conclusion**
The article concludes that b-girls in Chinese societies face unique gender inequalities rooted in traditional patriarchal culture. The study proposes the concept of Chinese hip-hop feminism to address these challenges and suggests that future research should explore other local specificities of hip-hop and gender politics. The findings highlight the need for further research on the intersectionality of gender, culture, and hip-hop in Chinese contexts.

**References(Use a Specific Style Referencing)**

Leung, F., Lam, S., & Sse, S. (2001). Cultural expectations of thinness in Chinese women. EatingDisorders, 9(4), 339–350.

Lindsey, T. B. (2015). Let me blow your mind: Hip-hop feminist futures in theory and praxis.Urban Education, 50(1), 52–77.

Ma, E. (2002). Translocal spatiality. International Journal of Cultural Studies, 5(2), 131–152.

**(...)**

[**Sample Number:** 16]

[**Title:** Walking histories: narratives of young urban dancers in the city of Rio de Janeiro (Brazil)]

[**Source:** https://doi.org/10.1080/14647893.2024.2413642]

[**Edited?** No]

[**Reason for Inclusion:** This article investigates the construction of street dances in Rio de Janeiro, Brazil, focusing on the narratives of young urban dancers. It provides insights into how these dancers navigate their identities, cultural practices, and the broader social context. The study highlights the interplay between masculinity, dance, and cultural authenticity, making it a valuable resource for understanding the complexities of urban dance cultures.]

[**Coding Date:**02/22/2025**]**

[**Coder:** First author]

[**Main points/impressions:**

Note. *The reference style of this dataset will be evaluated based on SAGE Harvard (https://journals.sagepub.com/author-instructions/IRS#ReferenceStyle)*

This article delves into the lived experiences of young urban dancers in Rio de Janeiro, Brazil, exploring how they construct their identities through dance. It examines the relationship between street dance, masculinity, and cultural authenticity, highlighting the importance of understanding the socio-cultural context in which these dancers operate. The study emphasizes the role of dance in shaping personal narratives and the broader cultural landscape.**]**

**Writing Purpose Statements**
This article aims to investigate the construction of street dances in Rio de Janeiro, Brazil, by focusing on the narratives of young urban dancers. It seeks to understand how these dancers navigate their identities, cultural practices, and the broader social context. The study highlights the interplay between masculinity, dance, and cultural authenticity, providing insights into the complexities of urban dance cultures. For example, the text reads: “The aim of the study is to investigate the construction of street dances in the city of Rio de Janeiro, describing urban dancers and their daily 'art of doing', listening to their voices and observing their physical and virtual interactions.” (p. 1)

**Methodology and Data collection (Provide Evidence to Support the Credibility of the Study’s Findings)**
This article utilizes a qualitative research design, focusing on the narratives of eleven young male dancers in Rio de Janeiro. The researchers conducted semi-structured interviews and analyzed the dancers' daily experiences, personal life stories, and interactions. The study also incorporates ethnomethodological perspectives to understand how these dancers construct their social narratives. For example, the text reads: “The research focuses on eleven sets of narratives analysed in order to gather stories of male dancers aged between 18 and 32, all presented under pseudonyms.” (p. 3) Data collection involved following the dancers' online interactions and conducting interviews in various urban spaces. The study's credibility is supported by its in-depth analysis of the dancers' experiences and the use of multiple data sources. For example, the text reads: “The strategy was to register a personal profile on Facebook and start searching within this haptic space... Finally, numbers were lost of the quantity of friend requests, but accepted by about 50 dancers.” (p.4)

**Provide Evidence to Support the Transferability of the Study’s Findings**The study's findings are transferable to other urban contexts where street dance cultures thrive. The text mentions that the experiences of these young dancers in Rio de Janeiro can provide insights into the broader dynamics of urban dance cultures. For example, the text reads: “The narratives of these young men have provided an effective means for representing hip-hop dancers’ concerns and bodily articulations in order to comprehend their dance practice and the construction of spaces, but also indicate aesthetic and commercial dimensions.” (p. 2) “This paper begins with a discussion of studies of Leal (2007) and Herschmann (2000),which give us keys to understand the urban dance phenomenon within the universe of hip-hop culture in Rio de Janeiro.”(p. 2) The study's focus on masculinity, cultural authenticity, and social interactions makes its findings relevant to other urban dance communities.

**Provide Evidence to Support the Dependability of the Study’s Findings**The researchers used a combination of ethnomethodological perspectives and phenomenological approaches to understand the dancers' experiences. For example, the text reads: “As general theoretical framework the ethnomethodological perspective of Garfinkel (1967) was applied, an approach interested in social action, intersubjectivity and linguistic communication.” (p. 2) The study also incorporates the concept of performativity from Butler (1988) to understand the construction of gender identities through dance. The researchers' use of multiple theoretical frameworks and iterative data analysis supports the dependability of the study's findings.

**Provide Evidence to Support the Confirmability of the Study’s Findings**The authors reflect on the study's findings and the broader implications for understanding urban dance cultures. For example, the text reads: “The issue of masculinity came up mainly as a parental concern about the fact that dance puts them in place within a subordinate masculinity...” (p. 9) The study's findings are confirmed through the dancers' narratives and the researchers' analysis of their experiences. The authors also highlight the importance of further research to explore the complexities of urban dance cultures. For example, the text reads: “This paper has revealed the complexity of hip-hop and street-dance culture in general, and specifically among young Brazilians.” (p. 10)

**Conclusion**The article concludes that the narratives of young urban dancers in Rio de Janeiro provide valuable insights into the construction of street dances and the broader cultural context. The study highlights the importance of understanding the interplay between masculinity, dance, and cultural authenticity. The findings suggest that urban dance cultures are dynamic and multifaceted, influenced by both local and global dynamics. This study provides important insights for researchers and practitioners interested in understanding the complexities of urban dance cultures.

**References(Use a Specific Style Referencing)**

Butler, J. (1988). Performative Acts and Gender Constitution: An Essay in Phenomenology and Feminist Theory. Theatre Journal, 40(4), 519-531.

Buzo, A. 2010. Hip-hop: dentro do movimento. Rio de Janeiro: Aeroplano.

Connell, R.W., and J. W. Messerschmidt. 2013. Masculinidade hegemônica: repensando o conceito.Estudos Feministas 21 no. 1: 241-282.

**(...)**

[**Sample Number:**17]

[**Title:**A study on image expressions for augmenting street dances and their matching ]

[**Source:**https://doi.org/10.3756/artsci.14.36 ]

[**Edited?** No]

[**Reason for Inclusion:**This article explores the integration of video projection and image expressions to enhance street dance performances. It provides valuable insights into how technology can be used to augment the visual appeal of dance performances, particularly focusing on break dance, lock dance, and pop dance. The study offers practical methods and theoretical frameworks for creating interactive and visually engaging dance performances.]

[**Coding Date:** 02/22/2025**]**

[**Coder:** First author]

[**Main points/impressions:**

Note. *The reference style of this dataset will be evaluated based on SAGE Harvard (https://journals.sagepub.com/author-instructions/IRS#ReferenceStyle)*

This article investigates the use of video projection and image expressions to augment street dance performances, particularly focusing on break dance, lock dance, and pop dance. The study explores two types of image expressions—“Track” and “Flash”—and evaluates their effectiveness in enhancing the visual appeal of these dance genres.]

**Writing Purpose Statements**
This article aims to explore the integration of video projection and image expressions to enhance the visual appeal of street dance performances. It focuses on developing image expressions that can dynamically respond to the movements of dancers, thereby creating a more engaging and immersive experience for the audience. The study also seeks to understand the compatibility of these image expressions with different street dance genres. For example, the text reads: “The authors are studying video projection intended to augment the appeal of dance performance from the latter of these two approaches” (p. 36). “As the subject of this study, focusing on break dance, lock dance, and pop dance as three subgenres of street dance, this paper reports the development of two types of image expression systems for responding automatically to the movements of dancers and experiential knowledge obtained from studying the affinity between these systems and each genre” (p. 36).

**Methodology and Data collection (Provide Evidence to Support the Credibility of the Study’s Findings)**
This paper lacks a description of the sampling strategy, or even a detailed description of the sample, and instead focuses the study on three styles of breakdancing: breaking, locking, and popping.However,this article utilizes a qualitative research design, focusing on the development and testing of two types of image expressions—“Track” and “Flash”—to enhance street dance performances. The researchers used Kinect to capture dancers’ movements and developed a projection system to generate real-time video images. The study involved multiple test trials and feedback from dancers and audience members to refine the system. For example, the text reads: “We used Kinect to obtain data on dancers’ movements. A Kinect device combines features such as a depth sensor and a red-green-blue camera into a relatively compact size.” (p. 38) Data collection involved observing the dancers’ performances and collecting feedback from both the performers and the audience. The study’s credibility is supported by its detailed methodology and iterative testing process. For example, the text reads: “In the testing stage, we improved the system based on the responses of dancers and audience members.” (p. 42)

**Provide Evidence to Support the Transferability of the Study’s Findings**
The study’s findings are transferable to other dance genres and performance contexts. The text mentions that the developed image expressions and projection system can be applied to a broader range of dance performances, including traditional dances like Noh and classical ballet. For example, the text reads: “Aiming to develop a general-purpose system in the future, we would like both to put to use the knowledge obtained from this study and to seek out a route toward applying it to a broader range of dance, including traditional performances such as Noh and classical ballet.” (p. 44)

**Provide Evidence to Support the Dependability of the Study’s Findings**
The researchers used Kinect to capture dancers’ movements and developed a projection system to generate real-time video images. The study involved multiple test trials and feedback from dancers and audience members to refine the system. For example, the text reads: “we developed the system as outlined below.We used Kinect to obtain data on dancers’ movements. A Kinect device combines features such as a depth sensor and a red-green-blue camera into a relatively compact size.” (p. 38) The study also includes detailed descriptions of the image generation processes for both “Track” and “Flash” expressions, providing a clear and replicable methodology. For example, the text reads: “To generate video focused on the silhouette, we created images based on data on the dancer’s silhouette and depth data available from Kinect.” (p. 39)

**Provide Evidence to Support the Confirmability of the Study’s Findings**
The authors reflect on the study’s findings and suggest future improvements and applications. For example, the text reads: “As future improvements in the system, we are interested in the following points: - Consideration of improving the accuracy of capturing motion and video generation by introducing a small-sized, lightweight and wireless acceleration sensor.” (p. 44) The study’s findings are confirmed through the positive feedback from dancers and audience members, indicating that the developed system can enhance the visual appeal of dance performances. For example, the text reads: “From this questionnaire we were able to obtain positive evaluations from more than 80% of subjects on all assessment items for both patterns generated.” (p. 42)

**Conclusion**
The article concludes that the developed image expressions and projection system can significantly enhance the visual appeal of street dance performances. The study provides practical methods and theoretical frameworks for integrating technology with dance performances, particularly focusing on break dance, lock dance, and pop dance. The findings suggest that the developed system can be applied to a broader range of dance genres and performance contexts. This study provides important insights for researchers and practitioners interested in exploring the intersection of technology and performing arts.

**References(Use a Specific Style Referencing)**

[7] KAGEMU - BLACK SUN -，

http://www.youtube.com/watch?feature=player_embedded&v=Qvoh0

ZtVIW8(cited 2015.05.07).

[8] enra “pleiades”,

https://www.youtube.com/watch?v=0813gcZ1Uw8 (cited 2015.05.07)

(...)

[**Sample Number:** 18]

[**Title:** Expressing Joy Through Hip-Hop Dance Steps: Focus on New Jack Swing]

[**Source:** https://doi.org/10.3756/artsci.14.36]

[**Edited?** No]

[**Reason for Inclusion:** This study investigates how emotion, specifically joy, can be expressed through hip-hop dance steps, with a focus on the new jack swing. It provides valuable insights into the relationship between dance movements and emotional expression, using three-dimensional motion analysis to quantify and differentiate dance steps based on the presence of emotion. The findings contribute to the understanding of how dance can convey emotions and have practical applications in dance education.]

[Coding Date: 02/23/2025]

[Coder: First author]

[**Main points/impressions:**

Note. The reference style of this dataset will be evaluated based on SAGE Harvard (https://journals.sagepub.com/author-instructions/IRS#ReferenceStyle)

This study explores the expression of joy through hip-hop dance steps, specifically focusing on the new jack swing. Using three-dimensional motion analysis, the study differentiates dance steps based on the presence of emotion and identifies key movement features associated with emotional expression.]

**Writing Purpose Statements**This study aims to differentiate hip-hop dance steps based on whether joy is expressed and to explain their movement features using three-dimensional motion analysis. It focuses on the new jack swing, a popular hip-hop dance step, to understand how specific movements can convey emotions. The study seeks to provide objective measures for teaching and understanding emotional expression in dance. For example, the text reads: “This study aimed to differentiate hip-hop dance steps based on whether joy was expressed and explain their movement features using three-dimensional motion analysis.” (p. 1)”This study aimed to differentiate hip-hop dance steps based on whether there was an expression of joy and to explain their motion features using three-dimensional motion analysis.”(p. 10)

**Methodology and Data collection (Provide Evidence to Support the Credibility of the Study’s Findings)**This study utilized a qualitative research design with a focus on three-dimensional motion analysis. Ten participants with experience in hip-hop dance were instructed to perform an 80-second hip-hop dance, and the step performed after 60 seconds (new jack swing) was analyzed. The study used 34 variables related to time and space to capture and analyze the dancers’ movements. For example, the text reads: “Ten male and female participants (26.9 ± 12.8 years old) were instructed to perform an 80-second hip-hop dance, and the step performed after 60 s (new jack swing) was analyzed.” (p. 1) “To analyze the expressions of dancers during performance, the

participants were instructed to perform an 80-second hip-hop dance prepared specifically for the experiment. This dance was composed of various steps. To identify the expression during this dance performance of new jack swing, the step-dance as a staple of the dance was observed in the movement and, just after the 60-second mark, was analyzed” (p. 3). The study’s credibility is supported by its detailed methodology and the use of advanced motion analysis techniques. The findings are based on objective measurements of movement features, ensuring the reliability of the results. For example, the text reads: “The results revealed the following three points: The discriminant analysis, which was used to predict whether there was emotion, correctly classified cases at a rate of 70% or higher in four stages, which were motion phases.” (p. 10-11)

**Provide Evidence to Support the Transferability of the Study’s Findings**The study’s findings are transferable to other dance genres and educational contexts. The text mentions that the analyzed steps can be used in the practice of teaching dance, suggesting that the methods and findings could be applied to a broader range of dance education. For example, the text reads: “The analyzed steps can be used in the practice of teaching dance; however, a greater variety of hip-hop dance steps and a wider range of emotions need to be further examined in the future.” (p. 10)

**Provide Evidence to Support the Dependability of the Study’s Findings**The study used three-dimensional motion analysis to capture and analyze the dancers’ movements, providing objective measures of movement features. For example, the text reads: “The results revealed the following three points: The discriminant analysis, which was used to predict whether there was emotion, correctly classified cases at a rate of 70% or higher in four stages, which were motion phases.” (p. 8) The study also includes a detailed description of the variables used and the statistical methods applied, supporting the dependability of the findings. For example, the text reads: “When the differences in motion features by intensity were identified in Phase 2—the phase with the highest correct classification rate—significant differences were found in the mean of the height in the shoulders, the speed of the left acromion, the speed of the capitellum of the left elbow joint, the speed of the center of the left wrist joint, the speed of the lateral condyle of the left femur, the speed of the right acromion, the speed of the capitellum of the right elbow joint, and the flexion angle of the upper torso.” (p. 11)

**Provide Evidence to Support the Confirmability of the Study’s Findings**The authors reflect on the study’s findings and suggest future research directions. For example, the text reads: “Since the results of this study showed that the dance composed of steps differed according to whether there was an emotional expression, the hypothesis was supported.” (p. 10) The study’s findings are confirmed through the significant differences observed in movement features between dances performed with and without emotional expression. For example, the text reads: “Under Strong Emotion, the speed of the left elbow joint and the speed of the center of the left wrist joint had the highest correlation (r = 0.92).” (p. 11)

**Conclusion**
The study concludes that it is possible to express joy through hip-hop dance, particularly through the new jack swing. The findings indicate that emotional expression in dance is related mainly to the speed of the upper limbs. The study provides practical insights for dance education, suggesting that instructors can teach emotional expression through specific, objective movement features. Future research should explore a wider range of dance steps and emotions to further validate these findings.

**References(Use a Specific Style Referencing)**

Incorrectly written references:

Fischer DE (2013). Blackness, race, and language politics in Japanese hip hop. Transf. Anth. 21(2):135-152. http://doi/10.1111/traa.12017 DOI: 10.1111/traa.12017

**(...)**

[**Sample Number:** 19]

[**Title:** Krumpin’ In North Hollywood: Public Moves in Private Spaces]

[**Source:**https://doi.org/10.1525/boom.2013.3.1.1]

[**Edited?** No]

[**Reason for Inclusion:** This article examines the phenomenon of krump dancing in North Hollywood, focusing on how marginalized youth reclaim private spaces for public expression. It provides a detailed analysis of the cultural, social, and spatial dynamics of the 818 Session, a weekly krump gathering that repurposes a suburban parking lot as a site for community building and creative expression. The study highlights the intersection of race, space, and performance, offering insights into how krump dancers navigate and challenge dominant spatial politics in Los Angeles.]

[**Coding Date:**02/23/2025**]**

[**Coder:** First author]

[**Main points/impressions:**

Note. *The reference style of this dataset will be evaluated based on SAGE Harvard (https://journals.sagepub.com/author-instructions/IRS#ReferenceStyle)*

This article explores how krump dancers in North Hollywood transform private spaces into public arenas for artistic expression and community building. The study focuses on the 818 Session, a weekly gathering where dancers from diverse backgrounds come together to perform and share ideas. **]**

**Writing Purpose Statements**This article aims to analyze how krump dancers in North Hollywood repurpose private spaces for public expression, focusing on the cultural and social dynamics of the 818 Session. It seeks to understand how these dancers navigate racial and spatial politics in Los Angeles, using krump as a means to reclaim space and assert their identities. For example, the text reads: “The 818 Session offers alternative configurations and imaginings of the city. This is animated firstly through the session’s appropriation of public and semi-private spaces—abandoned lots, parks, churches, and currently the parking lot of a shopping center.” (p. 8)

**Methodology and Data collection (Provide Evidence to Support the Credibility of the Study’s Findings)**In this paper, krump dance is presented using a variety of data sources, including literature, videos, and movies.For example, the text reads: “...And since then it has grown into a national and international phenomenon within popular culture, crossing over into music videos, on US-based dance-competition television shows such as So You Think You Can Dance and America’s Next Best Dance Crew, as well in major film productions including Bring It On: All or Nothing and Step Up 3D. But it was Rize, video-director and photographer David LaChapelle’s 2005 documentary, that brought the dance style its most significant attention...” (p. 2) The study’s credibility is supported by its detailed descriptions and the use of multiple data sources, providing a rich and nuanced understanding of the krump community. For example, the text reads: “Several dancers insist that the lot’s architectural structure influences their creative process and physical movement; that is to say the lines, angles, shapes, colors, and textures of the buildings and terrain are a central part of their imaginative palette.” (p. 9)

**Provide Evidence to Support the Transferability of the Study’s Findings**
The study’s findings are transferable to other urban contexts where marginalized youth seek to reclaim public spaces for creative expression. The text mentions that the 818 Session’s practices of repurposing private spaces and challenging dominant spatial politics could be relevant to similar communities in other cities. For example, the text reads: “The 818 Session offers alternative configurations and imaginings of the city. This is animated firstly through the session’s appropriation of public and semi-private spaces—abandoned lots, parks, churches, and currently the parking lot of a shopping center.” (p. 8)

**Provide Evidence to Support the Dependability of the Study’s Findings**This article presents a detailed methodology and data analysis, ensuring the dependability of its findings. The researchers used ethnographic observations, interviews, and historical analysis to provide a comprehensive understanding of the 818 Session. For example, the text reads: “The dancing ignites. A male krumper assertively stomps the asphalt. He snakes in and out of the Toyota’s driver side window with his upper body. His arms whip at the air and his ankles twist side to side, rolling him up to his tiptoes.” (p. 2)

**Provide Evidence to Support the Confirmability of the Study’s Findings**
In this paper, the author does not conduct a reflective analysis, but utilizes the opinions of the characters in the text to reinforce the point of view,collective has no aversion to movement and change.For example, the text reads:‘‘Krump means life. Krump means elevation,he points out.’It means victory. It means prosperity. It means positivity. It means anger, emotion, oppression, inspiration,aspiration ... . It’s life. It’s energy. It’s forever. It’s what it is. It’s buck.’”(p. 15)

**Conclusion**
The article concludes that krump dancing in North Hollywood provides a powerful means for marginalized youth to reclaim private spaces for public expression. The study highlights the cultural significance of the 818 Session, where dancers come together to perform, share ideas, and build community. The findings suggest that krump offers a unique lens through which to understand the intersection of race, space, and performance in urban environments. Future research should explore similar practices in other cities and further investigate the role of krump in challenging dominant spatial politics.

**References(Use a Specific Style Referencing)**

This paper has only NOTES, not full references. For example, the records in the NOTE are:

Lefebvre, The Production of Space, 199.

Ibid., 26.

Widener, Black Arts West, 287

**(...)**

[**Sample Number:** 20]

[**Title:** Global Breakdancing and the Intercultural Body]

[**Source:**https://doi.org/10.2307/1478458.]

[**Edited?** No]

[**Reason for Inclusion:** This article explores the phenomenon of global breakdancing and its impact on the formation of the Intercultural Body. It provides a detailed analysis of how hip-hop culture, particularly breakdancing, transcends cultural boundaries and creates new forms of embodied identity. The study offers insights into the interplay between performance and performativity in the context of global hip-hop culture.]

[**Coding Date:** 02/23/2025**]**

[**Coder:** First author]

[**Main points/impressions:**

Note. *The reference style of this dataset will be evaluated based on SAGE Harvard (https://journals.sagepub.com/author-instructions/IRS#ReferenceStyle)*

This article examines how global breakdancing influences the formation of the Intercultural Body, highlighting the interdependence of performance and performativity. The study focuses on hip-hop culture in Hawai'i, where local and global influences intersect to create unique forms of embodied identity.**]**

**Writing Purpose Statements**
This article aims to investigate how global breakdancing influences the formation of the Intercultural Body, focusing on the interplay between performance and performativity. It seeks to understand how hip-hop culture, particularly breakdancing, transcends cultural boundaries and creates new forms of embodied identity. For example, the text reads: “I argue that transnational hip hop culture expands upon its basis in African American performance and poses new challenges to the once clear-cut paradigm of cultural appropriation of black dance and music by European-Americans.” (p. 31)

**Methodology and Data collection (Provide Evidence to Support the Credibility of the Study’s Findings)**This study utilizes a qualitative research design, combining ethnographic observations, interviews, and historical analysis. The author conducted field research in Hawai'i, observing hip-hop events and interviewing participants to gather insights into the cultural significance of breakdancing. For example, the text reads: “I conducted a high school study on the extent of hip hop culture among youth on the island of Hawai'i.” (p. 36) The study’s credibility is supported by its detailed descriptions and the use of multiple data sources, providing a rich and nuanced understanding of the hip-hop community. For example, the text reads: “The first dance group to perform was the Evolution Dancers, a six-member 'street dance' girl group, predominantly of Asian descent.” (p. 34)

**Provide Evidence to Support the Transferability of the Study’s Findings**
The study’s findings are transferable to other urban contexts where hip-hop culture thrives. The text mentions that the Intercultural Body concept can be applied to other global sites where hip-hop culture intersects with local traditions. For example, the text reads: “International competitions are held in Japan, Germany, and other global sites with participants from every continent.” (p. 42)

**Provide Evidence to Support the Dependability of the Study’s Findings**
This paper collected data through observation and interviews and analyzed the data to ensure the reliability of its findings. The authors used participant observation, interviews and document analysis to provide a comprehensive understanding of breakdancing globally.For example, the text reads: “The resulting Intercultural Body is dramatically illustrated through the prism of what I call hip hop's two-pronged bodily text.” (p. 39) The study also includes detailed descriptions of the dancers’ movements and the spatial dynamics of the events, supporting the dependability of the findings. For example, the text reads: “The democracy of the b-boy circle demonstrates how the individualism of dance styles, styles that speak, works together with good b-boy form, all rendering a cool Africanist aesthetic.” (p. 36)

**Provide Evidence to Support the Confirmability of the Study’s Findings**
The authors reflect on the study’s findings and suggest future research directions. For example, the text reads: “Global breakdancing is a potentially subversive means of culturally transgressing the nation-state, as well as transcending the controlling and racializing aspects of capitalism.” (p. 42) The study’s findings are confirmed through the detailed observations and interviews with participants, highlighting the cultural significance of breakdancing and its role in forming the Intercultural Body. For example, the text reads: “The Intercultural Body is where 'natural' appropriation can take place on the street and in the clubs by practitioners of all nationalities drawn by the powerful improvisatory Africanist aesthetic.” (p. 42)

**Conclusion**
The article concludes that global breakdancing offers a unique lens through which to understand the formation of the Intercultural Body. The study highlights the interdependence of performance and performativity in hip-hop culture, demonstrating how breakdancing transcends cultural boundaries and creates new forms of embodied identity. Future research should explore similar practices in other cities and further investigate the role of hip-hop culture in challenging dominant cultural paradigms.

**References(Use a Specific Style Referencing)**

Works Cited was used for this paper, and the reference list does not include some of the elements that meet specific formatting requirements.

Butler, Judith. 1990a. Gender Trouble: Feminism and the Subversion ofldentity. New York: Routledge._______and Joan W, Scott, eds. 1990b. Feminists Theorize the Political. New York: Routledge._______.1996.“Performativity's Social Magic." In The Social and Political Body. Edited byTheodore R. Schatzki and Wolfgang Natter, 29-48. New York: Guilford Press.

(...)

[**Sample Number:** 21]

[**Title:** The importance of creativity in the sportification of breakdance]

[**Source:**https://doi.org/ 10.3389/feduc.2022.855724]

[**Edited?** No]

[**Reason for Inclusion:**This article was chosen because it gives a detailed description of the history and current state of breakdancing. This provides a valuable theoretical basis for the study of the street dance framework. The article explores the role of creativity in the evolution and sportification of breakdancing, particularly in the context of its inclusion in the 2024 Paris Olympics. The study highlights how creativity functions as both a performance strategy and a judging criterion in competitive breakdancing, underscoring its importance for both technical execution and artistic expression.]

[**Coding Date:** 02/23/2025**]**

[**Coder:** First author]

[**Main points/impressions:**

Note. *The reference style of this dataset will be evaluated based on SAGE Harvard (https://journals.sagepub.com/author-instructions/IRS#ReferenceStyle)*

This article addresses the growing tension between creativity and standardization as breakdancing transitions from an underground street dance form to a regulated competitive sport. While breakdancing has traditionally emphasized improvisation, musicality, and individual expression, the sportification process introduces structured rules and scoring systems that may challenge the creative core of the dance. The authors argue that maintaining creativity is essential for preserving the cultural authenticity of breakdancing and ensuring that it remains a dynamic and expressive art form.]

**Abstract**

This study explores the impact of sportification on breakdancing, focusing on the tension between maintaining creative expression and meeting standardized judging criteria in competitive settings. The article examines the evolution of breakdancing from an underground dance style to a competitive sport recognized by the International Olympic Committee (IOC). Creativity, defined as the ability to produce original and expressive movements, is identified as a key factor in competitive success. The study argued that creativity must be preserved in breakdancing competitions to ensure that the art form retains its core identity as a form of cultural expression.

**Writing Purpose Statements**

This article is a literature review, and although it does not detail the locations of the participants, However, it is mentioned in the reference that "The two authors of this article visited many breakdance studios in China. They shared their teaching methods with many breakdance teachers. An interesting finding was that the execution of the dance movements demonstrated by almost all the students was similar; even the emotions and facial expressions were highly similar. "(p.2 ) “Secondly, in terms of little-c creativity (creativity that is recognized as such by someone else), breakdance teachers should encourage and support children to explore the integration of dance movements with personal emotional expression and provide materials that can be choreographed because the focus of the creative dance is to explore the possibilities of personal, expressive movement. Foley’s (2016) baby project gives an excellent example of our argument; for instance, breakdance teachers can encourage children to show like superheroes during the dance, making the dance moves more dynamic.During the improvisation process, teachers should give positive encouragement and feedback, regardless of whether the children’s presentation is good or bad (e.g., lack of fluency in movement and anxiety). Similarly, Starko (1999) argued that finding a problem, identifying a question to answer, or investigating an area can be a crucial component of creative productivity”(p.4). Can provide a reference to describe the participants of the study or the study location.

**Methodology and Data collection(Provide Evidence to Support the Credibility of the Study’s Findings)**

In this article, the author cites several breakdancing scholars or hip-hop scholars to illustrate the role of creativity.

Data Collection Methods: Literature Review qnalysis of existing research on breakdancing, sportification, and creativity in dance.

**Provide Evidence to Support the Transferability of the Study’s Findings**

This paper provides a detailed description of the findings, but does not explain the sampling strategy or discuss how the findings resonate with existing literature in different contexts. A detailed description of the findings can be found in:“Secondly, in terms of little-c creativity (creativity that is recognized as such by someone else), breakdance teachers should encourage and support children to explore the integration of dance movements with personal emotional expression and provide materials that can be choreographed because the focus of the creative dance is to explore the possibilities of personal,expressive movement. Foley’s (2016) baby project gives an excellent example of our argument; for instance, breakdance teachers can encourage children to show like superheroes during the dance, making the dance moves more dynamic. During the improvisation process, teachers should give positive encouragement and feedback, regardless of whether the children’s presentation is good or bad (e.g., lack of fluency in movement and anxiety). Similarly, Starko (1999) argued that finding a problem,identifying a question to answer, or investigating an area can be a crucial component of creative productivity.”(p.4)

**Results and Discussion(Provide Evidence to Support the Dependability of the Study’s Findings)**

By reading the article, this article does not mention following the saturation principle of data collection, but provides iterative data collection.This article discussed the advantages and examples of four C-modes, and proposes a set of methods for breakdancing teachers to cultivate students' creativity. The original text is as follows: “Kaufman and Beghetto (2009) define creativity as having four levels: Mini-C creativity (subjectively recognized creativity),Little-C creativity (creativity recognized from others), ProC creativity (creativity classified as a novel and significant contribution by experts in the area), and Big-C creativity (major creative achievements that only a few people can achieve)...a set routine to choreograph, pupils may not collaborate. Therefore, breakdance teachers can support children to work in groups and share and exchange ideas,laying the foundation for developing children’s mini-c creativity(subjectively recognized creativity).”(p.3-4)

**Provide Evidence to Support the Confirmability of the Study’s Findings**

“A limitation of this article is that it does not adequately describe the divergence between breakdance as a product of the American hip-hop cultural movement and other national cultures.”(p.4)

**Conclusion**

The study concludes that creativity remains a defining element of breakdancing despite the challenges posed by sportification. While formal rules and scoring systems provide a framework for competitive evaluation, the authors emphasize that preserving the creative and expressive nature of breaking is essential for maintaining its authenticity and cultural significance. The authors advocate for a balanced approach that allows for both competitive structure and creative freedom to ensure that breakdancing retains its unique identity within the global sports and hip-hop communities.

**References(Use a Specific Style Referencing)**

Bergmann, S. (1992). The process/product dichotomy and its implications for creative dance. J. Aesthetic Educat. 26, 103–108. doi: 10.2307/3332928

Blagojevic, G. (2009). Belgrade breakdance girl: Breaking gender-specific ´stereotypes with dance. CHAY 57, 19–24. doi: 10.2298/gei0902019b

Bode Bakker, M., and Nuijten, M. (2018). ‘When breaking you make your soul dance’Utopian aspirations and subjective transformation in breakdance. Identities 25, 210–227. doi: 10.1080/1070289X.2017.1400275

Breaking history (2021). KENQ13 SWIFT (7Gems) 2018 V1 Battle of the Gods Recap (2018). [Video]. YouTube. Available online at: https://www.youtube.com/watch?v=u5NxDTuZtrw (accessed March 19, 2021).

(...)

[**Sample Number:** 22]

[**Title:** Injury incidence in hip hop dance]

[**Source:** https://doi.org/10.1111/j.1600-0838.2010.01173.x]

[**Edited?** No]

[**Reason for Inclusion:** This study investigates the injury incidence and patterns among hip hop dancers, focusing on three main categories: Breakers, Popper/Lockers, and New Schoolers. It provides valuable insights into the prevalence and types of injuries sustained by these dancers, highlighting the need for better injury prevention and education in this population.]

[**Coding Date:** 02/24/2025**]**

[**Coder:** First author]

[**Main points/impressions:**

Note. *The reference style of this dataset will be evaluated based on SAGE Harvard (https://journals.sagepub.com/author-instructions/IRS#ReferenceStyle)*

This study examines the injury incidence and patterns among hip hop dancers, particularly focusing on Breakers, Popper, and Lockers. The findings reveal that hip hop dancers, especially Breakers, experience high rates of injuries, particularly in the upper and lower extremities. ]

**Writing Purpose Statements**
This study aims to determine the injury incidence and patterns among three groups of hip hop dancers: Breakers, Popper/Lockers, and New Schoolers. It seeks to understand the prevalence and types of injuries sustained by these dancers and to compare injury patterns between different dance styles. For example, the text reads: “We determined injury incidence and patterns in a subset of hip hop dancers (Breakers, Popper, and Lockers), using standardized injury reporting guidelines” (p. 347).“Survey participants were recruited at battles, dance conferences, clubs, and on dance related web sites within the United States and internationally”(p. 348).

**Methodology and Data collection (Provide Evidence to Support the Credibility of the Study’s Findings)**
This study utilized a web-based survey to collect data from 312 intermediate, advanced, and expert hip hop dancers over a 6-month period. The survey included questions on demographics, dance experience, and injury history. For example, the text reads: “Data were collected for 5 months using a secure web-based survey (Survey Monkey, Portland, Oregon, USA) with 45 multiple choice and open-ended questions.” (p. 348) The study’s credibility is supported by its detailed methodology and the use of standardized injury reporting guidelines. The findings are based on a large sample size and rigorous statistical analysis. For example, the text reads: “Separate analysis of variances were used to compare injuries by gender, age category, experience level, and dance style in SPSS (Version 15.0, Chicago, Illinois, USA).” (p. 348)

**Provide Evidence to Support the Transferability of the Study’s Findings**
The study’s findings are transferable to other populations of hip hop dancers, particularly those involved in competitive and street dance styles. The text mentions that the injury rates and patterns observed in this study are relevant to other dance forms and sports with high physical demands. For example, the text reads: “Hip hop dancers report injury rates that are higher than other dance forms but similar to gymnastics.” (p. 347)

**Provide Evidence to Support the Dependability of the Study’s Findings**The study used a secure web-based survey and standardized injury reporting guidelines to collect and analyze data. For example, the text reads: “Injury incidence was calculated by dividing the number of injuries by the total number of subjects.” (p. 348) The study also includes detailed demographic information and injury patterns, supporting the dependability of the findings. For example, the text reads: “Breakers had higher rates of upper extremity injuries compared with Popper/Lockers and New Schoolers (Po0.01) regardless of the injury definition used.” (p. 349)

**Provide Evidence to Support the Confirmability of the Study’s Findings**
The authors reflect on the study’s findings and suggest future research directions. For example, the text reads: “With the increase in participation and documented high rates of preventable injuries, education about injury prevention and use of protective equipment is warranted.” (p. 353) The study’s findings are confirmed through the detailed analysis of injury incidence and patterns, highlighting the high rates of injuries among hip hop dancers. For example, the text reads: “Annual injury incidence was 237% (162% involving TL). Lower extremity injuries were 52% and upper extremity injuries 32% of total injuries.” (p. 348)

**Conclusion**The study concludes that hip hop dancers, particularly Breakers, experience high rates of injuries, especially in the upper and lower extremities. The findings highlight the need for better injury prevention and education in this population. Future research should focus on developing targeted interventions to reduce injury rates and improve the overall health and safety of hip hop dancers.

**References(Use a Specific Style Referencing)**

Bowling A. Injuries to dancers: prevalence, treatment, and perceptions of causes.

BMJ 1989: 298(6675): 731–734.

Kramer N, Cooper M. We B* Girlz Da Book, 2009. Available at http:// www.bgirlz.com/ (accessed March 20,2008).

Okumura K. Styles, 1992. Available at http://www.msu.edu/user/okumurak/

styles/styles.html (accessed March 13, 2008)

(...)

[**Sample Number:** 23]

[**Title:** From street dance to hip hop: performance as a tactic to maintain cultural meanings]

[**Source:** https://doi.org/10.5324/da.v7i1.4226]

[**Edited?** No]

[**Reason for Inclusion:** This article explores the historical development and cultural significance of street dance and hip hop in Brazil, particularly in the city of Uberlândia. It provides valuable insights into how street dance evolved into hip hop performance as a means to preserve cultural identity and resist dominant artistic norms. The study offers a detailed analysis of the social and aesthetic transformations in dance practices, highlighting the importance of maintaining cultural meanings through performance.]

[**Coding Date:** 02/24/2025**]**

[**Coder:** First author]

[**Main points/impressions:**

Note. *The reference style of this dataset will be evaluated based on SAGE Harvard (https://journals.sagepub.com/author-instructions/IRS#ReferenceStyle)*

This article examines the transition from street dance to hip hop performance in Brazil, focusing on the city of Uberlândia. It highlights how hip hop emerged as a means to preserve cultural meanings and resist dominant artistic norms. ]

**Writing Purpose Statements**
This article aims to historicize the concept of street dance and analyze its transition to hip hop performance in Brazil. It seeks to understand how hip hop emerged as a cultural tactic to maintain and preserve the meanings and practices of street dance. For example, the text reads: “Analysing street dance and hip hop considering their ruptures and continuities, the text intends to contribute to studies dedicated to the presence of dance in the construction of urban identities.” (p. 45)

**Methodology and Data collection (Provide Evidence to Support the Credibility of the Study’s Findings)**This study utilizes a historical and ethnographic approach, combining interviews and archival research. The author conducted interviews with dancers and practitioners in Uberlândia to understand the cultural meanings and practices of street dance and hip hop. For example, the text reads: “I use interviews carried out between 2005 and 2008 for my master’s study in social history at Federal University of Uberlândia. Dancers who were active between the late 1970s and the first decade of the 21st century were interviewed.” (p. 47) The study’s credibility is supported by its detailed methodology and the use of oral history techniques to capture the lived experiences of dancers. For example, the text reads: “These dancers provided reports and explanations regarding how they understood the dance they practiced, and the methodological procedure was guided by the oral history.” (p. 47)

**Provide Evidence to Support the Transferability of the Study’s Findings**The study’s findings are transferable to other urban contexts where street dance and hip hop have evolved. The text mentions that the cultural dynamics observed in Uberlândia can be seen in other cities where breaking emerged as a means to resist dominant artistic norms. For example, the text reads: “I also intend to contribute to the understanding that the strengthening and expansion of breaking on a global level is a result of complex cultural relations when viewed from a local standpoint, in this case, from a city in the interior of Brazil” (p. 47). “The strengthening and expansion of breaking on a global level is a result of complex cultural relations when viewed from a local standpoint, in this case, from a city in the interior of Brazil.” (p. 56)

**Provide Evidence to Support the Dependability of the Study’s Findings**The author uses interviews to provide a comprehensive understanding of the transition from street dance to hip hop. For example, the text reads: “The practice of hip hop – taking breaking as an example – has always been understood as something aesthetically distinct from street dance.” (p. 56) The study also includes detailed descriptions of the cultural and social dynamics of the dance practices, supporting the dependability of the findings. For example, the text reads: “The main change in street dance which now will be discussed is the construction of new meanings. Such meanings both relate to expectations and are motivations for dancing.” (p. 52)

**Provide Evidence to Support the Confirmability of the Study’s Findings**The authors reflect on the study’s findings and suggest future research directions. For example, the text reads: “Despite the different definitions that the concept of street dance receives in the available literature, it can be understood that this variety of definitions is due to the absence of historical analysis on the specifics of this concept and the dance practice that it designates.” (p. 59) The study’s findings are confirmed through the detailed analysis of the cultural and social dynamics of street dance and hip hop, highlighting the importance of maintaining cultural meanings through performance. For example, the text reads: “There are differences that stress the formal distance between street dance and hip hop. However, street dancers and street dance groups refused other interpretations to be placed on them and what the dance market presented, all that coming from the dialogue with specialized critics. In this way the street dance dancers found in breaking practice a way to maintain, share, and perform cultural customs. This situation can be read as “a rebellion against institutions and representations that become ’un-believable’. It’s a refusal to the un-signified” (Certeau, 2005, p. 33, my translation). Such a refusal can be seen in identification symbols, in the distinctive aspects to tell the best group from the others, and in the belief shared by the subjects who practice certain dances or who share a theory-methodology on dance that might be different from that held by the so-called “dance specialists.” (p. 60)

**Conclusion**
The article concludes that hip hop emerged as a cultural tactic to maintain and preserve the meanings and practices of street dance. The study highlights the importance of performance in preserving cultural identity and resisting dominant artistic norms. Future research should explore similar practices in other cities and further investigate the role of hip hop in maintaining cultural meanings.

**References(Use a Specific Style Referencing)**

Correia, A., Silva, C., & Ferreira, N. (2017). Do racha na rua à batalha no palco: Cenas das danças urbanas [From street battles to stage battles: Scenes from urban dances]. Motrivivência, 29(50), 213-231.

Lauxen, P., & Isse, S. (2009). Contextos de dança de rua: Um pouco de história e práticas docentes [Street dance contexts: A little history and teaching practices]. Revista Destaques Acadêmicos, 1(2), 69-78.

(...)

[**Sample Number:** 24]

[**Title:** Jookin, Jiggin, Beatin Ya Feet and Gettin'Light: African-American Footwork Traditions]

[**Source:**https://digitalcommons.lmu.edu/cgi/viewcontent.cgi?article=1029&context=dance_students]

[**Edited?** No]

[**Reason for Inclusion:** This paper examines the regional variations and shared characteristics of African-American footwork traditions, specifically focusing on Memphis jookin, New Orleans jiggin, Washington DC’s beat ya feet, and New York City’s litefeet. It highlights the dances’ connections to older African and African-American aesthetics and argues that these dances act as important cultural institutions preserving specific socio-cultural practices.]

[**Coding Date:** 02/24/2025**]**

[**Coder:** First author]

[**Main points/impressions:**

Note. *The reference style of this dataset will be evaluated based on SAGE Harvard (https://journals.sagepub.com/author-instructions/IRS#ReferenceStyle)*

This paper explores the regional footwork traditions of African-American dance, emphasizing their shared cultural heritage and connections to older dance aesthetics.]

**Writing Purpose Statements**This paper aims to examine the regional variations and shared characteristics of African-American footwork traditions, specifically focusing on Memphis jookin, New Orleans jiggin, Washington DC’s beat ya feet, and New York City’s litefeet. It seeks to highlight the dances’ connections to older African and African-American aesthetics and argue that these dances act as important cultural institutions preserving specific socio-cultural practices. For example, the text reads: “This paper argues that these dances form a singular body of work that is directly connected to African-American dance traditions developed during and after enslavement.” (p. 2)

**Methodology and Data collection (Provide Evidence to Support the Credibility of the Study’s Findings)**This study utilizes a historical and comparative approach, analyzing the characteristics of each dance style and their connections to older African and African-American dance traditions. The author examines various sources, including academic literature, dance videos, and historical records, to provide a comprehensive understanding of these regional dance styles. For example, the text reads: “This paper looks specifically at Memphis jookin, New Orleans jiggin, Washington DC’s beat ya feet and New York City’s litefeet. It explores common characteristics and regional differences in each dance, connecting them to older African and African-American dance aesthetics.” (p. 3) The study’s credibility is supported by its detailed methodology and the use of multiple data sources, providing a rich and nuanced understanding of the cultural significance of these dances. For example, the text reads: “The oldest of these was the ring shout—a circle dance done in West Africa.” (p. 3)

**Provide Evidence to Support the Transferability of the Study’s Findings**The study’s findings are transferable to other regions with established African-American communities where similar dance styles may have evolved. The text mentions that nearly every region with an established African-American community has a unique dance style, suggesting that the cultural dynamics observed in this study could be relevant to other areas. For example, the text reads: “Nearly every region with an established African-American community has a unique dance style, but this paper will look most in depth at Memphis jookin, DC’s beat ya feet, Louisiana or New Orleans jiggin’ and New York City’s litefeet.” (p. 3)

**Provide Evidence to Support the Dependability of the Study’s Findings**
The author uses historical analysis and comparative methods to provide a comprehensive understanding of the regional footwork traditions. For example, the text reads: “These dances are often hyper-regional and unique to the traditions of these areas, but they also share many of the same cultural characteristics.” (p. 3) The study also includes detailed descriptions of each dance style and their cultural significance, supporting the dependability of the findings. For example, the text reads: “Memphis jookin’ is characterized by smooth and precise footwork. Sometimes referred to as ‘street ballet,’ Memphis jookin’ originated in Memphis’ hip-hop scene in the 1990s.” (p. 4)

**Provide Evidence to Support the Confirmability of the Study’s Findings**
The authors did not reflect on the results of the study, but only summarized them.For example, the text reads: “When looked at together, regional African-American social dances embody many tenets of African-American culture and history. Each uniquely influenced by the Black populations of their respective cities, these dances carry on older African and African-American dance aesthetics, like polyrhythms, both in music and in dance, and complex footwork.” (p. 10) The study’s findings are confirmed through the detailed analysis of the cultural and social dynamics of these dance practices, highlighting the importance of maintaining cultural meanings through performance. For example, the text reads: “These dances both legitimize African American and Black culture in the United States and act as living museums of African-American traditions.” (p. 3)

**Conclusion**
The article concludes that regional African-American footwork traditions, such as Memphis jookin, New Orleans jiggin, Washington DC’s beat ya feet, and New York City’s litefeet, share common characteristics and are deeply connected to older African and African-American dance aesthetics. These dances act as important cultural institutions, preserving specific socio-cultural practices and highlighting the importance of community involvement. Future research should explore similar practices in other regions and further investigate the role of these dances in maintaining cultural heritage.

**References(Use a Specific Style Referencing)**

Theoretical sources in this paper are represented as NOTE and BIBLIOGRAPHY and do not include elements in the reference list that meet specific formatting requirements (e.g., APA, MLA, Chicago style) such as the full title, names of all authors (last name + initials), name of the journal, year of publication, volume number, and page number.

@StreetwizefoundationSWF “Street Wize Foundation (SWF),” Facebook, https://www.facebook.com/StreetwizefoundationSWF/.

Wreckin’ Shop from Brooklyn, Dian Martel, New York City: 1992, <https://www.youtube.com/watch?v=zUAuCQN-AJI.>

(...)

[**Sample Number:** 25]

[**Title:** Extreme kinematics in selected hip hop dance sequences]

[**Source:**https://www.jstor.org/stable/10.2307/48714269]

[**Edited?** No]

[**Reason for Inclusion:** This study investigates the extreme kinematics of selected hip hop dance sequences, comparing the angular displacements in breakdance, house, and toprock. It provides valuable insights into the biomechanical demands of these dance styles and their potential impact on injury rates. The findings highlight the high joint angles and extreme movements involved in hip hop dance, contributing to the understanding of injury mechanisms in this population.]

[**Coding Date:** 02/24/2025**]**

[**Coder:** First author]

[**Main points/impressions:**

Note. *The reference style of this dataset will be evaluated based on SAGE Harvard (https://journals.sagepub.com/author-instructions/IRS#ReferenceStyle)*

This study examines the extreme kinematics of selected hip hop dance sequences, focusing on breakdance, house, and toprock. The findings reveal that these dance styles involve high joint angles and extreme movements, which may explain the high injury rates reported in hip hop dancers. ]

**Writing Purpose Statements**This study aims to compare the angular displacements in selected hip hop dance sequences, specifically focusing on breakdance, house, and toprock. It seeks to understand the biomechanical demands of these dance styles and their potential impact on injury rates. For example, the text reads: “The purpose of this study was to compare representative hip hop steps found in breakdance (toprock and breaking) and house and provide descriptive statistics of the angular displacements that occurred in these sequences.” (p. 126)

**Methodology and Data collection (Provide Evidence to Support the Credibility of the Study’s Findings)**This study utilized a biomechanical approach, collecting kinematic data from six expert female hip hop dancers performing choreographed sequences of breakdance, house, and toprock. The dancers performed multiple steps within each sequence, and the data were captured using a motion capture system. For example, the text reads:“Six expert female hip hop dancers, experienced in breaking and house dancing”（p.127）. “Hip, knee, and ankle kinematics were collected during sequences that were 18 to 30 sec long. Hip, knee, and ankle three-dimensional peak joint angles were compared in repeated measures ANOVAs with post hoc tests where appropriate (p<0.01).” (p. 126) The study’s credibility is supported by its detailed methodology and the use of advanced motion capture technology to measure joint angles. For example, the text reads: “Kinematic data were collected using a 6-camera motion capture system (Vicon Nexus, Oxford Metrics Ltd, Oxford, UK) at a sampling rate of 120 Hz.” (p. 128)

**Provide Evidence to Support the Transferability of the Study’s Findings**The study’s findings are transferable to other populations of hip hop dancers, particularly those involved in breakdance, house, and toprock. The text mentions that the extreme joint angles and movements observed in these dance styles may contribute to high injury rates, suggesting that similar biomechanical demands could be found in other hip hop dance populations. For example, the text reads: “Hip hop dancers work at weight-bearing joint end ranges where muscles are at a functional disadvantage. These results may explain why lower extremity injury rates are high in this population.” (p. 126)

**Provide Evidence to Support the Dependability of the Study’s Findings**The study used a motion capture system to collect kinematic data and compared joint angles across different dance sequences. For example, the text reads: “Peak angles of the breaking sequence, which included floorwork, exceeded the other two sequences in the majority of planes and joints.” (p. 126) The study also includes detailed descriptions of the dance sequences and the statistical methods used, supporting the dependability of the findings. For example, the text reads: “Separate repeated measures in general linear models (GLM) were used for each joint (hip, knee, ankle) for the dependent variables (3-D plane peak angle) in SPSS (SPSS v. 21, IBM Corp, Armonk, NY).” (p. 128)

**Provide Evidence to Support the Confirmability of the Study’s Findings**The authors reflect on the study’s findings and suggest future research directions. For example, the text reads: “Further investigations into training regimes that may reduce injuries are warranted. Hip hop dancers are a neglected population, sustaining high rates of injuries that may result from these biomechanical stressors.” (p. 133)

**Conclusion**The article concludes that hip hop dance, particularly breakdance, involves extreme joint angles and movements that place significant biomechanical demands on the body. These findings may explain the high injury rates reported in hip hop dancers. Future research should explore the kinetics of hip hop dance and investigate training regimes that can reduce injury rates. The study highlights the need for better understanding and support for hip hop dancers, who face significant biomechanical challenges in their performances.

**References(Use a Specific Style Referencing)**

United States Bone and Joint Initiative. Musculoskeletal Injuries.The Burden of Musculoskeletal Diseases in the United States, 2nd ed.Rosemont, IL: American Academy of Orthopaedic Surgeons;2011: pp129–79.

Kumar S. A conceptual model of overexertion, safety, and risk of injury in occupational settings. Hum Factors. 1994;36(2):197–209.

(...)

[**Sample Number:** 26]

[**Title:** C'mon to my house: Underground-House Dancing]

[**Source:**https://doi.org/10.1080/10.2307/1477805 ]

[**Edited?** No]

[**Reason for Inclusion:** This article provides a comprehensive exploration of the Underground-House dancing scene, focusing on its cultural significance, historical roots, and the unique social dynamics that define it. It offers valuable insights into how Underground-House has evolved over the past three decades and its influence on contemporary dance culture. The article also highlights the importance of the "vibe" and the communal experience, which are central to the essence of Underground-House. Additionally, it discusses the impact of the post-9/11 environment on the resurgence of interest in the nostalgic and utopian ethos of the 1970s and 1980s dance culture.]

[**Coding Date:** 02/25/2025**]**

[**Coder:** First author]

[**Main points/impressions:**

Note. *The reference style of this dataset will be evaluated based on SAGE Harvard (https://journals.sagepub.com/author-instructions/IRS#ReferenceStyle)*

This article delves into the cultural and social dimensions of Underground-House dancing, emphasizing its role in creating a sense of "communitas," as described by anthropologist Victor Turner. The study highlights how Underground-House clubs and dance practices represent unique, contemporary "liminoid" rites of passage, with vivid stages of separation and liminality.]

**Writing Purpose Statements**

This article explores the cultural and social significance of Underground-House dancing, emphasizing its role in creating a sense of community and liberation through dance. It examines how Underground-House has evolved over the past three decades and its influence on contemporary dance culture. The study highlights the importance of the "vibe" and the communal experience, which are central to the essence of Underground-House. For example, the text reads: “The phenomenon of Underground-House clubs and dance practices represents unique, contemporary, 'liminoid' rites of passage, with vivid stages of separation and liminality” (Sommer, 2001, p. 72). Additionally, the article discusses the impact of the post-911 environment on the resurgence of interest in the nostalgic and utopian ethos of the 1970s and 1980s dance culture, as seen in the increased attendance at clubs: “As anecdotal evidence, Archie Burnett, Chris Buxenbaum, and Brahams La Fortune all said they were noticing an increase in club attendance since '911' (i.e., terrorist attacks of September 11, 2001). As Burnett observed, 'People need to bust out more than ever, now'” (Sommer, 2001, p. 84).

**Methodology and Data collection (Provide Evidence to Support the Credibility of the Study’s Findings)**

This article utilized a qualitative research design, drawing on extensive field observations, interviews, and historical analysis of the Underground-House scene. The author, Sally R. Sommer, has been conducting research on social dance and clubs since the mid-1970s, with a specific focus on the New York City Underground-House scene since 1992. Data were collected through long-term participant observation, video documentation, and in-depth interviews with key figures in the dance community. For example, the text reads: “The information in this article is based on my research on social dance and the clubs that has been ongoing since the mid-1970s. More specific to this essay is the research done since 1992, which has concentrated on a group of dancers from the Underground-House scene in New York City who were the main subjects of an hour-long video documentary, Check Your Body at the Door” (Sommer, 2001, p. 84).

**Provide Evidence to Support the Transferability of the Study’s Findings**

The study’s findings are supported by the detailed descriptions of the Underground-House scene in New York City, which have broader implications for understanding similar dance cultures globally. The article highlights the universal appeal of the "vibe" and the communal experience in dance communities. For example, the text reads: “The vibe is an active communal force, a feeling, a rhythm that is created by the mix of dancers, the balance of loud music, the effects of darkness and light, the energy. Everything interlocks to produce a powerful sense of liberation” (Sommer, 2001, p. 73).

**Provide Evidence to Support the Dependability of the Study’s Findings**

The article presents a consistent and detailed account of the Underground-House scene, supported by multiple interviews and observations over several years. The author’s long-term engagement with the dance community ensures the reliability and depth of the findings. For example, the text reads: “All of the dancers are people the author has known for years (some for as many as twenty, others for as few as six)” (Sommer, 2001, p. 84).

**Provide Evidence to Support the Confirmability of the Study’s Findings**

The author reflects on the study’s limitations and the evolving nature of the dance culture. The article acknowledges the impact of historical and social contexts on the Underground-House scene. For example, the text reads: “Yet in many ways Underground-House is an archaic throwback. Rather than being radical in production and ethos, it is very conservative in its structures, idealistic even” (Sommer, 2001, p. 83).

**Conclusion**

The article concludes that Underground-House dancing is not just a form of entertainment but a cultural movement that fosters a sense of community and liberation. It highlights the importance of maintaining the authenticity and inclusivity of the dance culture while adapting to modern contexts. The study provides valuable insights for dance scholars, cultural historians, and anyone interested in understanding the social dynamics of dance communities. The integration of historical context, participant observations, and interviews offers a rich and nuanced perspective on the Underground-House scene.

**References (Use a Specific Style Referencing)**

In this paper, notes and work cited were used to document the literature, and not all of the elements that meet specific formatting requirements (e.g., APA, MLA, Chicago style) were included in the reference list, such as the full title, names of all authors (last name + initials), journal name, year of publication, volume number, and page number.

Owen, Frank,1997.“Crackdown in Clubland," Village Vice, February 18: 38-39______.2001.“Private Pandemonium: As Clubs Flounder, Clandestine House Parties Flourish,Village Voice, July 17: 37, 38.

Reynolds, Simon. 2001. “Disco Double Take: New York Parties Like It's 1975," Village Voice, July 17:43-44.

(...)
