# Supplementary material for: Street dance redefined: a bridge across the knowledge gap
Source: Front Sports Act Living. 2025 Jul 23;7:1610656. doi: 10.3389/fspor.2025.1610656 (PMC12325215; doi:10.3389/fspor.2025.1610656)
Supplement: Supplementary file 3 [file Supplementaryfile3.docx]

Supplementary Material C – Researcher’s Daily Reflections(excerpt)

**January 15, 2025**

To date, I have published seven articles related to street dance, a monograph on street dance. In the process of my searching for literature to write my articles, I have discovered that the terminology in the field of street dance has different styles of names and there is no unified terminology specification and definition. This situation that occurred may seem insignificant, but it has begun to have an impact on the theoretical basis of street dance researchers, at least in my opinion, the lack of uniform standards of street dance theory research will be biased, for example, breakdance and breaking are two styles, if the researcher is biased in the definition of the researchers will affect the scope of the study and the selection of the research object, resulting in a research errors in the results.

This confusing cognitive generation gap of definitions led me to decide to do an integrative study of street dance theoretical frameworks and their definitions. Through the establishment of the street dance theoretical framework and the reconstruction of definitions, I hope to help street dance researchers establish a unified standard to reduce the bias in definitions by street dance researchers, thus increasing the credibility and reliability of street dance research.

**January 17, 2025**

At a conference not long ago, my mentor K. re-emphasized the importance of using more precise terminology in the study of street dance theory. He pointed out the importance of using specific street dance style names, such as Breaking, Popping, Locking, or Krumping, rather than simply using the broad term “street dance” to summarize all styles. In the subsequent Daily seminar, I consulted with Assistant Professor C, who also reiterated the need for precise terminology. Combined with her experience as a reviewer, she pointed out that the use of broad terms is a common but uncritical practice. Using street dance research as an example, she explained the importance of the need for clear definitions of street dance, such as the definitions of different street dance styles, rather than using generalizations such as “street dance” or “hip-hop dance”. Similarly, in street dance research, using only “street dance” to refer to all styles can lead to a lack of clarity in the research design, making it difficult for readers to understand the specific research methodology and theoretical framework used by the researcher. Therefore, in street dance theory research, clearly distinguishing between different styles and adopting precise terminology are key to ensuring research rigor and academic standardization.

After reviewing 26 theoretical studies on the field of street dance, I noticed a strange phenomenon. Some researchers have only explained some of the styles of street dance (e.g., Breaking, Popping, or Locking), but no one has examined the fragmented styles of street dance in the context of the entire theoretical framework of street dance. In addition, there are significant differences in the understanding of street dance styles among scholars of different styles, and the reasons for this cognitive generation gap are seldom explored in depth. This obviously confuses readers after reading. In articles that do not clearly categorize street dance styles, it sometimes looks like an in-depth study of a single street dance style, sometimes a hybrid exploration of multiple street dance styles, or perhaps even a misperceived study due to confusion of styles.

[...]

For example, one study may focus on the cultural context and assessment research of Breaking, while another may explore the technical details and assessment of body control in Popping, but these studies often fail to situate their respective findings within a unified theoretical framework of street dance. This lack of a holistic perspective not only limits the depth and breadth of theory, but may also lead readers to misunderstand the diversity and complexity of street dance. More importantly, scholars of different styles have divergent definitions and classification criteria for street dance, and this cognitive difference further exacerbates the fragmentation of theoretical research.

For new researchers who are unfamiliar with the differences between street dance styles such as Breaking, Popping, and Locking, it may be difficult for them to accurately identify which style or combination of styles the article describes as “street dance research”. Of course, it should also be recognized that some researchers, while not explicitly identifying the street dance styles studied in their articles, may still have an explicit framework for their research. These frameworks may consist of one or several street dance styles. This requires an experienced researcher to scrutinize the article and make a judgment. However, this process can be very labor intensive for the reader. So how can an incomplete categorization of street dance styles represent a complete street dance theory?
